# Gene-expression profiling of bortezomib added to standard chemoimmunotherapy for diffuse large B-cell lymphoma (REMoDL-B): an open-label, randomised, phase 3 trial

**DOI:** 10.1016/S1470-2045(18)30935-5

**Published:** 2019-05

**Authors:** Andrew Davies, Thomas E Cummin, Sharon Barrans, Tom Maishman, Christoph Mamot, Urban Novak, Josh Caddy, Louise Stanton, Shamim Kazmi-Stokes, Andrew McMillan, Paul Fields, Christopher Pocock, Graham P Collins, Richard Stephens, Francesco Cucco, Alexandra Clipson, Chulin Sha, Reuben Tooze, Matthew A Care, Gareth Griffiths, Ming-Qing Du, David R Westhead, Catherine Burton, Peter W M Johnson

**Affiliations:** a Cancer Research UK Centre, University of Southampton, Southampton, UK; b Southampton Clinical Trials Unit, University of Southampton, Southampton, UK; c Haematological Malignancy Diagnostic Service, Leeds Cancer Centre, Leeds Teaching Hospitals, Leeds, UK; d Medical Oncology, Cantonal Hospital Aarau, Switzerland; e Department of Medical Oncology, Inselspital, Bern University Hospital, University of Bern, Switzerland; f Centre for Drug Development, Cancer Research UK, London, UK; g Department of Haematology, Nottingham University Hospitals NHS Trust, Nottingham, UK; h Department of Haematology, Guy's and St Thomas' Hospitals NHS Trust, Kings Health Partners, London, UK; i Department of Haematology, East Kent Hospitals University Foundation Trust, Canterbury, UK; j Oxford Cancer and Haematology Centre, Churchill Hospital, Oxford, UK; k National Cancer Research Institute Consumer Forum, London, UK; l Department of Pathology, University of Cambridge, Cambridge, UK; m School of Molecular and Cellular Biology, Faculty of Biological Sciences, University of Leeds, Leeds, UK; n Section of Experimental Haematology, University of Leeds, Leeds, UK

## Abstract

**Background:**

Biologically distinct subtypes of diffuse large B-cell lymphoma can be identified using gene-expression analysis to determine their cell of origin, corresponding to germinal centre or activated B cell. We aimed to investigate whether adding bortezomib to standard therapy could improve outcomes in patients with these subtypes.

**Methods:**

In a randomised evaluation of molecular guided therapy for diffuse large B-cell lymphoma with bortezomib (REMoDL-B), an open-label, adaptive, randomised controlled, phase 3 superiority trial, participants were recruited from 107 cancer centres in the UK (n=94) and Switzerland (n=13). Eligible patients had previously untreated, histologically confirmed diffuse large B-cell lymphoma with sufficient diagnostic material from initial biopsies for gene-expression profiling and pathology review; were aged 18 years or older; had ECOG performance status of 2 or less; had bulky stage I or stage II–IV disease requiring full-course chemotherapy; had measurable disease; and had cardiac, lung, renal, and liver function sufficient to tolerate chemotherapy. Patients initially received one 21-day cycle of standard rituximab, cyclophosphamide, doxorubicin, vincristine, and prednisolone (R-CHOP; rituximab 375 mg/m^2^, cyclophosphamide 750 mg/m^2^, doxorubicin 50 mg/m^2^, and vincristine 1·4 mg/m^2^ [to a maximum of 2 mg total dose] intravenously on day 1 of the cycle, and prednisolone 100 mg orally once daily on days 1–5). During this time, we did gene-expression profiling using whole genome cDNA-mediated annealing, selection, extension, and ligation assay of tissue from routine diagnostic biopsy samples to determine the cell-of-origin subtype of each participant (germinal centre B cell, activated B cell, or unclassified). Patients were then centrally randomly assigned (1:1) via a web-based system, with block randomisation stratified by international prognostic index score and cell-of-origin subtype, to continue R-CHOP alone (R-CHOP group; control), or with bortezomib (RB-CHOP group; experimental; 1·3 mg/m^2^ intravenously or 1·6 mg/m^2^ subcutaneously) on days 1 and 8 for cycles two to six. If RNA extracted from the diagnostic tissues was of insufficient quality or quantity, participants were given R-CHOP as per the control group. The primary endpoint was 30-month progression-free survival, for the germinal centre and activated B-cell population. The primary analysis was on the modified intention-to-treat population of activated and germinal centre B-cell population. Safety was assessed in all participants who were given at least one dose of study drug. We report the progression-free survival and safety outcomes for patients in the follow-up phase after the required number of events occurred. This study was registered at ClinicalTrials.gov, number NCT01324596, and recruitment and treatment has completed for all participants, with long-term follow-up ongoing.

**Findings:**

Between June 2, 2011, and June 10, 2015, 1128 eligible patients were registered, of whom 918 (81%) were randomly assigned to receive treatment (n=459 to R-CHOP, n=459 to RB-CHOP), comprising 244 (26·6%) with activated B-cell disease, 475 (51·7%) with germinal centre B cell disease, and 199 (21·7%) with unclassified disease. At a median follow-up of 29·7 months (95% CI 29·0–32·0), we saw no evidence for a difference in progression-free survival in the combined germinal centre and activated B-cell population between R-CHOP and RB-CHOP (30-month progression-free survival 70·1%, 95% CI 65·0–74·7 *vs* 74·3%, 69·3–78·7; hazard ratio 0·86, 95% CI 0·65–1·13; p=0·28). The most common grade 3 or worse adverse event was haematological toxicity, reported in 178 (39·8%) of 447 patients given R-CHOP and 187 (42·1%) of 444 given RB-CHOP. However, RB-CHOP was not associated with increased haematological toxicity and 398 [87·1%] of 459 participants assigned to receive RB-CHOP completed six cycles of treatment. Grade 3 or worse neuropathy occurred in 17 (3·8%) patients given RB-CHOP versus eight (1·8%) given R-CHOP. Serious adverse events occurred in 190 (42·5%) patients given R-CHOP, including five treatment-related deaths, and 223 (50·2%) given RB-CHOP, including four treatment-related deaths.

**Interpretation:**

This is the first large-scale study in diffuse large B-cell lymphoma to use real-time molecular characterisation for prospective stratification, randomisation, and subsequent analysis of biologically distinct subgroups of patients. The addition of bortezomib did not improve progression-free survival.

**Funding:**

Janssen-Cilag, Bloodwise, and Cancer Research UK.

## Introduction

The combination of rituximab, cyclophosphamide, doxorubicin, vincristine, and prednisolone (R-CHOP) has been considered standard of care for diffuse large B-cell lymphoma for more than 15 years.^1^ In trials of R-CHOP, 5-year progression-free survival has been reported to be 70–75% and overall survival 75–80%,^2^ although unselected population-based studies show lower figures.^3^ Patients with lymphoma that does not respond to R-CHOP or that recurs have a poor prognosis, with only a third alive at 2 years after diagnosis.^4^ Various approaches have been attempted to improve outcomes for patients with diffuse large B-cell lymphoma, but none has so far increased overall survival. The recognised molecular heterogeneity of this aggressive lymphoma contributes to the complexity of this problem.^5^

Gene-expression profiling of diffuse large B-cell lymphoma has been used to define subgroups with distinct pathogenesis. Cell-of-origin classification recognises those cases with a gene expression similar to that of peripheral blood B cells undergoing in-vitro antigen activation, referred to as the activated B-cell subtype, whereas the germinal centre B-cell subtype resembles B cells in the germinal centre. Retrospective analyses suggest that the patients with the activated B-cell subtype have worse outcomes, with 40% 3-year progression-free survival after R-CHOP compared with 75% in the germinal centre B-cell group.^5^

**Research in context**

**Evidence before this study**

We searched PubMed for publications of randomised clinical trials in English between Jan 1, 1998, and Dec 1, 2010, using the terms “diffuse large B-cell lymphoma” and “cell of origin”, and studies involving “diffuse-large B-cell lymphoma” and “bortezomib”. Using gene-expression profiling to characterise patients, several retrospective studies of patients treated with rituximab, cyclophosphamide, doxorubicin, vincristine, and prednisolone (R-CHOP) or CHOP-like regimens had shown that the activated B-cell subtype was associated with inferior survival compared with the germinal centre B-cell subtype. Bortezomib had shown restricted single-agent activity in diffuse large B-cell lymphoma but had been successfully combined with standard chemotherapy regimens in a phase 2 study. In another phase 2 study, bortezomib in combination with dose-adjusted R-EPOCH (rituximab, etoposide, cyclophosphamide, doxorubicin, vincristine, and prednisolone) had resulted in longer progression-free survival in patients with the activated B-cell subtype than in those with the germinal centre B-cell subtype. Bortezomib in combination with R-CHOP had produced similar outcomes in non-germinal centre B-cell diffuse large B-cell lymphoma (ascertained by immunohistochemistry) compared with germinal centre B-cell diffuse large B-cell lymphoma in a further phase 2 study.

**Added value of this study**

To our knowledge, this study is the first to combine prospective gene-expression profiling of lymphoma with a targeted therapy to allow stratification and random assignment of patients to treatment within a phase 3 clinical trial. It is the first study to assess a novel agent in diffuse large B-cell lymphoma, prospectively powered to address subtypes defined by gene-expression profiling, and we have shown that the addition of bortezomib to R-CHOP (RB-CHOP) does not improve survival in the activated B-cell subgroup. Extensive characterisation and subgroup analyses suggest that cell-of-origin subtype and nuclear factor (NF)-κB activating mutations are not associated with improved outcomes with RB-CHOP, and that bortezomib does not act as an effective inhibitor of the NF-κB pathway in this disease. Exploratory analyses, however, suggest that different high-risk subgroups—double-expressor lymphoma and double-hit lymphoma—might benefit from the addition of bortezomib or similar agents to standard immunochemotherapy.

**Implications of all the available evidence**

The trial design provides a rational framework for future studies in diffuse large B-cell lymphoma, allowing prompt initiation of treatment while molecular characterisation is carried out. We confirm that R-CHOP is a good standard of care for most patients with diffuse large B-cell lymphoma, but raise the possibility that high-risk subgroups could benefit from the addition of a proteasome inhibitor to standard therapy, which could guide future research.

The subtypes have distinct genomic characteristics. The activated B-cell subtype shows a higher prevalence of mutations in genes involved in B-cell receptor signalling and regulators of nuclear factor (NF)-κB (*MYD88, CD79B, TNFAIP3, CARD11, TRAF2, TRAF5, MAP3K7*, and *TNFRSF11A*) than the germinal centre B-cell subtype. Constitutive NF-κB activation downstream of the B-cell receptor is a feature of the activated B-cell subtype of diffuse large B-cell lymphoma. Genomic, pharmacological, and RNA interference screens have shown selective oncogenic addiction of the activated B-cell subtype to activation of this protein complex.^6^ Bortezomib is a proteasome inhibitor and can suppress NF-κB activity by preventing proteosomal degradation of the inhibitor IκBα, thereby keeping NF-κB inactive and unable to translocate to the nucleus to mediate transcription. Preliminary clinical studies^7^ suggested that bortezomib had selective efficacy in diffuse large B-cell lymphoma subtypes. When combined with infusional chemotherapy, bortezomib appeared to have preferential activity in relapsed or refractory activated B-cell diffuse large B-cell lymphoma, with a higher response rate and median overall survival than that achieved with infusional chemotherapy alone.^7^

The randomised evaluation of molecular guided therapy for diffuse large B-cell lymphoma with bortezomib (REMoDL-B) study aimed to investigate the clinical efficacy of R-CHOP in addition to bortezomib in patients with diffuse large B-cell lymphoma. To determine whether the cell-of-origin subtypes respond differently to the combination of bortezomib with R-CHOP, we used a study design that incorporated prospective randomisation stratified by whole transcriptome gene-expression profiling. We also incorporated molecular characterisation into our analysis to assess recognised subgroups distinct from the cell-of-origin subgroups: double-hit (rearrangements of *MYC* and *BCL2* or *BCL6*, or both) and double-expressor lymphomas (high expression of MYC and BCL2 proteins).

As clinical studies move towards increased application of targeted drugs against molecular phenotype, the feasibility of determining a molecular phenotype in real-time was an important objective of the study.

## Methods

### Study design and participants

In this multicentre, open-label, randomised, phase 3, superiority trial, we compared R-CHOP with R-CHOP plus bortezomib (RB-CHOP) in patients with newly diagnosed diffuse large B-cell lymphoma. In a collaboration between the UK National Cancer Research Institute group and the Schweiz Arbeitsgemeinschaft für Klinische Krebsforschung in Switzerland, patients were recruited from 107 cancer centres in the UK (n=94) and Switzerland (n=13). Patients were eligible for inclusion in the study if they had de novo diffuse large B-cell lymphoma confirmed by an expert haematopathologist (CB) with sufficient diagnostic material from previous biopsies for gene-expression profiling and central pathological review; were aged 18 years or older; had Eastern Cooperative Oncology Group (ECOG) performance status of 2 or less; had bulky stage I or stage II–IV disease requiring full-course chemotherapy; had measurable disease; and had cardiac, lung, renal, and liver function sufficient to tolerate chemotherapy. Patients with a previous history of indolent lymphoma were excluded, but patients with previously undiagnosed concurrent low-grade infiltration in bone marrow or lymph nodes were eligible. Patients with primary mediastinal lymphoma; clinical CNS involvement; positive serology for HIV, hepatitis B virus, or hepatitis C virus; active malignancy in the preceding 5 years; or other conditions precluding administration of study treatment were ineligible. Pregnant women were also excluded. Full inclusion and exclusion criteria are in the appendix (pp 43–44).

The institutional review board at each study site approved the protocol. The full study protocol is available in the appendix (pp 15–90). Independent trial oversight was maintained by a trial steering committee and the data monitoring and ethics committee. The study was carried out according to the principles of the Declaration of Helsinki, Principles of the International Conference on Harmonisation of Technical Requirements for Registration of Pharmaceuticals for Human Use Good Clinical Practice, and in accordance with UK and Swiss regulatory requirements. Written informed consent was obtained from all patients.

### Randomisation and masking

Participants were centrally randomly assigned (1:1) with block randomisation of varying block size by TENALEA, a web-based system, to receive either R-CHOP (control) or RB-CHOP (experimental). Randomisation stratification factors included cell-of-origin subtype and international prognostic index (IPI) score. For the purposes of stratification, participants were grouped by their IPI scores as: low (0–1), intermediate (2–3), and high (4–5), and those with an unclassified cell-of-origin subtype were included. In cases of failed RNA extraction or insufficient RNA yield, participants were not randomly assigned but were given conventional R-CHOP treatment and followed up with the same assessments as participants in the control group, but analysed as a distinct group. Participants, investigators, and treating clinicians were unmasked to the treatment allocation; however, local investigators were not informed which molecular subgroup the participants were in.

### Procedures

Participants underwent routine staging investigations, including CT scans and bone marrow biopsy, with examination of cerebrospinal fluid as clinically indicated. Tumour material was sent to the central laboratory (Haematological Malignancy Diagnostic Service, Leeds Cancer Cancer Centre, Leeds Teaching Hospitals, Leeds, UK) for gene-expression profiling and somatic mutation assessment.

For cycle one, all participants received the R-CHOP regimen on a 21-day schedule. The regimen comprised rituximab 375 mg/m^2^ intravenously, cyclophosphamide 750 mg/m^2^ intravenously, doxorubicin 50 mg/m^2^ intravenously, and vincristine 1·4 mg/m^2^ (maximum total dose 2 mg) intravenously on day 1 of the cycle, and prednisolone 100 mg orally once daily on days 1–5. From cycle 2 onwards, participants were randomly assigned to their treatment groups, either to receive five further cycles of R-CHOP in the control group, or five cycles of R-CHOP plus bortezomib (RB-CHOP) on days 1 and 8 (1·3 mg/m^2^ intravenously or 1·6 mg/m^2^ subcutaneously) in the experimental group. Further cycles were given when neutrophils had recovered to 1·0 × 10^9^ per L and platelets to 100 × 10^9^ per L; dose reductions of bortezomib in response to neurotoxicity were closely specified according to severity of this toxicity (appendix pp 48–53).

On Feb 28, 2014, we changed the route of bortezomib from intravenous to subcutaneous administration and updated the protocol after publication of data^7^ that suggested subcutaneous administration was associated with decreased toxicity and similar efficacy at a lower dose, and greater acceptability to patients, as compared with intravenous administration.^8^ Patients who were already being given intraveous bortezomib continued on this formulation. Allopurinol, granulocyte colony-stimulating factor, and anti-emetic therapy were given according to local policy. Intrathecal prophylaxis with methotrexate was recommended for patients at high risk of CNS relapse for three to six cycles and could be given at any time at investigators' discretion at each study site. Radiotherapy to initial bulky disease, extranodal sites, or residual masses was done according to routine practice in the participating centres. Cross-sectional imaging was repeated 1 month after administration of the final dose of chemotherapy to assess disease response using the International Working Group Response Criteria for non-Hodgkin lymphoma^9^ and repeated at 12 months.

Participants were assessed clinically at the beginning of each treatment cycle and after treatment completion every 3 months for 1 year and thereafter every 6 months until 5 years' total follow-up. At each assessment, medical history was recorded including adverse events, and patients underwent a physical examination, ECOG performance status assessment, and routine laboratory tests. Progressions were recorded after clinical assessment and imaging, determined by local investigators, according to standard criteria,^9^ and at progression trial treatment was discontinued and patients were followed up until data cut off for survival.

Histological haematoxylin and eosin sections from formalin-fixed paraffin-embedded (FFPE) samples taken at diagnosis were reviewed in the central laboratory as a quality check. Macrodissection of tumours was done by scraping the area of interest from unstained sections on plain microscope slides. RNA was extracted using an Ambion RecoverAll Total Nucleic Acid Isolation Kit for FFPE (ThermoFisher, Waltham, MA, USA) according to the manufacturer's protocol, with the exception that two washes in xylene and alcohol were used to remove wax, with extended digestion in proteinase K overnight.

During cycle 1 of R-CHOP, gene-expression profiling was done (by SB) using Illumina whole genome cDNA-mediated annealing, selection, extension, and ligation (DASL) assay (Illumina, San Diego, CA, USA). Patient samples were classified as activated B-cell, germinal centre B-cell, unclassified, or fail (ie, insufficient quality or quantity of DNA or failure of DASL array) by use of the DASL automated classifier as previously described,^10^ with a quality control of a score over 1 to define technical failure. The confidence of each sample being one of the three classes was recorded and the final classification was defined as that with the highest confidence score.

When possible, we used tissue from the biopsy sample to construct tissue micorarrays for immunohistochemistry, fluorescence in-situ hybridisation, and DNA extraction. Specifically, we did immunohistochemistry for MYC and BCL2 protein (dual) expression using these tissue micorarrays and Abcam rabbit monoclonal antibodies (Abcam, Cambridge, UK; clone Y69), with a cutoff of 40% or more, and Dako anti-BCL-2 monoclonal antibodies (Agilent, Santa Clara, USA; clone 124) with a cutoff of 50% or more, scored by two independent assessors according to recognised criteria. In the event of a disagreement about the score, a third assessor would arbitrate. For samples that could not be defined as positive or negative for expression of one of the proteins, these samples were defined as borderline. Using these criteria, samples with high or average MYC expresion and high or average BCL2 expression were used to define cutoff values for associated mRNA concentrations. These cutoff values were used to identify categories of *MYC* and *BCL2* gene expression: high or average.

DNA was extracted from tumour cells enriched by microdissection on FFPE tissue sections and its quality was assessed by PCR of variously sized genomic fragments. A panel of 70 genes that are recurrently mutated in aggressive B-cell lymphomas were investigated for mutation by targeted sequencing using HaloPlexHS target enrichment (Agilent Technologies, Santa Clara, CA, USA) and Illumina HiSeq sequencing, as described previously.^11^ This process was carried out for participants who had DNA available of adequate quantity and quality. Duplicate experiments were done for samples of lower quality, including all those with quality control PCR showing amplification of 300 bp or fewer genomic fragments, and only those mutations that were reproducible in both experiments were reported. Samples of better quality were investigated in a single replicate. Variant calling, single nucleotide polymorphisms, and background noise filtering were done as previously described.^11^ In a further 22 samples, mutations in 20 genes (included in the above panel of 70 genes) were analysed in duplicate using Fluidigm multiplex PCR (Fluidigm, South San Francisco, CA, USA) and Illumina MiSeq sequencing, as described previously, because of evolution of molecular diagnostics during the study period.^11^

Variants detected by use of these targeted sequencing methods were further assessed by use of functional prediction tools. These tools comprised SIFT, Polyphen2 HDIV, Polyphen2 HVAR, LRT, MutationTaster, MutationAssessor, FATHMM, SVM score, and LR score, which predict whether or not a variant has an effect on the protein function, and those variants predicted to be benign by seven or more of nine programs, not in the Catalogue Of Somatic Mutations In Cancer database, were excluded. The resulting variants were further scrutinised by reviewing the binary alignment map (.bam) file to eliminate any potential PCR or sequence artefacts. As part of a post-hoc analysis, samples were tested for the possible presence of primary mediastinal lymphoma using a Bayesian predictor described by the Lymphoma Molecular Profiling Project to ensure that no molecular cases of primary mediastinal lymphoma had been included.^12^

Safety was assessed by the recording and grading of adverse events according to the Common Terminology Criteria for Adverse Event Reporting Version 4.0 at each study visit, or between visits if notified. The assessment of causality was related to the study drugs by the local investigator. Serious or severe adverse events, including mention of suspected unexpected serious adverse reactions were defined as per the medicines for human use (clinical trials) regulations 2004.

### Outcomes

The primary endpoint was 30-month progression-free survival in the germinal centre and activated B-cell popualtion, defined as time from registration to the date of progression or death from any cause. Disease progression was determined using the International Working Group Response Criteria for non-Hodgkin lymphoma.^9^ Participants free from progression or death were censored at the date of their last visit. Secondary outcomes were 30-month progression-free survival by cell-of-origin subgroup**;** the time-to-event variables of overall survival, event-free survival, disease-free survival, and time to progression; response duration; complete and overall proportion of patients who achieved a response; assessment of toxicity; quality of life; assessment of peripheral neuropathy up to 30 days after last treatment; and safety. The proportion of patients who achieved a complete and overall response, duration of response, event-free survival, disease-free survival, time-to-progression, and quality-of-life assessments will be reported elsewhere. Exploratory analyses were planned for potential prognostic factors that emerged during the course of the trial, particularly new genomic risk categories.

### Statistical analysis

We used an adaptive design based on a two-stage approach, with two interim analyses to explore the safety and efficacy in the germinal centre B-cell group treated with RB-CHOP after a defined number of events. The first interim analysis was to take place once 55 patients in the germinal centre B-cell group had been randomly assigned to receive RB-CHOP. If progression-free survival at 12 months was assessed to be below 70% in this subgroup, the trial would stop recruiting into the germinal centre B-cell group. The second interim analysis was to take place when 73 patients in the germinal centre B-cell group had been randomly assigned to receive RB-CHOP and followed up for 1 year. If the progression-free survival at 12 months was assessed to be below 85% in this subgroup, the trial would stop recruiting into the germinal centre B-cell group.

The trial was powered to detect an improvement in progression-free survival at 30 months of 10% in the combined activated B-cell and germinal centre B-cell groups, from 75% in the R-CHOP group to 85% in the RB-CHOP group (corresponding to a hazard ratio [HR] of 0·56), on the basis of a log-rank test with a significance level of 5% (two-sided) and 90% power, requiring a total of 129 events. The intention-to-treat (ITT) population comprised all patients for whom gene-expression profiling was attempted (classified as activated B-cell, germinal centre B-cell, or unclassified subgroups, or for whom gene-expression profiling failed). The safety population was formed of all patients in the ITT population who received at least one dose of any study drug.

We assessed the primary outcome of 30-month progression-free survival in a modified ITT (mITT) population comprising the activated and germinal centre B-cell subgroups who were randomly assigned to receive treatment, using a Cox proportional hazards model, adjusted for cell-of-origin subtype and IPI score.

Secondary outcome analyses included repeating the primary outcome analysis in the activated B-cell ITT population alone, the germinal centre B-cell ITT population, and the unclassified ITT population, adjusting for IPI score only. We produced Kaplan-Meier curves for time-to-event data and we described follow-up maturity by the reverse Kaplan-Meier method. We used summary statistics to describe baseline characteristics for participants in the R-CHOP group, RB-CHOP group, and patients for whom gene-expression profiling had failed in the ITT population, and by cell-of-origin subgroups in the ITT population, with formal comparisons between cell-of-origin subgroups using Pearson χ^2^ tests. Toxicity information was summarised by treatment group, and we did post-hoc analyses to compare toxicity information by treatment using Pearson χ^2^ tests for the safety population. Further post-hoc analyses included repeating the primary outcome analysis and adjusting for time from diagnosis to the start of treatment to ascertain whether or not the interval from diagnosis to treatment affected the progression-free survival outcome. We also did post-hoc analyses to assess progression-free survival and overall survival by treatment group in the mITT population in the IPI low, intermediate, and high score groups, assessed using a Cox proportional hazards model, adjusted for cell-of-origin subtype, and also repeated the primary outcome analysis excluding patients who had a dose reduction in any treatment drug. We made no adjustment for multiple comparisons.

Post-hoc analyses to assess progression-free survival also included: comparing double-hit lymphomas to non-rearranged cases, separated by treatment group; comparing double-expressor lymphomas to all other cases, separated by treatment group; and comparing by treatment groups in subgroups with mutations in components of the NF-κB pathway.

We used Stata statistical software (version 15.1) for all analyses. This trial is registered with ClinicalTrials.gov, number NCT01324596.

### Role of the funding source

The funders had no role in study design, data collection, data analysis, data interpretation, or writing of the report. The corresponding author had full access to all the data in the study and had final responsibility for the decision to submit for publication.

## Results

**Figure 1:**
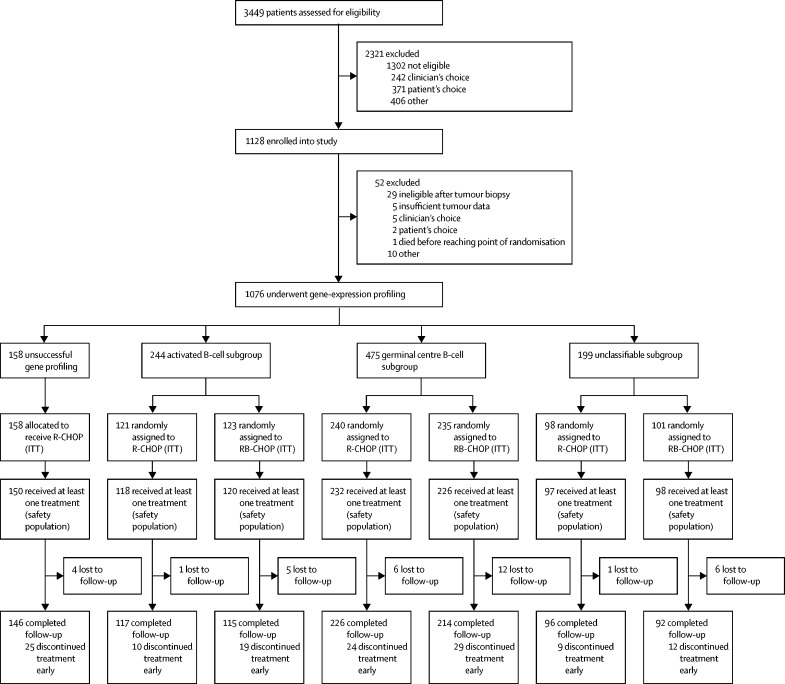
Study profile ITT=intention-to-treat. R-CHOP=rituximab, cyclophosphamide, doxorubicin, vincristine, and prednisolone. RB-CHOP=rituximab, bortezomib, cyclophosphamide, doxorubicin, vincristine, and prednisolone.

Between June 2, 2011, and June 10, 2015, of 3449 patients assessed for eligibility, 1128 (32·7%) participants were registered to the study (figure 1). Of the registered participants, a further 52 who received one cycle of R-CHOP were excluded for reasons including ineligibility after tumour biopsy (n=29) and insufficient tumour data (n=5). 158 (14·7%) of 1076 remaining participants had inadequate sample material for gene-expression profiling and so were excluded from subsequent random assignment to treatment, and instead given R-CHOP as per the control group. 918 (85·3%) of 1076 participants were stratified by cell-of-origin subtype and IPI and randomly assigned to receive R-CHOP or RB-CHOP (figure 1). Overall, 244 (26·6%) patients had activated B-cell disease, 475 (51·7%) had germinal centre B-cell disease, and 199 (21·7%) had unclassified disease. The planned interim analyses and safety assessments by the data monitoring and ethics committee in the germinal centre B-cell group showed the 1-year progression-free survival to be 70% or above in the germinal centre B-cell subgroup at the first interim analysis and the 1-year progression-free survival to be 85% or above in the germinal centre B-cell subgroup at the second interim analysis. Hence, the trial continued to recruit to all groups.

Baseline characteristics were similar between the control group, experimental group, and non-randomised participants (table 1). The median turnaround time from tumour samples reaching the diagnostic laboratory to a cell-of-origin result being available was 8 days (IQR 6–12). 14·7% (158 of 1076) of samples failed, mainly because of insufficient tissue remaining in the block. For the samples from which sufficient material was extracted, only 1% (11 of 1076) failed for technical reasons.

**Table 1:** Baseline characteristics of the participants by treatment group

		**R-CHOP (n=459)**	**RB-CHOP (n=459)**	**Not randomised (n=158)**
Age, years		65 (24–86)	63 (20–84)	65 (24–85)
ECOG performance status		n=443	n=443	n=154
	0	250 (56·4%)	222 (50·1%)	73 (47·4%)
	1	142 (32·1%)	168 (37·9%)	57 (37·0%)
	2	51 (11·5%)	53 (12·0%)	24 (15·6%)
Bone marrow involvement		n=448	n=448	n=157
	Yes	78 (17·4%)	63 (14·1%)	34 (21·7%)
	No	370 (82·6%)	385 (85·9%)	123 (78·3%)
Serum LDH level		n=377	n=368	n=108
	>ULN	224 (59·4%)	227 (61·7%)	77 (71·3%)
	≤ULN	153 (40·6%)	141 (38·3%)	31 (28·7%)
IPI score				
	Low (0–1)	123 (26·8%)	120 (26·1%)	33 (20·9%)
	Low intermediate (2)	111 (24·2%)	123 (26·8%)	45 (28·5%)
	High intermediate (3)	145 (31·6%)	133 (29·0%)	51 (32·3%)
	High (4–5)	80 (17·4%)	83 (18·1%)	29 (18·4%)
Stage of disease		n=457	n=457	n=157
	I	12 (2·6%)	14 (3·1%)	5 (3·2%)
	II	131 (28·7%)	126 (27·6%)	37 (23·6%)
	III	128 (28·0%)	154 (33·7%)	48 (30·6%)
	IV	186 (40·7%)	163 (35·7%)	67 (42·7%)
Bulk >10 cm		n=456	n=450	n=149
	Yes	122 (26·8%)	141 (31·3%)	66 (42·6%)
Molecular phenotype				
	Activated B cell	121 (26·4%)	123 (26·8%)	..
	Germinal centre B cell	240 (52·3%)	235 (51·2%)	..
	Unclassified	98 (21·4%)	101 (22·0%)	..

Data are median (range) or n (%). R-CHOP=rituximab, cyclophosphamide, doxorubicin, vincristine, and prednisolone. RB-CHOP=rituximab, bortezomib, cyclophosphamide, doxorubicin, vincristine, and prednisolone. ECOG=Eastern Cooperative Oncology Group. LDH=lactate dehydrogenase. ULN=upper limit of normal. IPI=international prognostic index.

We observed clinical differences between the molecular subgroups (table 2). Median age was higher in the activated B-cell subgroup (p=0·0045) and bulky disease occurred more often in the germinal centre B-cell subgroup (p<0·0001). Patients with bone marrow involvement were over-represented in the unclassified subgroup. No significant difference was seen between the activated and germinal centre B-cell subgroups in the distribution of IPI risk group, serum lactate dehydrogenase (LDH) concentration above the upper limit of normal, conventional stage of disease, overall prevalence of extranodal disease, or ECOG performance status (table 2; appendix p 6). Post-hoc testing for the possible presence of primary mediastinal lymphoma identified 19 participants who fulfilled the criteria,^12^ of whom 13 (68%) had mediastinal disease. Of these participants, 14 (74%) had been allocated to the germinal centre B-cell group and five (26%) to the unclassified subgroup.

**Table 2:** Clinical characteristics of participants in intention-to-treat population by cell-of-origin subtypes

		**Activated B-cell subgroup (n=244)**	**Germinal centre B-cell subgroup (n=475)**	**Unclassified subgroup (n=199)**	**p value (activated *vs* germinal centre B-cell groups)**
Age, years		67 (22–86)	63 (20–82)	63 (20–84)	0·0045
ECOG performance status		n=233	n=459	n=194	0·83
	0	121 (51·9%)	247 (53·8%)	104 (53·6%)	..
	1	84 (36·1%)	158 (34·4%)	68 (35·1%)	..
	2	28 (12·0%)	54 (11·8%)	22 (11·3%)	..
Bone marrow involvement		33/240 (13·8%)	66/465 (14·2%)	42/191 (22·0%)	0·017
Serum LDH level >ULN		115/189 (60·8%)	231/386 (59·8%)	105/170 (61·8%)	0·19
IPI score		n=244	n=475	n=199	0·822
	Low (0–1)	66 (27·0%)	127 (26·7%)	50 (25·1%)	..
	Low intermediate (2)	70 (28·7%)	117 (24·6%)	47 (23·6%)	..
	High intermediate (3)	69 (28·3%)	144 (30·3%)	65 (32·7%)	..
	High (4–5)	39 (16·0%)	87 (18·3%)	37 (18·6%)	..
Stage of disease		n=244	n=471	n=199	0·74
	I	8 (3·3%)	12 (2·5%)	6 (3·0%)	..
	II	76 (31·1%)	134 (28·5%)	47 (23·6%)	..
	III	73 (29·9%)	148 (31·4%)	61 (30·7%)	..
	IV	87 (35·7%)	177 (37·6%)	85 (42·7%)	..
Bulk >10 cm		50/241 (20·7%)	158/467 (33·8%)	55/198 (27·8%)	<0·0001

Data are median (range), n (%), or n/N (%). ECOG=European Cooperative Oncology Group. LDH=lactate dehydrogenase. ULN=upper limit of normal. IPI=international prognostic index.

The primary efficacy outcome was analysed when the combined activated and germinal centre B-cell mITT population had been followed up for a median of 30 months, as stipulated in the protocol (median follow-up 29·7 months [95% CI 29·0–32·0]; median follow-up of survivors: 29·4 months [28·6–31·1]). In the combined activated and germinal centre B-cell mITT population, 198 progression-free survival events (ie, progression or death) occurred (in 107 [29·6%] of 361 participants in the R-CHOP group and in 91 [25·4%] of 358 in the RB-CHOP group), giving a Kaplan-Meier estimate of 30-month progression-free survival of 70·1% (95% CI 65·0–74·7) for the R-CHOP group and 74·3% (69·3–78·7) for the RB-CHOP group. We saw no difference in progression-free survival in the combined activated and germinal centre B-cell populations between the R-CHOP and RB-CHOP groups (HR 0·86, 95% CI, 95% CI 0·65–1·13; p=0·28; adjusted HR 0·84, 95% CI 0·64–1·11; p=0·23). 116 overall survival events (ie, deaths) occurred (62 in the R-CHOP group and 54 in the RB-CHOP group). The Kaplan-Meier estimate for 30-month overall survival was 82·7% (95% CI 78·2–86·3) for the R-CHOP group and 83·6% (79·0–87·3) for the RB-CHOP group (HR 0·89, 95% CI 0·62–1·28; p=0·52; adjusted HR 0·85, 0·59–1·23; p=0·40).

**Figure 2:**
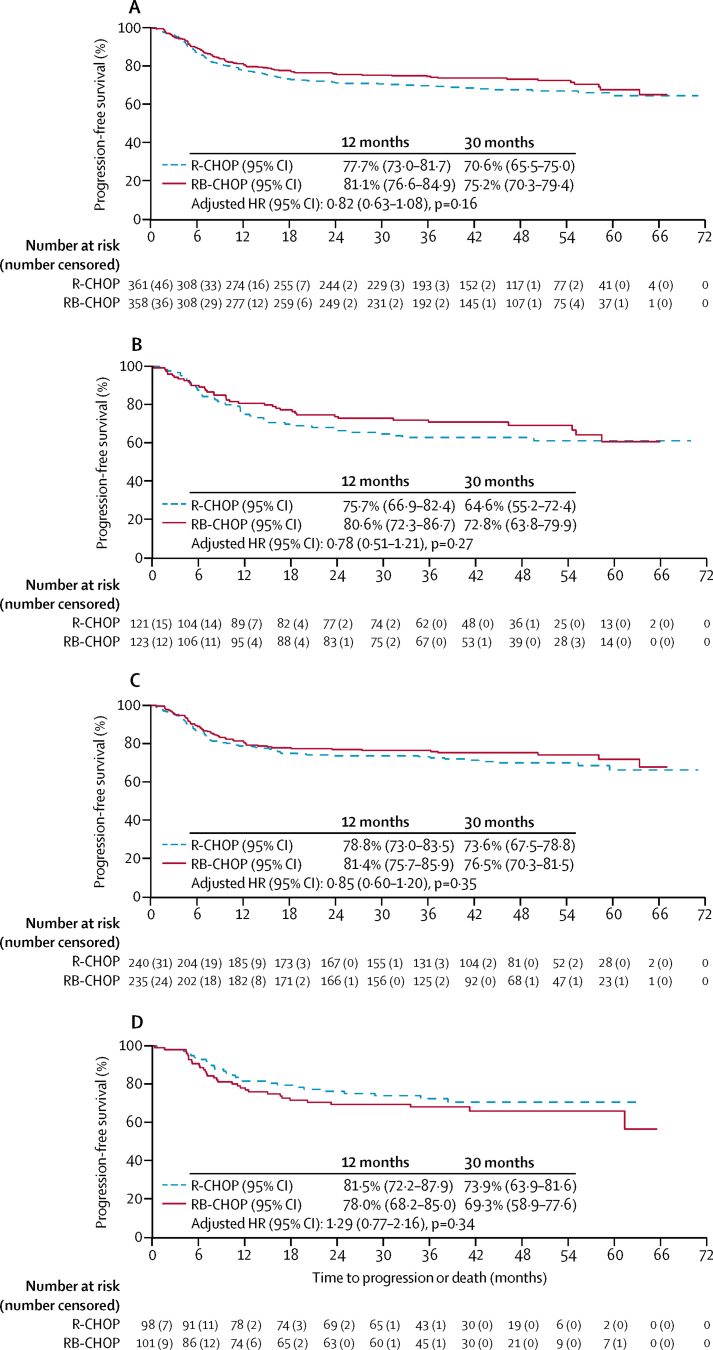
Progression-free survival in the m ITT population (A), activated B-cell subgroup (B), germinal centre B-cell subgroup (C), and unclassified group (D), by treatment Data are for the mITT population, which comprises germinal centre and activated B-cell ITT participants (n=719); activated B-cell subgroup (n=244); germinal centre B-cell subgroup (n=475); and unclassified subgroup (n=199); with estimated proportions of participants achieving progression-free survival at 12 months and 30 months. HR=hazard ratio. ITT=intention-to-treat. mITT=modified ITT. R-CHOP=rituximab, cyclophosphamide, doxorubicin, vincristine, and prednisolone. RB-CHOP=rituximab, bortezomib, cyclophosphamide, doxorubicin, vincristine, and prednisolone.

Although no adjustment for multiple testing was done, efficacy analyses were repeated after additional follow-up data were collected. After a median follow-up of survivors in the mITT population of 42·3 months (95% CI 40·9–45·6), 211 progression-free survival events were observed (115 [31·9%] of 361 in the R-CHOP group and 96 [26·8%] of 358 in the RB-CHOP group), and 133 overall survival events were observed (72 [19·9%] of 361 participants died in the R-CHOP group and 61 [17·0%] of 358 died in the RB-CHOP group). After this additional follow-up, the Kaplan-Meier estimate for 30-month progression-free survival in the combined activated and germinal centre B-cell mITT population was 70·6% (95% CI 65·5–75·0) for the R-CHOP group and 75·2% (70·3–79·4) for the RB-CHOP group. We saw no evidence of difference in 30-month progression-free survival in the mITT population between the R-CHOP and RB-CHOP groups (adjusted HR 0·82, 95% CI 0·63–1·08; p=0·16; figure 2A).

Secondary analysis of subtypes by cell of origin showed that bortezomib did not significantly affect progression-free survival in either the activated B-cell (adjusted HR 0·78, 95% CI 0·51–1·21; p=0·27), germinal centre B-cell (0·85, 0·60–1·20; p=0·35), or unclassifiable participants (1·29, 95% CI 0·77–2·16; p=0·34; figure 2). We saw no difference in overall survival by treatment group in the mITT population (72 deaths in the R-CHOP group and 61 in the RB-CHOP group; adjusted HR 0·82, 95% CI 0·59–1·16; p=0·27), and the Kaplan-Meier estimate of overall survival at 30 months was 81·6% (95% CI 77·1–85·3) in the R-CHOP group versus 83·1% (78·7–86·7) in the RB-CHOP group (appendix p 3).

The addition of bortezomib to R-CHOP was well tolerated (table 3). The most common grade 3 or worse adverse event was haematological toxicity, in 178 (39·8%) of 447 patients given R-CHOP and 187 (42·1%) of 444 given RB-CHOP. However, in a post-hoc analysis of adverse events between groups, we saw no significant increase in the proportion of patients who had grade 3 or worse neutropenia, febrile neutropenia, thrombocytopenia, or anaemia. Neuropathy of any grade was more frequent in participants given RB-CHOP than among those given R-CHOP (252 (56·8%) RB-CHOP *vs* 186 (41·6%) given R-CHOP; p<0·0001; appendix pp 8–11) but there was no significant difference in the event rate of neuropathy of grade 3 or higher (17 (3·8%) RB-CHOP *vs* eight (1·8%) R-CHOP; p=0·070). 190 (42·5%) participants given R-CHOP versus 223 (50·2%) given RB-CHOP had serious adverse events. Nine suspected unexpected serious adverse reactions were reported: four reactions in four participants in the R-CHOP group (haemophagocytic syndrome, leukaemia secondary to chemotherapy, neutropenic sepsis, and fracture), and five reactions in four participants in the RB-CHOP group (jejunal stricture with small bowel obstruction, bowel perforation, sepsis, and one patient had both renal failure and tumour lysis syndrome). In the safety population, 73 (16·3%) of 447 participants in the R-CHOP group and 68 (15·3%) of 444 in the RB-CHOP group died, with most deaths due to progressive lymphoma (50 [68·5%] of 73 in the R-CHOP group, and 54 [79·4%] of 68 in the RB-CHOP group); nine treatment-related deaths were reported (five [6·8%] of 73 in the R-CHOP group, four [5·9%] of 68 in the RB-CHOP group; appendix p 13).

**Table 3:** Adverse events in the safety population

	**R-CHOP (n=447)**				**RB-CHOP (n=444)**			
	Grade 1–2	Grade 3	Grade 4	Grade 5	Grade 1–2	Grade 3	Grade 4	Grade 5
Any adverse event	414 (92·6%)	226 (50·6%)	107 (23·9%)	6 (1·3%)	415 (93·5%)	253 (57·0%)	105 (23·6%)	4 (0·9%)
Haematological	115 (25·7%)	128 (28·6%)	96 (21·5%)	1 (0·2%)	118 (26·6%)	153 (34·5%)	89 (20·0%)	1 (0·2%)
Neutropenia	51 (11·4%)	107 (23·9%)	92 (20·6%)	1 (0·2%)	62 (14·0%)	137 (30·9%)	81 (18·2%)	1 (0·2%)
Thrombocytopenia	22 (4·9%)	5 (1·1%)	2 (0·4%)	0	36 (8·1%)	7 (1·6%)	7 (1·6%)	0
Anaemia	73 (16·3%)	19 (4·3%)	0	0	82 (18·5%)	14 (3·2%)	0	0
Neuropathy	183 (40·9%)	8 (1·8%)	0	0	249 (56·1%)	17 (3·8%)	0	0
Nausea or vomiting	160 (35·8%)	7 (1·6%)	0	0	194 (43·7%)	15 (3·4%)	1 (0·2%)	0
Febrile neutropenia	8 (1·8%)	49 (11·0%)	14 (3·1%)	0	7 (1·6%)	51 (11·5%)	9 (2·0%)	0
Neutropenic sepsis	3 (0·7%)	9 (2·0%)	23 (5·1%)	1 (0·2%)	3 (0·7%)	19 (4·3%)	11 (2·5%)	1 (0·2%)
Febrile neutropenia or neutropenic sepsis	11 (2·5%)	55 (12·3%)	33 (7·4%)	1 (0·2%)	10 (2·3%)	67 (15·1%)	20 (4·5%)	1 (0·2%)
Abdominal pain	61 (13·6%)	12 (2·7%)	1 (0·2%)	0	64 (14·4%)	9 (2·0%)	0	0
Alopecia	114 (25·5%)	9 (2·0%)	0	0	106 (23·9%)	6 (1·4%)	0	0
Constipation	165 (36·9%)	1 (0·2%)	0	0	180 (40·5%)	5 (1·1%)	0	0
Cough	53 (11·9%)	0	0	0	63 (14·2%)	1 (0·2%)	0	0
Diarrhoea	95 (21·3%)	10 (2·2%)	0	0	133 (30·0%)	24 (5·4%)	0	0
Dyspnoea	56 (12·5%)	4 (0·9%)	0	0	59 (13·3%)	4 (0·9%)	0	0
Fatigue	201 (45·0%)	10 (2·2%)	0	0	191 (43·0%)	8 (1·8%)	0	0
Fever	64 (14·3%)	17 (3·8%)	1 (0·2%)	0	87 (19·6%)	14 (3·2%)	1 (0·2%)	0
Mucositis	73 (16·3%)	2 (0·4%)	0	0	62 (14·0%)	6 (1·4%)	0	0
Nausea	141 (31·5%)	3 (0·7%)	0	0	165 (37·2%)	8 (1·8%)	1 (0·2%)	0
Pain	56 (12·5%)	5 (1·1%)	0	0	69 (15·5%)	6 (1·4%)	0	0
Peripheral sensory neuropathy	129 (28·9%)	3 (0·7%)	0	0	182 (41·0%)	8 (1·8%)	0	0
Sepsis	2 (0·4%)	2 (0·4%)	12 (2·7%)	0	0	2 (0·5%)	15 (3·4%)	0
Vomiting	63 (14·1%)	6 (1·3%)	0	0	109 (24·5%)	11 (2·5%)	0	0

Data are for adverse events for which grade 1 or 2 events were reported in 10% or more of patients, adverse events for which grade 3, 4, or 5 events were reported in 2% or more of patients, and any other haematological or neutropenia-related adverse events reported. R-CHOP=rituximab, cyclophosphamide, doxorubicin, vincristine, and prednisolone. RB-CHOP=rituximab, bortezomib, cyclophosphamide, doxorubicin, vincristine, and prednisolone.

In the ITT population, fewer participants in the R-CHOP group had dose reductions in any drug than did those in the RB-CHOP group (158 [34·5%] of 459 *vs* 196 [42·9%] of 459, not including the non-randomsied participants; appendix p 12). Fewer participants discontinued from trial treatments in the R-CHOP group than in the RB-CHOP group (43 [9·4%] *vs* 60 [13·1%]; appendix p 11). However, median relative dose intensity for participants in the control and experimental groups was similar for drugs comprising R-CHOP and a high proportion of participants in both groups successfully completed six cycles of treatment: 418 (91·3%) of 459 in the R-CHOP group and 398 (87·1%) of 459 in the RB-CHOP group (appendix p 12).

The median time from diagnosis to first treatment was similar between treatment groups (R-CHOP 17 days [IQR 10–29]; RB-CHOP 20 days [10–32]). Post-hoc analyses, repeating the primary analysis and adjusting for the time from diagnosis to first treatment interval also showed no difference in 30-month progression-free survival in the mITT population between the R-CHOP and RB-CHOP groups (70·6%, 95% CI 65·5–75·0 for R-CHOP, and 75·2%, 70·3–79·4 for RB-CHOP; adjusted HR 0·83, 95% CI 0·56–1·24; p=0·36). Similarly, post-hoc analyses excluding patients who had a dose reduction in any treatment drug showed no difference in 30-month progression-free survival in the mITT population between the R-CHOP and RB-CHOP groups (68·9%, 95% CI 62·4–74·5 for R-CHOP and 74·3%, 65·8–81·0 for RB-CHOP; adjusted HR 0·80, 95% CI 0·54–1·19; p=0·27). Post-hoc analysis of progression-free survival and overall survival by IPI score are shown in the appendix (p 4).

The panel of genomic mutations confirmed the known association of different somatic changes with cell-of-origin subtypes, with a bias towards alterations in epigenetic modifier genes in the germinal centre B-cell subgroup and genes of the B-cell receptor signalling pathway in the activated B-cell subgroup (appendix pp 2, 6, 7). Mutations in *EZH2* were seen in 25·0% (53 of 212) of germinal centre B-cell biopsy samples but only 4·2% (five of 118) of activated B-cell samples, and conversely mutations in *MYD88* were found in 9·0% (19 of 212) of germinal centre B-cell and 44·9% (52 or 118) of activated B-cell samples tested (appendix pp 1, 6, 7). NF-κB target genes were expressed at higher levels in the activated B-cell subgroup compared with the germinal centre B-cell group (appendix p 2).

417 biopsy samples (118 from the activated B-cell subgroup, 212 from the germinal centre B-cell subgroup, and 87 from the unclassified subgroup) were suitable for construction of tissue microarrays. We analysed recognised prognostic subgroups in the ITT population (appendix p 13). Karyotypic double-hit lymphomas were rare in the activated B-cell population and were significantly associated with the germinal centre B-cell subtype (one [0·4%] of 244 *vs* 32 [6·7%] of 475; p<0·0001). Conversely, the activated B-cell subtype was associated with higher concomitant expression of MYC and BCL-2 proteins (ie, double-expressor lymphomas) than the germinal centre B-cell subtype was by immunohistochemistry analysis (excluding double-hit lymphomas; 56 [54·9%] *vs* 45 [26%]; p<0·0001) and mRNA (109 [44·7%] *vs* 87 [18·3%]; p<0·0001; appendix p 13).

**Figure 3:**
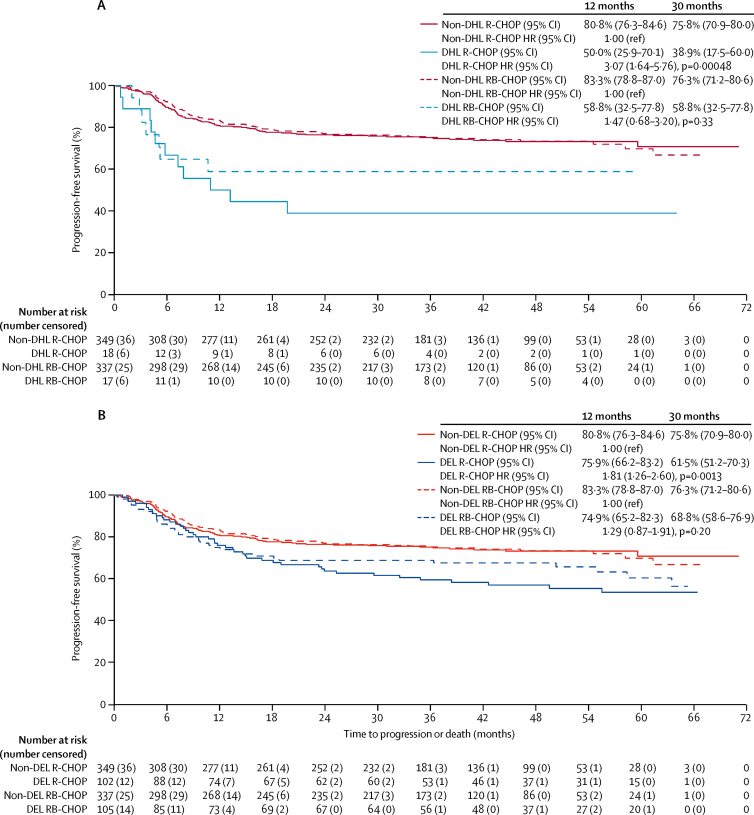
Progression-free survival comparing double-hit lymphomas to non-rearranged cases (A) and double-expressor (high *MYC* and high *BCL-2* mRNA) lymphomas to all other cases (B), by treatment group Data are progression-free survival and hazard ratio (HR), with non-DHL and non-DEL patients as reference categories. DEL=dual-expressor lymphoma. DHL=double-hit lymphoma. R-CHOP=rituximab, cyclophosphamide, doxorubicin, vincristine, and prednisolone. RB-CHOP=rituximab, bortezomib, cyclophosphamide, doxorubicin, vincristine, and prednisolone.

**Figure 4:**
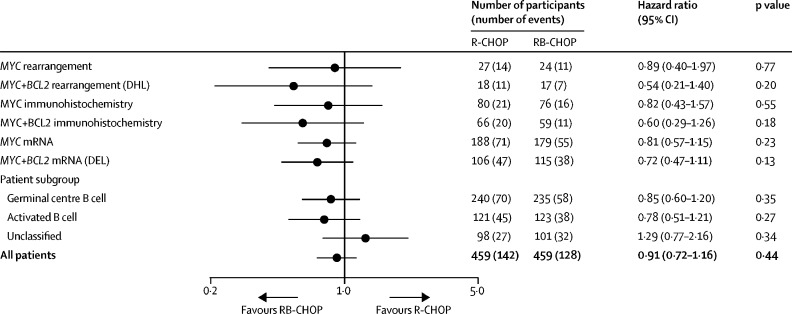
Forest plot of hazard ratios based on progression free survival for participants at high risk and with different molecular subtypes of disease, by treatment group Data are for all randomised participants (ie, ITT population). Hazard ratios and p values are effect estimates from a multivariable model adjusted for IPI score. DEL=dual-expressor lymphoma. DHL=double-hit lymphoma. IPI=international prognostic index. ITT=intention-to-treat. R-CHOP=rituximab, cyclophosphamide, doxorubicin, vincristine, and prednisolone. RB-CHOP=rituximab, bortezomib, cyclophosphamide, doxorubicin, vincristine, and prednisolone.

395 biopsy samples had sufficient DNA of adequate quantity and quality for investigation of mutations via targeted sequencing. 61 samples had to undergo a duplicate analysis because they were not of sufficient quality, and in 22 samples, mutations in 20 genes were further analysed. Among the participants given R-CHOP (including the non-randomised group), *MYC* rearrangement, double-hit lymphoma, and dual high *MYC* and *BCL-2* mRNA expression were significantly associated with inferior progression-free survival after controlling for IPI (data not shown). Participants in the R-CHOP group with either double-hit lymphoma (n=18) or dual high expression of *MYC* and *BCL-2* mRNA (ie, double-expressor lymphoma; n=102) had significantly worse progression-free survival at 30 months than participants in the same treatment group without these rearrangements (double-hit lymphoma in the R-CHOP group: 38·9% *vs* 75·8%, adjusted HR 3·07, 95% CI 1·64–5·76; p=0·00048; and dual-expressor lymphoma in the R-CHOP group: 61·5% *vs* 75·8%, adjusted HR 1·81, 1·26–2·60; p=0·0013; figure 3). High concentrations of MYC and BCL-2 proteins by immunohistochemistry did not appear to have a significant effect on outcomes, although few participants had high concentrations of these proteins, resulting in wide confidence limits (figure 4). The effect of bortezomib on progression-free survival in these high-risk groups is shown in figure 4.

We examined the effect of the addition of bortezomib on survival in patients with mutations known to be associated with activation of NF-κB, the putative target of bortezomib (*MYD88, CD79A, CD79B, TNFAIP3, TNFRSF11A*) and found no significant differences for single gene alterations (appendix p 5).

## Discussion

In this trial, we have shown the feasibility of molecular phenotyping in a large multicentre study of rapidly progressive tumours and shown that the addition of bortezomib does not affect treatment outcomes in most patients with diffuse large B-cell lymphoma. Because patients entering clinical trials are often not representative of the wider population, for prospective testing of a complex biomarker we wished to avoid worsening the problem of generalisability by restricted enrolment and delays to the initiation of therapy. Such delays were avoided by studying routine FFPE biopsy samples and deferring random assignment to a treatment group until the second cycle of R-CHOP, thereby allowing treatment to start as soon as staging investigations were completed, with molecular analysis taking place in parallel. Deferred introduction of the experimental drug had a further advantage, because more treatment-related deaths (around 62%) in diffuse large B-cell lymphoma treatment cycles are reported after the first cycle than at any other timepoint,^13^ a proportion that might increase with an additional drug.

To our knowledge, all previous studies of gene-expression profiling in lymphoma have been retrospective; assignment of patients to novel therapies on the basis of their molecular phenotype will require real-time outputs, which we have shown to be feasible in this trial. Using a central laboratory and the DASL automated classifier, we prospectively assigned cell-of-origin categories within a clinically relevant timeframe, which allowed random assignment to treatment to be stratified by cell-of-origin subtype, with the potential for adaptive design based on interim analyses of molecular subtypes. The accuracy of the classifier is supported by our identification of expected frequencies of different mutations that are known to be enriched in activated B-cell or germinal centre B-cell subtypes of disease.^14^ We identified that NF-κB target genes were more highly expressed in the activated B-cell subtype whereas almost all participants with double-hit lymphoma were identified within the germinal centre B-cell subgroup, which is consistent with the published literature.^15^ Dual expression of MYC and BCL-2 proteins or mRNA was more frequent in participants with the activated B-cell subtype than in those with the germinal centre B-cell subgroup, and at similar frequencies to those previously reported.^16^

The overall frequency of the activated B-cell subtype (27%) was lower than has been reported in some retrospective studies, in which approximately equal numbers of patients with germinal centre B-cell and activated B-cell lymphoma were seen.^15^, ^17^ However, a large randomised trial^18^ has reported very similar findings to our study, showing 58% of participants had germinal centre B-cell subtype, 26% activated B-cell, and 16% unclassifiable disease, by use of NanoString Lymphoma Subtyping Test.^18^ Some patients with lymphoma with poor prognostic features at presentation might be excluded from such trials on the grounds of performance status or the need for urgent treatment before screening procedures can be completed, which might reduce the proportion of patients with activated B-cell subtype disease entering prospective studies.

Our overall outcomes are consistent with those of other large prospective studies in diffuse large B-cell lymphoma, with similar progression-free survival (70·6% to 75·2% at 30 months) to other phase 3 trials.^2^, ^18^ The progression-free survival for the ITT population was not improved by the addition of bortezomib at the doses used in this study, and neither was a differential effect of treatment seen according to the cell of origin. This observation is in keeping with the findings of a smaller randomised phase 2 study, in which the addition of bortezomib 1·3 mg/m^2^ to R-CHOP on days 1 and 4 did not improve outcomes for non-germinal centre B-cell diffuse large B-cell lymphoma, defined in that study by immunohistochemistry.^19^

Administration of R-CHOP was not substantially compromised by the addition of bortezomib. Individual R-CHOP components had similar median relative dose intensities between groups and almost 90% of patients completed six cycles of treatment. The slight increase in neurotoxicity observed in participants in the RB-CHOP group compared with the R-CHOP group suggests that the bortezomib was given at a biologically active dose. Bortezomib was administered on days 1 and 8 of cycles 2–5, at a dose that has shown efficacy in other lymphoma trials,^19^, ^20^ but we recognise that more potent proteasome inhibitors are now in use, as are other agents with apparent preferential effects in the activated B-cell subgroup, such as lenalidomide and ibrutinib, trials of which are in progress (NCT02285062, NCT01855750).

Bortezomib did not improve outcomes for participants with the activated B-cell subtype of diffuse large B-cell lymphoma, which was confirmed to be enriched for expression of NF-κB target genes, or for patients with somatic mutations associated with NF-κB activation. Inhibition of NF-κB might be insufficient to improve outcomes in addition to R-CHOP in diffuse large B-cell lymphoma, or bortezomib at the doses given might not have been sufficient to inhibit NF-κB adequately for outcomes to improve. Another study in patients with non-germinal centre B-cell lymphoma, selected by immunohistochemistry, did not show a difference between R-CHOP and the combination of rituximab, cyclophosphamide, doxorubicin, and prednisone (R-CHP) with bortezomib given in place of vincristine, supporting our finding.^20^

Studies^18^, ^19^, ^20^ have shown the difficulty of improving the results of initial therapy in diffuse large B-cell lymphoma by the addition of novel drugs that had promising activity in studies treating recurrent disease with a single treatment group. This situation might partly reflect biological selection through treatment failure: in relapsed or refractory lymphomas for which new drugs are investigated, the biology of such disease is likely to be different from that of newly diagnosed lymphomas. Thus, most germinal centre B-cell diffuse large B-cell lymphoma can be cured by R-CHOP, whereas recurrent disease is more common for those with double-hit lymphoma. Similarly, recurrent activated B-cell diffuse large B-cell lymphoma is enriched for double-expressor lymphomas, which might account for the different results reported in our study compared with the previous studies of bortezomib treatment. This observation highlights the need for full molecular characterisation of the disease being treated, both at diagnosis and in the event of initial treatment failure.

In this study, the presence of a small number of participants with double-hit lymphoma in the germinal centre B-cell subgroup lowered the progression-free survival estimate for this subgroup. Overall, however, the progression-free survival outcome for the patients with double-hit lymphoma appears to be better than that reported in some earlier studies^21^ and is consistent with more recent analyses.^22^, ^23^ Although clearly worse than the non-rearranged group, nearly half of double-hit lymphomas appeared to have not progressed at 30 months. The progression-free survival at 30 months in patients with double-hit lymphoma was 38·9% after R-CHOP compared with 58·8% after RB-CHOP, although this result was from a post-hoc analysis and was not statistically significant (data not shown).

This study had several limitations. Any clinical trial is potentially prone to selective recruitment of those patients with better prognoses, but we endeavoured to minimise this effect by deferring random assignment to treatment until the second cycle of therapy, thereby allowing rapid initiation of treatment at the same time as molecular typing. As a result, the median time from diagnosis to initiation of therapy was lower than in similar studies,^24^ and the distribution of IPI scores in this study was similar to or worse than recent trials,^2^, ^18^ with 47% of patients being high-intermediate or high risk. Despite this limitation, the exclusion of patients with ECOG performance status of 3 or higher might have removed a cohort with the most adverse biology. We were unable to do a comprehensive central histopathology review on the participants enrolled and we did not assess for the presence of Epstein-Barr virus in the biopsy samples. However, all participants were diagnosed by expert haematopathologists by use of a procedure with high accuracy for diffuse large B-cell lymphoma (over 96% in a recent case series from the UK^25^), and because Epstein-Barr virus is present in less than 3% of diffuse large B-cell lymphoma in Europe,^26^ the presence of the virus would be unlikely to affect our results. The dose of bortezomib was chosen to reduce the risk of additive neurotoxicity, but as a result the dose might have been insufficient. The RB-CHOP group had a slight excess of vincristine dose reductions compared with the R-CHOP group, which could potentially have eroded a positive effect from the bortezomib. The use of routine FFPE biopsy samples was necessitated by the large number of recruiting centres, but resulted in a failure rate of about 15% for molecular typing and might have resulted in a larger than expected number of unclassified cases for whom poor quality RNA resulted in a low probability score in the cell-of-origin classifier.

In conclusion, this trial has shown that complex molecular characterisation can be done in real time, with a pragmatic treatment schedule that allows for the allocation of therapy on the basis of the molecular subtype. This method is likely to become increasingly relevant as our understanding of the phenotypic diversity of diffuse large B-cell lymphoma expands to encompass not only cell of origin, but also other biologically distinct categories based on genomic alterations.^15^, ^27^, ^28^ Future trials that use such methods will be important to explore the mechanisms of action of investigational drugs and to redefine the groups in which they are most likely to be effective. The poor prognosis of double-hit lymphomas could be seen as a potential opportunity for such an approach.

## Data sharing

Individual participant data will be made available, including data dictionaries, for approved data sharing requests. Individual participant data will be shared that underlie the results reported in this Article, after de-identification and normalisation of information (text, tables, figures, and appendices). The study protocol is available in the appendix (pp 15–90) and the statistical analysis plan will also be available upon request. Anonymised data will be available beginning 3 months after and ending 5 years after publication of this Article to researchers who provide a completed Data Sharing Agreement that describes a methodologically sound proposal for the purpose of the approved proposal. Proposals should be directed to ctu@soton.ac.uk. Data will be shared once all relevant parties approve and sign the Data Sharing Agreement. Data sharing requests are available for 5 years via the Southampton Clinical Trials Unit website.
