# Supplementary material for: Gene-expression profiling of bortezomib added to standard chemoimmunotherapy for diffuse large B-cell lymphoma (REMoDL-B): an open-label, randomised, phase 3 trial
Source: Lancet Oncol. 2019 May;20(5):649–62. doi: 10.1016/S1470-2045(18)30935-5 (PMC6494978; doi:10.1016/S1470-2045(18)30935-5)
Supplement: Supplementary appendix [file mmc1.pdf]

THE LANCET
Oncology

Supplementary appendix

This appendix formed part of the original submission and has been peer reviewed. We post it as supplied by the authors.

Supplement to: Davies A, Cummin T E, Barrans S, et al. Gene-expression profiling of bortezomib added to standard chemoimmunotherapy for diffuse large B-cell lymphoma (REMoDL-B): an open-label, randomised, phase 3 trial. *Lancet Oncol* 2019; published online April 1. http://dx.doi.org/10.1016/S1470-2045(18)30935-5.

Appendix

Appendix – Figures

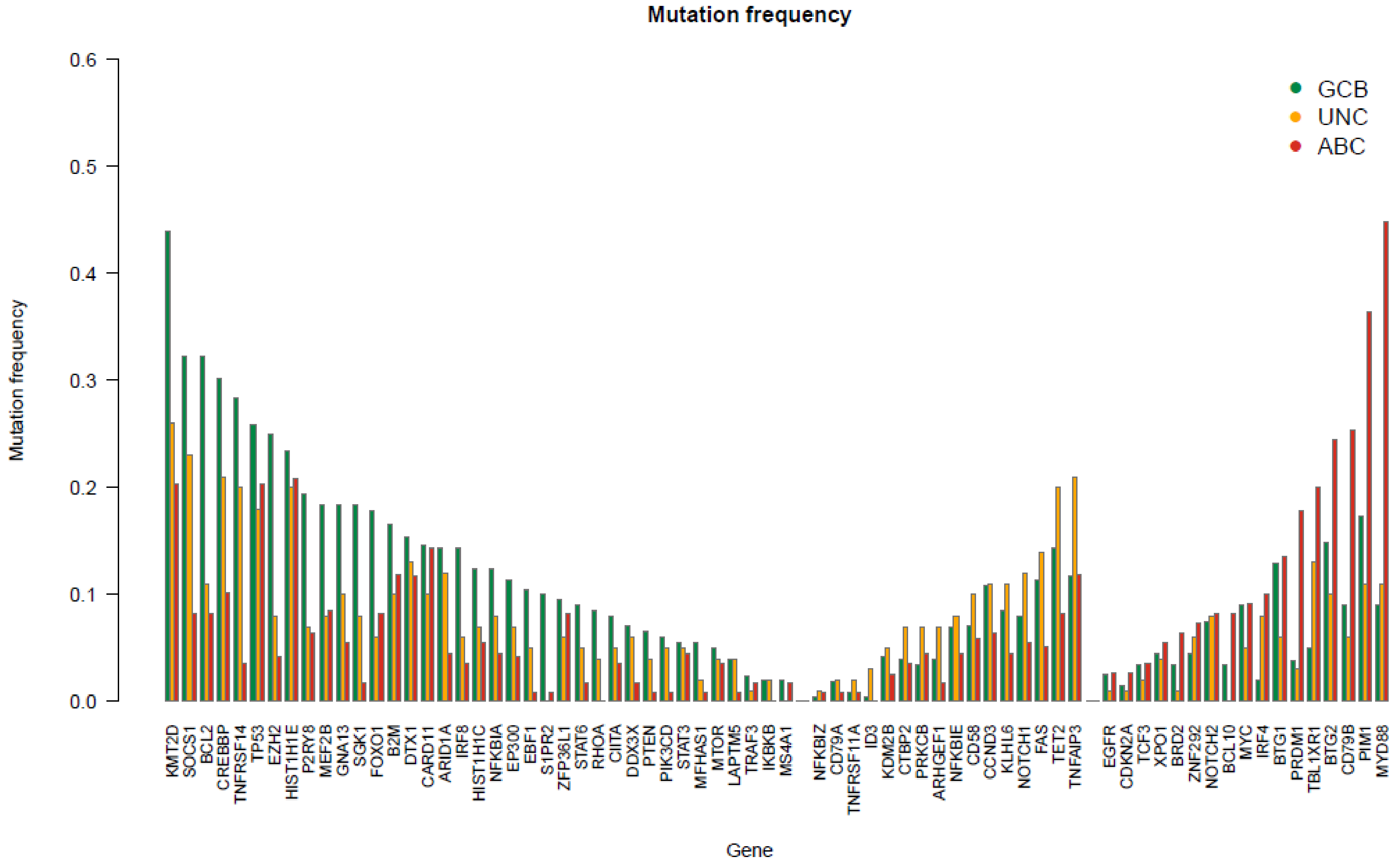

Appendix Figure 1: Mutation profile by Fluidigm multiplex-PCR Illumina sequencing or HaloPlexHS compared with COO subtype using Illumina WG-DASL array DAC classifierclassifier.

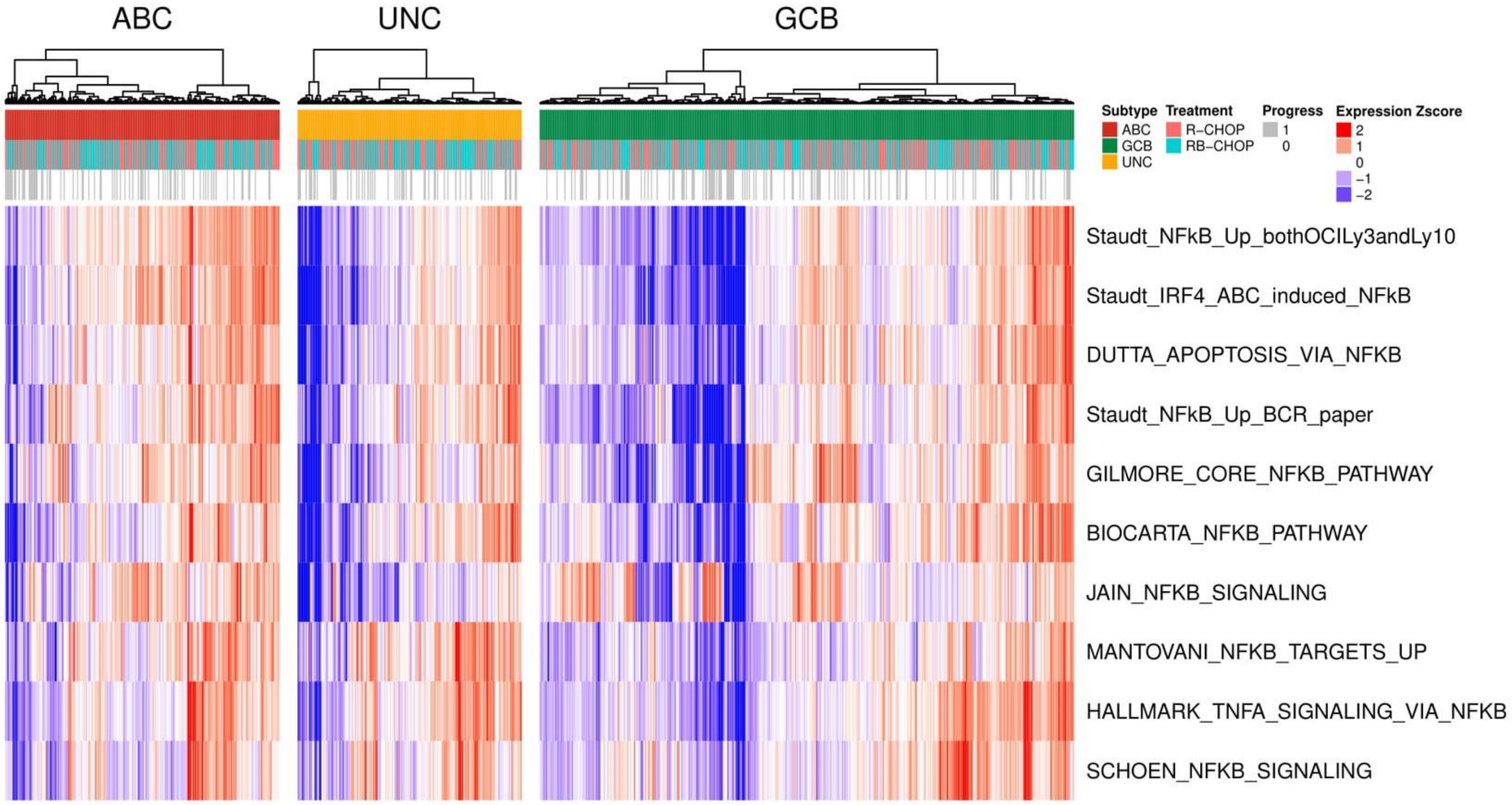

Appendix Figure 2: Gene expression heatmap of NF-kB pathway signatures / targets from MSigDB [1] and Staudt lab Signature DB in ABC, GCB and unclassified subgroups[2]. Z-score transformed mean expression of each signature is shown in the heatmap.

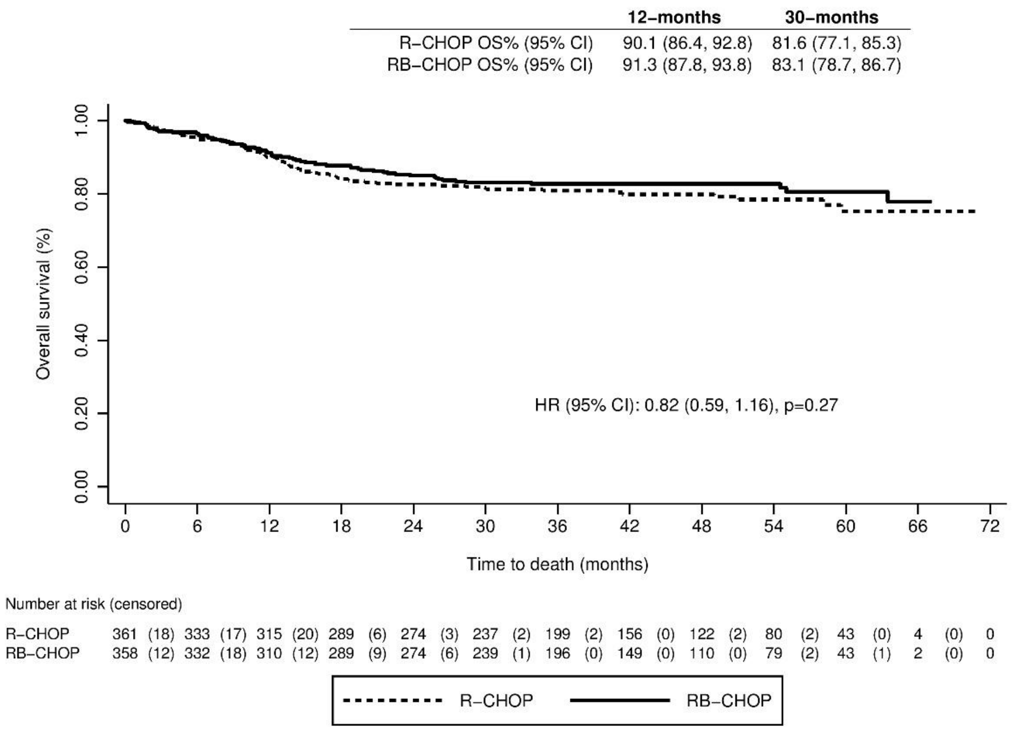

Appendix Figure 3: Overall survival according to treatment arm for ITT population (ABC and GCB patients)

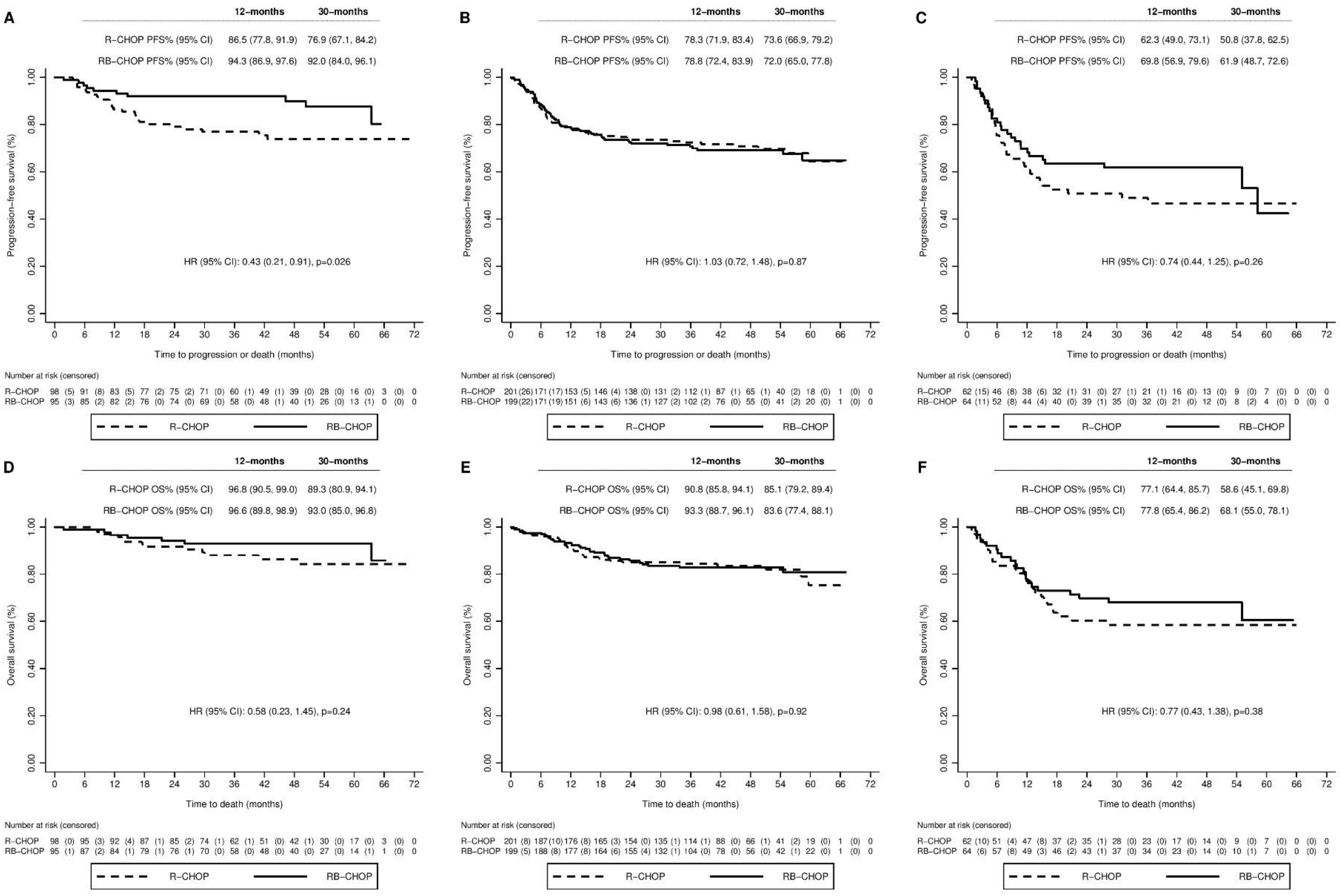

Appendix Figure 4. Analysis of PFS and OS by treatment arm in different risk groups according to the international prognostic index.
PFS: A: Low IPI; B: intermediate IPI; C: High IPI
OS: D: Low IPI; E: intermediate IPI; F: High IPI

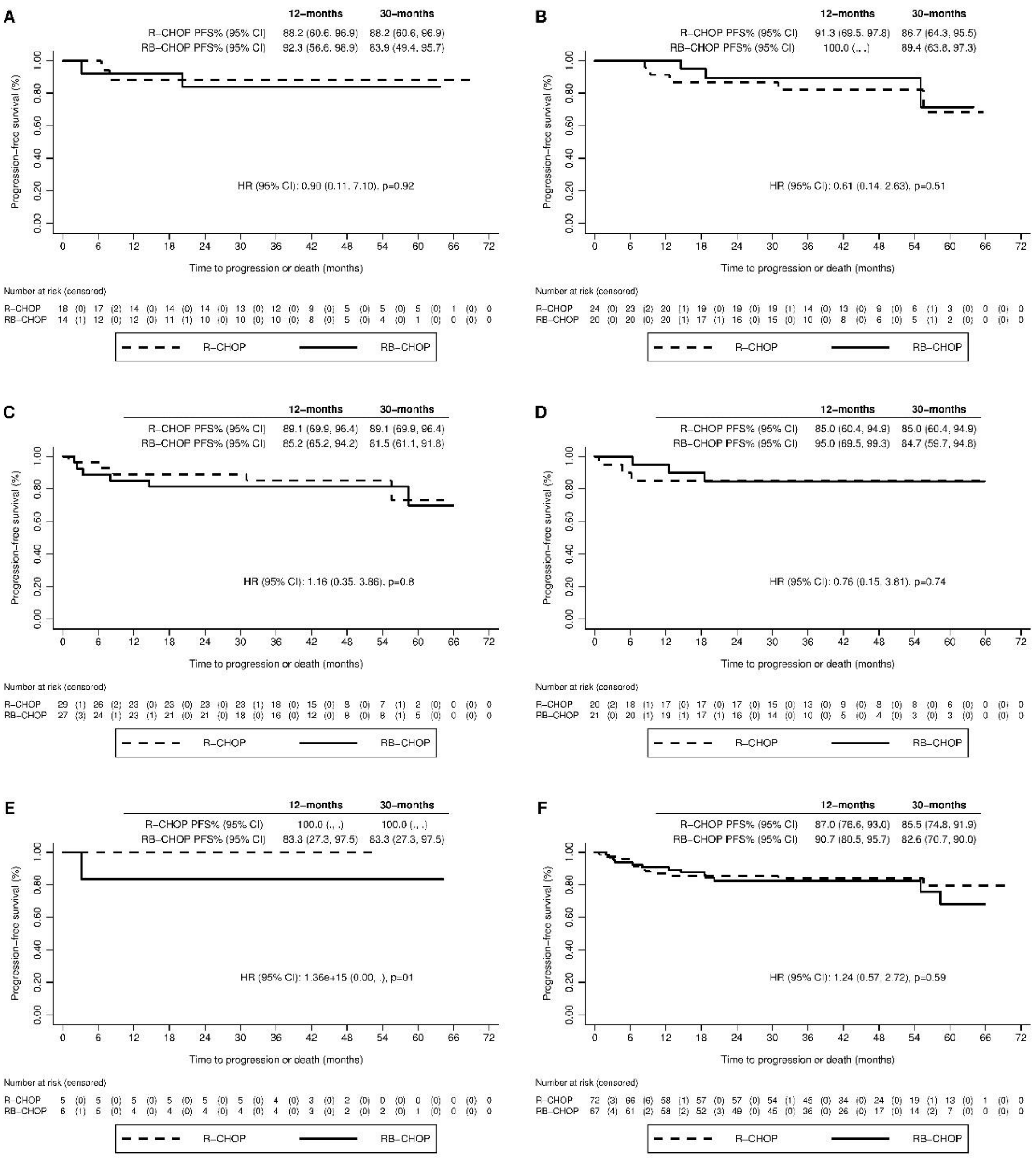

Appendix Figure 5: Kaplan-Meier curves for cases with somatic mutations identified related to activation of NF-κB and progression free survival by treatment arm, for: (A) *CARD11* subgroup; (B) *CD79A/B* subgroup; (C) *MYD88* subgroup; (D) *TNFAIP3 subgroup; (E) TNFRSF11A* subgroup; and (F) All NF-κB mutations subgroup.

Appendix - Tables

Site of involvement	ABC (n=244)	GCB (n=475)
Spleen	41 (16.8%)	86 (18.1%)
Liver	12 (4.9%)	35 (7.4%)
Lungs	28 (11.4%)	44 (9.3%)
Bone marrow	15 (6.1%)	54 (11.4%)
Kidney	12 (4.9%)	17 (3.6%)
Pericardium	1 (0.4%)	10 (2.1%)
Pleura	7 (2.8%)	38 (8.0%)
Skin	7 (2.8%)	5 (1.0%)
Testis	7 (2.8%)	1 (0.2%)
Other	62 (25.4%)	129 (27.2%)

Appendix Table 1. Numbers of patients with extranodal involvement according to molecular subtype

Gene	GCB	UNC	ABC	ABC vs GCB Fisher test p-value
KMT2D	0.439	0.26	0.203	4.91E-06
SOCS1	0.323	0.23	0.082	2.52E-08
BCL2	0.323	0.11	0.082	2.52E-08
CREBBP	0.302	0.21	0.102	3.15E-06
TNFRSF14	0.284	0.2	0.036	7.45E-11
TP53	0.259	0.18	0.203	2.43E-01
EZH2	0.25	0.08	0.042	8.90E-09
HIST1H1E	0.234	0.2	0.209	6.15E-01
P2RY8	0.194	0.07	0.064	4.04E-04
MEF2B	0.184	0.08	0.085	7.85E-03
GNA13	0.184	0.1	0.055	2.53E-04
SGK1	0.184	0.08	0.018	9.42E-08
FOXO1	0.179	0.06	0.082	1.04E-02
B2M	0.165	0.1	0.119	2.39E-01
DTX1	0.154	0.13	0.118	3.70E-01
CARD11	0.146	0.1	0.144	9.58E-01
ARID1A	0.144	0.12	0.045	2.11E-03
IRF8	0.144	0.06	0.036	4.93E-04
HIST1H1C	0.124	0.07	0.055	2.94E-02
NFKBIA	0.124	0.08	0.045	1.06E-02
EP300	0.113	0.07	0.042	1.40E-02
EBF1	0.104	0.05	0.009	6.34E-05
S1PR2	0.1	0	0.009	1.11E-04
ZFP36L1	0.095	0.06	0.082	7.04E-01
STAT6	0.09	0.05	0.018	3.06E-03
RHOA	0.085	0.04	0	2.68E-05
CIITA	0.08	0.05	0.036	1.00E-01
DDX3X	0.071	0.06	0.017	1.20E-02
PTEN	0.065	0.04	0.009	4.95E-03
PIK3CD	0.06	0.05	0.009	8.37E-03
STAT3	0.055	0.05	0.045	7.18E-01
MFHAS1	0.055	0.02	0.009	1.41E-02
MTOR	0.05	0.04	0.036	5.71E-01
LAPTM5	0.04	0.04	0.009	6.44E-02

Gene	GCB	UNC	ABC	ABC vs GCB Fisher test p-value
TRAF3	0.024	0.01	0.017	6.76E-01
IKBKB	0.02	0.02	0	4.52E-02
MS4A1	0.02	0	0.018	9.15E-01
NFKBIZ	0.005	0.01	0.009	6.92E-01
CD79A	0.019	0.02	0.008	4.11E-01
TNFRSF11A	0.009	0.02	0.008	9.29E-01
ID3	0.005	0.03	0	3.18E-01
KDM2B	0.042	0.05	0.025	3.98E-01
CTBP2	0.04	0.07	0.036	8.79E-01
PRKCB	0.035	0.07	0.045	6.56E-01
ARHGEF1	0.04	0.07	0.018	2.52E-01
NFKBIE	0.07	0.08	0.045	3.69E-01
CD58	0.071	0.1	0.059	6.84E-01
CCND3	0.109	0.11	0.064	1.55E-01
KLHL6	0.085	0.11	0.045	1.64E-01
NOTCH1	0.08	0.12	0.055	3.88E-01
FAS	0.113	0.14	0.051	3.72E-02
TET2	0.144	0.2	0.082	8.51E-02
TNFAIP3	0.118	0.21	0.119	9.85E-01
EGFR	0.025	0.01	0.027	9.00E-01
CDKN2A	0.015	0.01	0.027	4.89E-01
TCF3	0.035	0.02	0.036	9.45E-01
XPO1	0.045	0.04	0.055	7.10E-01
BRD2	0.035	0.01	0.064	2.83E-01
ZNF292	0.045	0.06	0.073	3.34E-01
NOTCH2	0.075	0.08	0.082	8.23E-01
BCL10	0.035	0	0.082	1.10E-01
MYC	0.09	0.05	0.091	9.68E-01
IRF4	0.02	0.08	0.1	9.36E-03
BTG1	0.129	0.06	0.136	8.63E-01
PRDM1	0.038	0.03	0.178	2.83E-04
TBL1XR1	0.05	0.13	0.2	3.79E-04
BTG2	0.149	0.1	0.245	4.79E-02
CD79B	0.09	0.06	0.254	3.17E-04
PIM1	0.174	0.11	0.364	4.81E-04
MYD88	0.09	0.11	0.449	2.33E-11

Appendix Table 2: Mutation frequency by Fluidigm multiplex-PCR and Illumina sequencing or HaloPlexHS compared with COO subtype using Illumina WG-DASL array DAC classifier.

Adverse events – n(%)[1]	R-CHOP (n=447)	RB-CHOP (n=444)	Total (n=891)
**Grade 1 or 2[2]**			
Number of patients experiencing at least 1 grade 1/2 AE	414 (92.6%)	415 (93.5%)	829 (93%)
Abdominal pain	61 (13.6%)	64 (14.4%)	125 (14%)
Alopecia	114 (25.5%)	106 (23.9%)	220 (24.7%)
Anaemia	73 (16.3%)	82 (18.5%)	155 (17.4%)
Anorexia	47 (10.5%)	55 (12.4%)	102 (11.4%)
Constipation	165 (36.9%)	180 (40.5%)	345 (38.7%)
Cough	53 (11.9%)	63 (14.2%)	116 (13%)
Diarrhoea	95 (21.3%)	133 (30%)	228 (25.6%)
Dyspnoea	56 (12.5%)	59 (13.3%)	115 (12.9%)
Fatigue	201 (45%)	191 (43%)	392 (44%)
Fever	64 (14.3%)	87 (19.6%)	151 (16.9%)
Mucositis	73 (16.3%)	62 (14%)	135 (15.2%)
Nausea	141 (31.5%)	165 (37.2%)	306 (34.3%)
Neutropenia	39 (8.7%)	53 (11.9%)	92 (10.3%)
Pain	56 (12.5%)	69 (15.5%)	125 (14%)
Peripheral sensory neuropathy	129 (28.9%)	182 (41%)	311 (34.9%)
Vomiting	63 (14.1%)	109 (24.5%)	172 (19.3%)
**Grade 3**			
Number of patients experiencing at least 1 grade 3 AE	226 (50.6%)	253 (57%)	479 (53.8%)
Abdominal and back pain	1 (0.2%)	1 (0.2%)	2 (0.2%)
Abdominal infection	1 (0.2%)	1 (0.2%)	2 (0.2%)
Abdominal pain	12 (2.7%)	9 (2%)	21 (2.4%)
Acute appendicitis	1 (0.2%)	0 (0%)	1 (0.1%)
Acute coronary syndrome	1 (0.2%)	0 (0%)	1 (0.1%)
Acute kidney injury	0 (0%)	1 (0.2%)	1 (0.1%)
Agitation	1 (0.2%)	1 (0.2%)	2 (0.2%)
Alanine aminotransferase increased	1 (0.2%)	1 (0.2%)	2 (0.2%)
Alkaline phosphatase increased	1 (0.2%)	0 (0%)	1 (0.1%)
Allergic reaction (metaclopramide)	0 (0%)	1 (0.2%)	1 (0.1%)
Alopecia	9 (2%)	6 (1.4%)	15 (1.7%)
Anaemia	19 (4.3%)	14 (3.2%)	33 (3.7%)
Anal pain	0 (0%)	1 (0.2%)	1 (0.1%)
Anorexia	6 (1.3%)	5 (1.1%)	11 (1.2%)
Anxiety	1 (0.2%)	0 (0%)	1 (0.1%)
Arthralgia	0 (0%)	1 (0.2%)	1 (0.1%)
Arthritis (gout)	1 (0.2%)	0 (0%)	1 (0.1%)
Ascites	0 (0%)	1 (0.2%)	1 (0.1%)
Aspartate aminotransferase increased	1 (0.2%)	1 (0.2%)	2 (0.2%)
Atrial fibrillation	0 (0%)	2 (0.5%)	2 (0.2%)
Back pain	6 (1.3%)	0 (0%)	6 (0.7%)
Bladder cancer	0 (0%)	1 (0.2%)	1 (0.1%)
Bladder infection	1 (0.2%)	0 (0%)	1 (0.1%)
Blood bilirubin increased	0 (0%)	1 (0.2%)	1 (0.1%)
Blurred vision	0 (0%)	1 (0.2%)	1 (0.1%)
Bone pain	2 (0.4%)	0 (0%)	2 (0.2%)
Bowel obstruction	1 (0.2%)	1 (0.2%)	2 (0.2%)
Bowel obstruction caused by neuroendocrine tumour	1 (0.2%)	0 (0%)	1 (0.1%)
Bowel perforation	0 (0%)	1 (0.2%)	1 (0.1%)
Bronchial obstruction	0 (0%)	1 (0.2%)	1 (0.1%)
Cardial troponin increased	1 (0.2%)	0 (0%)	1 (0.1%)
Catherter related infection	0 (0%)	1 (0.2%)	1 (0.1%)
Cellulitis	1 (0.2%)	1 (0.2%)	2 (0.2%)
Chest infection	3 (0.7%)	1 (0.2%)	4 (0.4%)
Chest pain - cardiac	6 (1.3%)	4 (0.9%)	10 (1.1%)
Chills	0 (0%)	1 (0.2%)	1 (0.1%)
Cholecystitis	2 (0.4%)	0 (0%)	2 (0.2%)
Clostridium difficile infection	1 (0.2%)	1 (0.2%)	2 (0.2%)
Collapse/fall	4 (0.9%)	2 (0.5%)	6 (0.7%)
Colonic perforation	1 (0.2%)	0 (0%)	1 (0.1%)
Confusion	1 (0.2%)	1 (0.2%)	2 (0.2%)
Constipation	1 (0.2%)	5 (1.1%)	6 (0.7%)
Corneal ulcer	0 (0%)	1 (0.2%)	1 (0.1%)
Cough	0 (0%)	1 (0.2%)	1 (0.1%)
Creatinine increased	1 (0.2%)	0 (0%)	1 (0.1%)
Dehydration	2 (0.4%)	4 (0.9%)	6 (0.7%)
Dental caries	1 (0.2%)	0 (0%)	1 (0.1%)
Depression	1 (0.2%)	0 (0%)	1 (0.1%)
Deranged liver function tests	0 (0%)	1 (0.2%)	1 (0.1%)
Deranged renal function	0 (0%)	1 (0.2%)	1 (0.1%)
Diarrhoea	10 (2.2%)	24 (5.4%)	34 (3.8%)
Diarrhoea and gastroenteritis	0 (0%)	1 (0.2%)	1 (0.1%)

Adverse events – n(%)[1]	R-CHOP (n=447)	RB-CHOP (n=444)	Total (n=891)
Disc herniation	1 (0.2%)	0 (0%)	1 (0.1%)
Disinhibition	0 (0%)	1 (0.2%)	1 (0.1%)
Dizziness	1 (0.2%)	3 (0.7%)	4 (0.4%)
Duodenal obstruction	1 (0.2%)	0 (0%)	1 (0.1%)
Dysgeusia	1 (0.2%)	0 (0%)	1 (0.1%)
Dysphagia	1 (0.2%)	1 (0.2%)	2 (0.2%)
Dyspnoea	4 (0.9%)	4 (0.9%)	8 (0.9%)
E coli bacteraemia	1 (0.2%)	0 (0%)	1 (0.1%)
Ear pain	1 (0.2%)	0 (0%)	1 (0.1%)
Electrolyte imbalance and malnutrition	1 (0.2%)	0 (0%)	1 (0.1%)
Elevated bilirubin	1 (0.2%)	0 (0%)	1 (0.1%)
Elevated liver function tests	0 (0%)	1 (0.2%)	1 (0.1%)
Encephalopathy	0 (0%)	1 (0.2%)	1 (0.1%)
Eye injury	0 (0%)	1 (0.2%)	1 (0.1%)
Facial muscle weakness	0 (0%)	1 (0.2%)	1 (0.1%)
Fatigue	10 (2.2%)	8 (1.8%)	18 (2%)
Febrile neutropenia	49 (11%)	51 (11.5%)	100 (11.2%)
Fever	17 (3.8%)	14 (3.2%)	31 (3.5%)
Fracture	5 (1.1%)	1 (0.2%)	6 (0.7%)
Gastric haemorrhage	1 (0.2%)	1 (0.2%)	2 (0.2%)
Gastric stenosis	1 (0.2%)	2 (0.5%)	3 (0.3%)
Gastrointestinal bleed	2 (0.4%)	0 (0%)	2 (0.2%)
General detrioriation	0 (0%)	1 (0.2%)	1 (0.1%)
Glucose intolerance	1 (0.2%)	0 (0%)	1 (0.1%)
Haemorrhoids	0 (0%)	1 (0.2%)	1 (0.1%)
Headache	6 (1.3%)	3 (0.7%)	9 (1%)
Hearing impaired	0 (0%)	1 (0.2%)	1 (0.1%)
Heart failure	2 (0.4%)	1 (0.2%)	3 (0.3%)
Hepatic infection	1 (0.2%)	0 (0%)	1 (0.1%)
Hickman line infection	1 (0.2%)	0 (0%)	1 (0.1%)
Hyperglycaemia	0 (0%)	2 (0.5%)	2 (0.2%)
Hyperkalaemia	1 (0.2%)	0 (0%)	1 (0.1%)
Hypertension	4 (0.9%)	0 (0%)	4 (0.4%)
Hypertriglyceridemia	0 (0%)	1 (0.2%)	1 (0.1%)
Hypoalbuminaemia	0 (0%)	3 (0.7%)	3 (0.3%)
Hypocalcaemia	0 (0%)	1 (0.2%)	1 (0.1%)
Hypokalaemia	1 (0.2%)	6 (1.4%)	7 (0.8%)
Hypomagnesaemia	0 (0%)	1 (0.2%)	1 (0.1%)
Hyponatraemia	0 (0%)	4 (0.9%)	4 (0.4%)
Hypophosphataemia	1 (0.2%)	5 (1.1%)	6 (0.7%)
Hypotension	6 (1.3%)	7 (1.6%)	13 (1.5%)
Hypoxia	0 (0%)	1 (0.2%)	1 (0.1%)
Infection	4 (0.9%)	13 (2.9%)	17 (1.9%)
Infusion related reaction	0 (0%)	1 (0.2%)	1 (0.1%)
Insomnia	0 (0%)	2 (0.5%)	2 (0.2%)
Kidney infection	1 (0.2%)	0 (0%)	1 (0.1%)
Left basal creps	1 (0.2%)	0 (0%)	1 (0.1%)
Left ventricular systolic dysfunction	0 (0%)	2 (0.5%)	2 (0.2%)
Lethargy	1 (0.2%)	4 (0.9%)	5 (0.6%)
Leukopenia	1 (0.2%)	1 (0.2%)	2 (0.2%)
Lower respiratory tract infection	2 (0.4%)	2 (0.5%)	4 (0.4%)
Lumbar radicular syndrome	1 (0.2%)	0 (0%)	1 (0.1%)
Lung infection	8 (1.8%)	16 (3.6%)	24 (2.7%)
Lymph gland infection	1 (0.2%)	1 (0.2%)	2 (0.2%)
Lymphocyte count decreased	5 (1.1%)	17 (3.8%)	22 (2.5%)
Lymphopenia	0 (0%)	1 (0.2%)	1 (0.1%)
Meningitis	0 (0%)	1 (0.2%)	1 (0.1%)
Mucositis	2 (0.4%)	6 (1.4%)	8 (0.9%)
Mucositis oral	4 (0.9%)	4 (0.9%)	8 (0.9%)
Myocardial infarction	1 (0.2%)	1 (0.2%)	2 (0.2%)
Nausea	3 (0.7%)	8 (1.8%)	11 (1.2%)
Neoplasm benign malignant & unsuspected - lung primary lesion	1 (0.2%)	1 (0.2%)	2 (0.2%)
Neuropathy (motor and sensory)	0 (0%)	1 (0.2%)	1 (0.1%)
Neuropathy	0 (0%)	2 (0.5%)	2 (0.2%)
Neutropenia	56 (12.5%)	86 (19.4%)	142 (15.9%)
Neutropenic sepsis	9 (2%)	19 (4.3%)	28 (3.1%)
Neutrophil count decreased	1 (0.2%)	0 (0%)	1 (0.1%)
Non-cardiac chest pain	4 (0.9%)	0 (0%)	4 (0.4%)
Oedema limbs	1 (0.2%)	0 (0%)	1 (0.1%)
Osteoporosis	1 (0.2%)	0 (0%)	1 (0.1%)
Other - Aneurysm	0 (0%)	1 (0.2%)	1 (0.1%)
Other - Cholangitis	1 (0.2%)	0 (0%)	1 (0.1%)
Other - Clostridium difficile	1 (0.2%)	3 (0.7%)	4 (0.4%)
Other - Extravasation	0 (0%)	1 (0.2%)	1 (0.1%)

Adverse events – n(%)[1]	R-CHOP (n=447)	RB-CHOP (n=444)	Total (n=891)
Other - Gout	1 (0.2%)	0 (0%)	1 (0.1%)
Other - Increased CRP	0 (0%)	2 (0.5%)	2 (0.2%)
Other - Infection of unknown cause	0 (0%)	1 (0.2%)	1 (0.1%)
Other - Lung primary neoplasm	0 (0%)	1 (0.2%)	1 (0.1%)
Other - Perseveration	0 (0%)	1 (0.2%)	1 (0.1%)
Other - Scortal infection	1 (0.2%)	0 (0%)	1 (0.1%)
Other - Varicella zoster infection	0 (0%)	1 (0.2%)	1 (0.1%)
Otitis media	0 (0%)	1 (0.2%)	1 (0.1%)
Pain	5 (1.1%)	6 (1.4%)	11 (1.2%)
Paraesthesia	2 (0.4%)	1 (0.2%)	3 (0.3%)
Peripharal nerve infection	0 (0%)	1 (0.2%)	1 (0.1%)
Peripheral motor neuropathy	2 (0.4%)	5 (1.1%)	7 (0.8%)
Peripheral neuropathy	1 (0.2%)	2 (0.5%)	3 (0.3%)
Peripheral sensory neuropathy	3 (0.7%)	8 (1.8%)	11 (1.2%)
Platelet count decreased	2 (0.4%)	4 (0.9%)	6 (0.7%)
Pleural effusion	3 (0.7%)	2 (0.5%)	5 (0.6%)
Pleural infection	0 (0%)	1 (0.2%)	1 (0.1%)
Pleuritic pain	0 (0%)	1 (0.2%)	1 (0.1%)
Productive cough	3 (0.7%)	4 (0.9%)	7 (0.8%)
Rash maculo-papular	1 (0.2%)	0 (0%)	1 (0.1%)
Respiratory infection	1 (0.2%)	0 (0%)	1 (0.1%)
Respiratory tract infection	0 (0%)	1 (0.2%)	1 (0.1%)
Right hip arthoroplasty	0 (0%)	1 (0.2%)	1 (0.1%)
Rigors	0 (0%)	1 (0.2%)	1 (0.1%)
Sepsis	2 (0.4%)	2 (0.5%)	4 (0.4%)
Sinusitis	1 (0.2%)	0 (0%)	1 (0.1%)
Skin infection	1 (0.2%)	9 (2%)	10 (1.1%)
Skin lesions to face/jaw	0 (0%)	1 (0.2%)	1 (0.1%)
Small intestinal obstruction	0 (0%)	1 (0.2%)	1 (0.1%)
Small intestinal perforation	0 (0%)	1 (0.2%)	1 (0.1%)
Soft tissue infection	1 (0.2%)	0 (0%)	1 (0.1%)
Sore throat	3 (0.7%)	2 (0.5%)	5 (0.6%)
Sore throat and cough	1 (0.2%)	0 (0%)	1 (0.1%)
Spinal herniation	1 (0.2%)	0 (0%)	1 (0.1%)
Splenic abscess	1 (0.2%)	0 (0%)	1 (0.1%)
Steroid withdrawal	1 (0.2%)	0 (0%)	1 (0.1%)
Stomach pain	0 (0%)	1 (0.2%)	1 (0.1%)
Supraventricular tachycardia	0 (0%)	1 (0.2%)	1 (0.1%)
Syncope	5 (1.1%)	7 (1.6%)	12 (1.3%)
Thrombocytopenia	3 (0.7%)	3 (0.7%)	6 (0.7%)
Thromboembolic event	9 (2%)	6 (1.4%)	15 (1.7%)
Tooth infection	1 (0.2%)	1 (0.2%)	2 (0.2%)
Upper respiratory infection	3 (0.7%)	5 (1.1%)	8 (0.9%)
Urinary retention	0 (0%)	2 (0.5%)	2 (0.2%)
Urinary tract infection	5 (1.1%)	7 (1.6%)	12 (1.3%)
Vascular access complication	1 (0.2%)	0 (0%)	1 (0.1%)
Vasovagal reaction	1 (0.2%)	1 (0.2%)	2 (0.2%)
Vertigo	0 (0%)	1 (0.2%)	1 (0.1%)
Vomiting	6 (1.3%)	11 (2.5%)	17 (1.9%)
Weight loss	4 (0.9%)	1 (0.2%)	5 (0.6%)
White blood cell decreased	7 (1.6%)	22 (5%)	29 (3.3%)
Wound dehiscence	0 (0%)	1 (0.2%)	1 (0.1%)
Wound infection	0 (0%)	1 (0.2%)	1 (0.1%)
**Grade 4**			
Number of patients experiencing at least 1 grade 4 AE	107 (23.9%)	105 (23.6%)	212 (23.8%)
Abdominal pain	1 (0.2%)	0 (0%)	1 (0.1%)
Acute coronary syndrome	2 (0.4%)	0 (0%)	2 (0.2%)
Aspartate aminotransferase increased	1 (0.2%)	0 (0%)	1 (0.1%)
Atrial fibrillation	1 (0.2%)	0 (0%)	1 (0.1%)
Bowel obstruction	1 (0.2%)	1 (0.2%)	2 (0.2%)
Colonic perforation	1 (0.2%)	0 (0%)	1 (0.1%)
Duodenal obstruction	0 (0%)	1 (0.2%)	1 (0.1%)
Febrile neutropenia	14 (3.1%)	9 (2%)	23 (2.6%)
Fever	1 (0.2%)	1 (0.2%)	2 (0.2%)
Gastric stenosis	1 (0.2%)	0 (0%)	1 (0.1%)
Heart failure	1 (0.2%)	0 (0%)	1 (0.1%)
Hyperglycaemia	0 (0%)	2 (0.5%)	2 (0.2%)
Hypoglycaemia	0 (0%)	1 (0.2%)	1 (0.1%)
Hypomagnesaemia	0 (0%)	1 (0.2%)	1 (0.1%)
Hyponatraemia	0 (0%)	1 (0.2%)	1 (0.1%)
Hypophosphataemia	0 (0%)	1 (0.2%)	1 (0.1%)
Hypotension	2 (0.4%)	0 (0%)	2 (0.2%)
Jejunal stricture with small bowel obstruction	0 (0%)	1 (0.2%)	1 (0.1%)
Leukaemia secondary to oncology chemotherapy	1 (0.2%)	0 (0%)	1 (0.1%)

Adverse events – n(%)[1]	R-CHOP (n=447)	RB-CHOP (n=444)	Total (n=891)
Leukopenia	1 (0.2%)	1 (0.2%)	2 (0.2%)
Lung infection	0 (0%)	1 (0.2%)	1 (0.1%)
Lymphocyte count decreased	3 (0.7%)	4 (0.9%)	7 (0.8%)
Lymphopenia	0 (0%)	2 (0.5%)	2 (0.2%)
Myelodysplastic syndrome	1 (0.2%)	0 (0%)	1 (0.1%)
Myocardial infarction	1 (0.2%)	0 (0%)	1 (0.1%)
Nausea	0 (0%)	1 (0.2%)	1 (0.1%)
Neutropenia	64 (14.3%)	69 (15.5%)	133 (14.9%)
Neutropenic sepsis	23 (5.1%)	11 (2.5%)	34 (3.8%)
Non-cardiac chest pain	1 (0.2%)	0 (0%)	1 (0.1%)
Obesity	1 (0.2%)	0 (0%)	1 (0.1%)
Other - Prostate adenocarcinoma	1 (0.2%)	0 (0%)	1 (0.1%)
Other - Unknown	0 (0%)	1 (0.2%)	1 (0.1%)
Pancytopenia	1 (0.2%)	0 (0%)	1 (0.1%)
Platelet count decreased	0 (0%)	5 (1.1%)	5 (0.6%)
Renal failure	0 (0%)	1 (0.2%)	1 (0.1%)
Sepsis	12 (2.7%)	15 (3.4%)	27 (3%)
Severe metabolic disorder	0 (0%)	1 (0.2%)	1 (0.1%)
Sinus bradycardia	1 (0.2%)	0 (0%)	1 (0.1%)
Thrombocytopenia	2 (0.4%)	2 (0.5%)	4 (0.4%)
Thromboembolic event	1 (0.2%)	3 (0.7%)	4 (0.4%)
Tumour lysis syndrome	0 (0%)	1 (0.2%)	1 (0.1%)
White blood cell decreased	7 (1.6%)	8 (1.8%)	15 (1.7%)
**Grade 5**			
Number of patients experiencing at least 1 grade 5 AE	6 (1.3%)	4 (0.9%)	10 (1.1%)
Bowel perforation	1 (0.2%)	0 (0%)	1 (0.1%)
Haemophagocytosis syndrome	1 (0.2%)	0 (0%)	1 (0.1%)
Lung infection	0 (0%)	1 (0.2%)	1 (0.1%)
Neutropenic sepsis	1 (0.2%)	1 (0.2%)	2 (0.2%)
Other - Pneumocystis cairnii pneumonia	1 (0.2%)	0 (0%)	1 (0.1%)
Pneumocystis cairnii pneumonia	1 (0.2%)	0 (0%)	1 (0.1%)
Pulmonary fibrosis	1 (0.2%)	0 (0%)	1 (0.1%)
Renal failure	0 (0%)	1 (0.2%)	1 (0.1%)
Thromboembolic event	1 (0.2%)	1 (0.2%)	2 (0.2%)
Type ii respiratory failure	0 (0%)	1 (0.2%)	1 (0.1%)

Appendix Table 3: Adverse events (safety population).
[1] Information calculated using the number of patients receiving R-CHOP/RB-CHOP.
[2] Only adverse events in which ≥10% of patients experienced are reported for Grade 1 or 2 adverse events.

Treatment information	R-CHOP (n=459)	RB-CHOP (n=459)	Total (n=918)
Discontinued treatment – n(%)[1]	43 (9.4%)	60 (13.1%)	103 (11.2%)
**Reason – n(%)[2]**			
Adverse Event	5 (11.6%)	6 (10%)	11 (10.7%)
Clinician Decision	11 (25.6%)	10 (16.7%)	21 (20.4%)
Patient Decision	3 (7%)	17 (28.3%)	20 (19.4%)
Protocol Violation	1 (2.3%)	1 (1.7%)	2 (1.9%)
Other	23 (53.5%)	26 (43.3%)	49 (47.6%)

Appendix Table 4: Premature discontinuation of treatment information (ITT population).
[1] Information calculated using the number of patients receiving R-CHOP/RB-CHOP.
[2] Information calculated using the number of patients receiving R-CHOP/RB-CHOP who prematurely discontinued treatment.

	R-CHOP (n=459)	RB-CHOP (n=459)	Total (n=918)
Dosing Information available – n(%)[1]	458 (99.8%)	457 (99.6%)	915 (99.7%)
**Dosing Information**			
Received all 6 treatment cycles – n (%)[2]	418 (91.3%)	398 (87.1%)	816 (89.2%)
Any dose reduction[3] made – n (%)[2]	158 (34.5%)	196 (42.9%)	354 (38.7%)
Any Rituximab dose reduction made – n (%)[2]	37 (8.1%)	63 (13.8%)	100 (10.9%)
Any Cyclophosphamide dose reduction made – n (%)[2]	76 (16.6%)	73 (16.0%)	149 (16.3%)
Any Doxorubicin dose reduction made – n (%)[2]	79 (17.2%)	81 (17.7%)	160 (17.5%)
Any Vincristine dose reduction made – n (%)[2]	86 (18.8%)	136 (29.8%)	222 (24.3%)
Any Bortezomib dose reduction made – n (%)[2]	-	203 (44.4%)	-
Any R-CHOP dose delay made – n(%)[2]	197 (43.0%)	202 (44.2%)	399 (43.6%)
Rituximab RDI[2]	98.65	97.86	98.25
IQR	94.09 to 101.91	92.69 to 100.90	93.58 to 101.27
Cyclophosphamide RDI[2]	99.23	98.38	98.79
IQR	94.26 to 101.01	92.36 to 100.57	92.97 to 100.76
Doxorubicin RDI[2]	99.06	98.36	98.78
IQR	93.44 to 101.08	92.29 to 100.44	92.81 to 100.76
Vincristine RDI[2]	100.00	98.13	99.06
IQR	91.30 to 100.00	87.50 to 100.00	88.24 to 100.00
Bortezomib RDI[2]	-	93.60	-
IQR		80.34 to 99.66	

Appendix Table 5: Protocol compliance for administration by treatment arm for administered chemotherapy.

[1] Information calculated using the number of patients receiving R-CHOP/RB-CHOP.

[2] Information calculated using the number of patients receiving R-CHOP/RB-CHOP with dosing information available.

[3] Any dose reduction of Rituximab, Cyclophosphamide, Doxorubicin, or Vincristine recorded in Cycles 2 to 6.

Death information – n (%)[1]	R-CHOP (n=447)	RB-CHOP (n=444)	Total (n=891)
Died – n(%)[1]	73 (16.3%)	68 (15.3%)	141 (15.8%)
**Cause of death – n(%)[2]**			
Progressive lymphoma	50 (68.5%)	54 (79.4%)	104 (73.8%)
Other malignancy	4 (5.5%)	1 (1.5%)	5 (3.5%)
Cardiac death	2 (2.7%)	1 (1.5%)	3 (2.1%)
Multi organ failure	1 (1.4%)	0	1 (0.7%)
Treatment related toxicity	5 (6.8%)	4 (5.9%)	9 (6.4%)
Other: acute kidney injury	1 (1.4%)	0	1 (0.7%)
Other: acute necrotising pancreatitis	1 (1.4%)	0	1 (0.7%)
Other: bowel perforation	1 (1.4%)	1 (1.5%)	2 (1.4%)
Other: diarrhoea	0	1 (1.5%)	1 (0.7%)
Other: disseminated aspergillosis	0	0	0 (0%)
Other: idiopathic pulmonary fibrosis	1 (1.4%)	0	1 (0.7%)
Other: infective exacerbation of copd and influenza	0	0	0 (0%)
Other: intra-abdominal haemorrhage	1 (1.4%)	0	1 (0.7%)
Other: intracranial haemorrhage	1 (1.4%)	0	1 (0.7%)
Other: pneumocystis carinii pneumonia	1 (1.4%)	0	1 (0.7%)
Other: pneumonia	1 (1.4%)	4 (5.9%)	5 (3.5%)
Other: pulmonary embolism	2 (2.7%)	0	2 (1.4%)
Other: sepsis	0	2 (2.9%)	2 (1.4%)
Other: unknown - died outside of hospital	1 (1.4%)	0	1 (0.7%)

Appendix Table 6: Cause of death information (safety population).

[1] Information calculated using the number of patients receiving R-CHOP/RB-CHOP.

[2] Information calculated using the number of patients receiving R-CHOP/RB-CHOP who died.

Molecular phenotype	ABC	GCB	Unclassified	Fail	ABC vs GCB (Fisher's exact test p value)
**Double-hit lymphoma**	1 (0.4)	32 (6.7)	2 (1)	0 (0)	<0.0001
**Dual expressor lymphoma (IHC) (excluding DHLs)**	56 (54.9)	45 (26)	24 (19.7)	1 (50)	<0.0001
**Dual expressor lymphoma (mRNA) (Excluding DHLs)**	109 (44.7)	87 (18.3)	25 (12.6)	2 (20)	<0.0001
**High BCL-2 (IHC)**	89 (73)	120 (69.4)	47 (61)	2 (66.7)	0.00070
**High BCL-2 (mRNA)**	173 (70.9)	196 (41.3)	89 (44.7)	6 (60)	<0.0001

Appendix Table 7: Frequency of cases characterised by double-hit, dual-expressor and BCL-2 expression frequency in COO subtypes

Appendix – References (for Appendix)

1. Subramanian A, Tamayo P, Mootha VK, et al. Gene set enrichment analysis: a knowledge-based approach for interpreting genome-wide expression profiles. *Proceedings of the National Academy of Sciences* 2005; **102**(43): 15545-50.
2. Shaffer AL, Wright G, Yang L, et al. A library of gene expression signatures to illuminate normal and pathological lymphoid biology. *Immunological reviews* 2006; **210**(1): 67-85.

A Randomised Evaluation of Molecular Guided Therapy for Diffuse Large B-cell Lymphoma with Bortezomib

REMoDL-B

Version number 10
06 October 2016

SPONSOR: University Hospital Southampton NHS Foundation Trust

COORDINATING CENTRE: Southampton Clinical Trials Unit

EudraCT reference no:	2010-022422-32
ISRCTN No	51837425
Ethics reference no:	10/H0504/79

Protocol authorised by:

Name: Professor Peter Johnson **Role:** Chief Investigator

Signature: **Date:**

Name: Professor Gareth Griffiths **Role:** Director of SCTU

Signature: **Date:**

Trial Development/Management Group Members

Chief Investigator:

Professor Peter Johnson Cancer Research UK Centre Somers Cancer Building Southampton General Hospital

Dr Andrew Davies
Cancer Research UK Centre
Somers Cancer Building
Southampton General Hospital

Pathology Lead:

Dr Andrew Jack
Haematological Malignancy Diagnostic Service
St James's Institute of Oncology
Leeds Teaching Hospitals NHS Trust
St James's University Hospital

Dr Paul Fields
Department of Haematology
Kings Health Partners AHSC
4th Floor Southwark Wing
Guy's Hospital

Dr Christoph Mamot
Centre for Oncology, Haematology and Transfusion Medicine

Professor Ming-Qing Du
Department of Pathology
University of Cambridge

Dr Andrew McMillan
Department of Haematology
Nottingham City Hospital

Study Statisticians:

Louise Stanton/Tom Maishman
Southampton Clinical Trials Unit
Southampton General Hospital

Professor Gareth Griffiths
Southampton Clinical Trials Unit
Southampton General Hospital

Patient Representative

Richard Stephens

TABLE OF CONTENTS

1 LIST OF ABBREVIATIONS 8
1.1 KEYWORDS 9
2 TRIAL SYNOPSIS 10
3 SCHEDULE OF OBSERVATIONS AND PROCEDURES 16
3.1 REGISTRATION AND CYCLE 1 16
3.2 FOLLOWING RANDOMISATION 18
3.2.1 Arm A: R-CHOP 18
3.2.2 Arm B: RB-CHOP 19
3.3 SCHEDULE OF FOLLOW-UP VISITS (BOTH ARMS) 20
4 INTRODUCTION 21
4.1 DISEASE BACKGROUND 21
4.2 BACKGROUND FOR STUDY 22
5 TRIAL OBJECTIVES 24
6 STUDY DESIGN 25
6.1 OVERVIEW OF STUDY DESIGN 25
6.2 STRUCTURE OF PATIENT ALLOCATION 26
6.3 NUMBER OF PATIENTS/ASSIGNMENT TO TREATMENT ARMS 27
6.4 DISCONTINUATION OF TRIAL TREATMENT 27
6.5 DISEASE PROGRESSION 27
6.6 END OF STUDY 27
6.7 CENTRE SELECTION 28
7 TRIAL OUTCOME MEASURES 28
7.1 PRIMARY OUTCOME MEASURE: 28
7.2 SECONDARY OUTCOME MEASURES: 28
8 SELECTION AND ENROLMENT OF PATIENTS 29
8.1 SCREENING AND PRE-REGISTRATION / RANDOMISATION EVALUATIONS 29
8.1.1 Molecular Profiling Consent 29
8.1.2 Consent to Main Trial 29
8.2 TARGET POPULATION 30
8.3 INCLUSION CRITERIA 30
8.4 EXCLUSION CRITERIA 30
8.5 PATIENT TRANSFERS 31
9 REGISTRATION / RANDOMISATION PROCEDURES 31
9.1 REGISTRATION 32
9.2 RANDOMISATION 32
10 TREATMENTS 32
10.1 OVERVIEW 32
10.2 TREATMENT ARMS 33
10.2.1 All patients - first cycle 33
10.2.1.1 R-CHOP: cycle1 (Days 1-21) 33

10.3 TREATMENT ASSIGNMENT 33

10.4 ARM A: R-CHOP 34

10.5 ARM B: RB-CHOP 34

10.6 CONTINUATION OF STUDY THERAPY 34

10.7 SUPPORTIVE CARE 34

10.8 ADDITIONAL THERAPY FOR DLBCL 35

10.8.1 Central nervous system prophylaxsis 35

10.8.2 Radiotherapy 35

10.9 PET IMAGING 35

11 DOSE MODIFICATIONS FOR TOXICITY 36

11.1 R-CHOP 36

11.1.1 Haematological Toxicity 36

11.1.1.1 Neutropenia 36

11.1.1.2 Thrombocytopenia 36

11.1.2 Non Haematological toxicity 37

11.1.2.1 Modification for neuropathic pain or peripheral neuropathy 37

11.1.2.2 Other non-Haematological toxicity 37

11.2 RB-CHOP 37

11.2.1 Haematological Toxicity 37

11.2.1.1 Neutropenia 37

11.2.1.2 Thrombocytopenia 38

11.2.2 Non Haematological toxicity 38

11.2.2.1 Modification for neuropathic pain or peripheral neuropathy 38

11.2.2.2 Renal impairment 41

11.2.2.3 Other non-Haematological toxicity 41

11.3 INTERACTION WITH OTHER DRUGS 41

11.3.1 Patients receiving bortezomib 41

11.4 STUDY THERAPIES 41

11.5 ACCOUNTABILITY 41

11.6 CO-ENROLLMENT GUIDELINES 42

12 ASSESSMENT AND FOLLOW-UP OF PATIENTS 42

12.1 ONE MONTH AFTER THE FINAL DOSE OF THERAPY 42

12.2 SUBSEQUENT FOLLOW- UP VISITS 43

12.3 ASSESSMENTS FOR PATIENTS WITHDRAWN FROM TRIAL THERAPY 43

12.4 SAFETY ASSESSMENT 43

12.5 QUALITY OF LIFE ASSESSMENT 43

12.6 TRANSLATIONAL RESEARCH 43

12.7 PATIENTS LOST TO FOLLOW-UP 44

13 PHARMACOVIGILANCE 44

13.1 DEFINITIONS 44

13.2 CAUSALITY 45

13.3 REPORTING PROCEDURES 46

13.3.1 Pre-existing Conditions 46

13.3.2 Non serious AR/AEs 46

13.3.3 Serious Adverse Events and Reactions 46

13.3.4 Reporting Details 46

13.3.5 Follow Up and Post-study SAEs and AEs 47

13.3.6 SARs not requiring Immediate Reporting 47
13.3.7 Pregnancy 47

14 DEFINITION OF ENDPOINTS 47

14.1 PRIMARY ENDPOINT 47

14.2 SECONDARY ENDPOINTS 48
14.2.1 Overall survival 48
14.2.2 Event-free survival 48
14.2.3 Disease-free survival 48
14.2.4 Time to Progression 48
14.2.5 Response Duration 48
14.2.6 Response Evaluation 48
14.2.6.1 Complete response (CR) 48
14.2.6.2 Complete response, undocumented/unconfirmed (CRu) 49
14.2.6.3 Partial response (PR) 49
14.2.6.4 Stable disease (SD): 50
14.2.6.5 Relapsed and Progressive disease (PD) 50
14.2.6.6 Summary of response criteria 51
14.2.6.7 PET/CT Response Assessment 51
14.2.7 Toxicity 52
14.2.8 Quality of Life 52

14.3 DEFINITION OF END OF TRIAL 52

15 STATISTICS AND DATA ANALYSIS 52

15.1 EARLY STOPPING RULES 52

15.2 STUDY POPULATIONS 53

15.3 SAMPLE SIZE AND POWER CALCULATIONS 53
15.3.1 Revised Sample Size
15.3.2 Timing of Final Analysis

15.4 INITIAL PLAN OF ANALYSIS: 55

16 REGULATORY ISSUES 56

16.1 CLINICAL TRIAL AUTHORISATION 56

16.2 ETHICS APPROVAL 56

16.3 CONSENT 57

16.4 CONFIDENTIALITY 57

16.5 INDEMNITY 57

16.6 SPONSOR 57

16.7 FUNDING 57

16.8 AUDITS AND INSPECTIONS 57

17 CASE REPORT FORMS AND MONITORING 58

17.1 COMPLETION OF THE CRF 58

17.2 STUDY PERFORMANCE AND MONITORING 58

17.3 SOURCE DOCUMENT VERIFICATION 58

17.4 DATA MANAGEMENT 59

17.5 SAFETY REPORTS 59

17.6 RECRUITMENT 59

17.7 PROTOCOL VIOLATIONS/DEVIATIONS 59

17.8 STUDY REPORT 59
17.9 RECORD RETENTION (ARCHIVING) 59
18 TRIAL MANAGEMENT 60

19 PUBLICATION POLICY 60

20 REFERENCES 61

APPENDIX 1 COMMUNICATION AND RELATIONSHIP BETWEEN PARTIES 66

APPENDIX 2 ECOG PERFORMANCE STATUS 67

APPENDIX 3 INTERNATIONAL PROGNOSTIC INDEX 68

APPENDIX 4 COMMON TERMINOLOGY CRITERIA FOR ADVERSE EVENTS 69

APPENDIX 5 QUALITY OF LIFE QUESTIONNAIRES 67

APPENDIX 6 RANDOMISATION FLOW CHART GUIDE 74

APPENDIX 7 EXAMPLE LIST OF EXPECTED TOXICITIES 75

APPENDIX 8 CYP3A4 INHIBITORS AND INDUCERS 76

APPENDIX 9 DECLARATION OF HELSINKI 77

1 LIST OF ABBREVIATIONS

ABC	Activated B-cell
ADL	Activities of Daily Living
AE	Adverse Event
ALT	Alanine Aminotransferase
AR	Adverse Reaction
ARSAC	Administration of Radioactive Substances Advisory Committee
AST	Aspartate aminotransferase
BSA	Body Surface Area
CHOP	Cyclophosphamide, Doxorubicin, Vincristine and Prednisolone
CNS	Central Nervous System
CR	Complete Response
CRu	Complete Response undocumented/unconfirmed
CRF	Case Report Form
CT	Computer Tomography
CTA	Clinical Trial Authorisation
CTCAE	Common Terminology Criteria for Adverse Events
CVP	Cyclophosphamide, Vincristine and Prednisolone
DASL	cDNA-mediated Annealing, Selection, extension and Ligation
DFS	Disease Free Survival
DLBCL	Diffuse Large B-cell Lymphoma
DMEC	Data Monitoring and Ethics Committee
ECG	Electrocardiogram
ECOG	Eastern COoperative Group
EFS	Event Free Survival
EORTC	European Organisation for Research and Treatment of Cancer
EPOCH-R	Etoposide, Prednisolone, Vincristine, Cyclophosphamide and Doxorubicin
FBC	Full Blood Count
FDA	Food and Drug Administration
FFPE	Formalin Fixed Paraffin Embedded
FL	Follicular Lymphoma
GCB	Germinal Centre B-cell
GCP	Good Clinical Practice
GCSF	Granulocyte Colony Stimulating Factor
HepBsAg	Hepatitis B Surface Antigen
HACA	Human Anti-Chimeric Antibody
HAMA	Human Anti- Mouse Antibody
HBV	Hepatitis B Virus
HCV	Hepatitis C Virus
HIV	Human Immunodeficiency Virus
HMDS	Haematological Malignancies Diagnostic Service
HR	Hazard Ratio
IB	Investigator’s Brochure
ICH GCP	International Conference on Harmonisation of Good Clinical Practice
IDMC	Independent Data Monitoring Committee
IHC	immunohistochemistry
IHP	International Harmonisation Project
IMP	Investigational Medicinal Product
IPI	International Prognostic Index
IV	Intravenous
LDH	Lactate Dehydrogenase

MHRA	Medicines and Healthcare products Regulatory Authority
mRNA	messenger Ribonucleic Acid
NCI	National Cancer Institute
NCRI	National Cancer Research Institute
NHL	Non-Hodgkin's lymphoma
NRES	National Research Ethics Service
od	once daily
ORR	Overall Response Rate
OS	Overall Survival
PD	Progressive Disease
PET	Positron Emission Tomography
PFS	Progression-Free Survival
PI	Principal Investigator
po	By mouth
PR	Partial Response
QoL	Quality of Life
R	Rituximab
RB-CHOP	Rituximab with Cyclophosphamide, Doxorubicin, Vincristine, Prednisolone and Bortezomib
R-CHOP	Rituximab with Cyclophosphamide, Doxorubicin, Vincristine and Prednisolone
RD	Response Duration
REC	Research Ethics Committee
RNA	Ribonucleic Acid
RR	Response Rate
SAE	Serious Adverse Event
SAKK	Schweizerische Arbeitsgemeinschaft für Klinische Krebsforschung/ Swiss Group for Clinical Cancer Research
SAR	Serious Adverse Reaction
SD	Stable Disease
SmPC	Summary of Product Characteristics
SPC	Specific Product Characteristics
SPD	Sum of the Product of the Diameters
SSA	Site Specific Assessment
SUSAR	Suspected Unexpected Serious Adverse Reaction
TMG	Trial Management Group
tRNA	transfer Ribonucleic Acid
TSC	Trial Steering Committee
TTP	Time To Progression
UAR	Unexpected Adverse Reaction
ULN	Upper Limit of Normal
SCTU	Southampton Clinical Trials Unit
WHO	World Health Organisation

1.1 KEYWORDS

Diffuse large B-cell lymphoma
Bortezomib
R-CHOP
Molecular profiling
Chemotherapy

2 TRIAL SYNOPSIS

**Title:**	REMoDL-B: A Randomised Evaluation of Molecular guided therapy for Diffuse Large B-cell Lymphoma with Bortezomib
**Sponsor:**	University Hospital Southampton NHS Foundation Trust
**Sponsor Ref Number:**	RHMCAN0749
**Funder:**	CTAAC endorsed and support by an unrestricted educational grant from Janssen-Cilag
**Trial Phase:**	Phase III adaptive design
**Indication:**	Previously untreated CD20 positive diffuse large B-cell lymphoma (DLBCL) requiring full course chemotherapy
**Primary Objective:**	To demonstrate superior clinical efficacy, as measured by Progression-free survival (PFS), of bortezomib in combination with rituximab and CHOP (RB-CHOP) versus R-CHOP alone for the treatment of previously untreated patients with DLBCL.<br><br>To assess whether the molecular phenotype, either activated B-cell (ABC) like or germinal centre B-cell (GCB) like, of DLBCL determines the benefit from the addition of bortezomib.
**Secondary Objective:**	To compare overall survival (OS) between both treatment and molecular groups<br><br>To compare event free survival (EFS) between both treatment and molecular groups<br><br>To compare disease free survival (DFS) between both treatment and molecular groups<br><br>To compare time to progression (TTP) between both treatment and molecular groups<br><br>To compare response duration (RD) between both treatment and molecular groups<br><br>To compare overall response rate (ORR) and complete response rate (CR) between both treatment groups<br><br>To assess differences in toxicity between assigned treatments<br><br>To assess quality of life and chemotherapy induced peripheral neuropathy reported by patients across the two different induction treatments<br><br>To demonstrate that accurate molecular profiling may be conducted in real-time and relate this to immunohistochemical algorithms.<br><br>To perform exploratory analysis comparing aberrations in the NF-κB pathway to clinical outcomes by treatment groups
**Rationale:**	Retrospective molecular profiling of untreated DLBCL samples has recognised distinct sub-classifications of this disease, each with

	unique biological features and clinical outcomes when treated with CHOP or R-CHOP chemotherapy. Constitutive activation of the NF-κB signalling pathway is observed in ABC-DLBCL (and some GCB-DLBCL). The former has a poor prognosis; it is postulated that inhibition of this pathway, through the addition of bortezomib, a proteasome inhibitor, may improve outcomes of patients with DLBCL.
**Trial Design:**	Stratified open-label multicentre randomised Phase III. A novel adaptive trial design will be employed with 2 interim analysis to explore safety and efficacy in the GCB arm patients treated with RB- CHOP. Modified to randomised Phase II in ABC patients only if GCB arm closed.
**Sample size :**	Up to 1132 patients. (765 should GCB strata close).
**Inclusion Criteria:**	• Histologically confirmed DLBCL, expressing CD20. Sufficient diagnostic material should be available to forward to HMDS for gene expression profiling and central pathology review<br>• Measurable disease of at least 15mm<br>• Not previously treated for lymphoma and fit enough to receive combination chemoimmunotherapy with curative intent<br>• Stage I_{AX} (bulk defined as lymph node diameter >10cm) to stage IV disease requiring full course chemotherapy<br>• ECOG performance status 0-2<br>• Aged 18 or above<br>• Adequate bone marrow function with platelets $>100x10^9/L$; neutrophils $>1.0x10^9/L$ at study entry, unless lower figures are attributable to lymphoma<br>• Serum creatinine <150μmol/L, serum bilirubin <35μmol/L and transaminases <2.5x upper limit of normal at the time of study entry, unless attributable to lymphoma<br>• Cardiac function sufficient to tolerate $300mg/m^2$ of doxorubicin. A pre-treatment echocardiogram is not mandated, but recommended in patients considered at higher risk of anthracycline cardiotoxicity<br>• No concurrent uncontrolled medical condition<br>• Life expectancy >3 months<br>• Adequate contraceptive precautions for all patients of child bearing potential<br>• A negative serum pregnancy test for females of child bearing potential or those <2 years after the onset of the menopause<br>• Patients will have provided written informed consent

**Exclusion Criteria:**	• Previous history of treated or untreated indolent lymphoma. However newly diagnosed patients with DLBCL who are found to also have small cell infiltration of the bone marrow or other diagnostic material (discordant lymphoma) will be eligible<br>• Diagnosis of primary mediastinal lymphoma<br>• Uncontrolled systemic infection<br>• History of cardiac failure or uncontrolled angina<br>• Clinical CNS involvement<br>• Serological positivity for Hepatitis C, B or HIV (standard of care testing)<br>• Serious medical or psychiatric illness likely to affect participation or that may compromise the ability to give informed consent<br>• Pregnant or nursing females<br>• Active malignancy other than fully excised squamous or basal cell carcinoma of the skin or carcinoma in situ of the cervix in the preceding 5 years<br>• History of allergic reaction to substances containing boron or mannitol<br>• Patient unwilling to abstain from green tea and preparations made from green tea as bortezomib may interact with these

**Investigational Products:**	Bortezomib, rituximab, cyclophosphamide, doxorubicin, vincristine, prednisolone
**Dosage Regimen / Duration of Treatment:**	All study patients will receive 1 cycle of conventional R-CHOP chemotherapy on a standard 21 day schedule.<br><br>**<u>R CHOP</u>**<br>*Day 1*<br>Rituximab 375mg/m^2 IV<br>Cyclophosphamide 750mg/m^2 IV<br>Doxorubicin 50mg/m^2 IV<br>Vincristine 1.4mg/m^2 (max 2mg) IV<br>*Days 1-5*<br>Prednisolone 100mg od po<br><br>In parallel, formalin fixed paraffin embedded diagnostic material will be forwarded to the HMDS in Leeds for molecular sub-typing by gene expression profiling. This will be determined in real-time and the result available for randomisation prior to the second cycle of therapy. The randomisation between R-CHOP and R- CHOP+bortezomib will be stratified by ABC and GCB sub-types.<br><br>**<u>Arm A: R-CHOP</u> (control arm)**<br>Patients will continue on to receive a further 5 cycles of conventional R-CHOP chemotherapy on a 21 day schedule<br><br>**<u>Arm B: RB-CHOP</u>** (experimental arm)<br>Patients in this arm will receive 5 cycles of R-CHOP with bortezomib according to the schedule below<br><br>*Day 1*<br>Bortezomib 1.6 mg/m^2 SC<br>Rituximab 375mg/m^2 IV<br>Cyclophosphamide 750mg/m^2 IV<br>Doxorubicin 50mg/m^2 IV<br>Vincristine 1.4mg/m^2 (max 2mg) IV<br>*Days 1-5*<br>Prednisolone 100mg od po<br>*Day 8*<br>Bortezomib 1.6 mg/m^2 SC
**Concomitant Therapy:**	According to local policy
**Primary Trial Endpoints:**	Progression-free survival
**Secondary Trial Endpoints:**	Overall survival<br>Event-free survival<br>Disease-free survival<br>Complete and overall response rates

	Evaluation of toxicity (according to CTCAE version 4.0)<br>Quality of life and assessment of peripheral neuropathy (employing the EORTC QLQ-C30 tool with ClPN20 module for assessment of chemotherapy induced peripheral neuropathy)
**Total Number of Sites**	>80 UK sites and 15 Swiss Sites

Trial Outline:

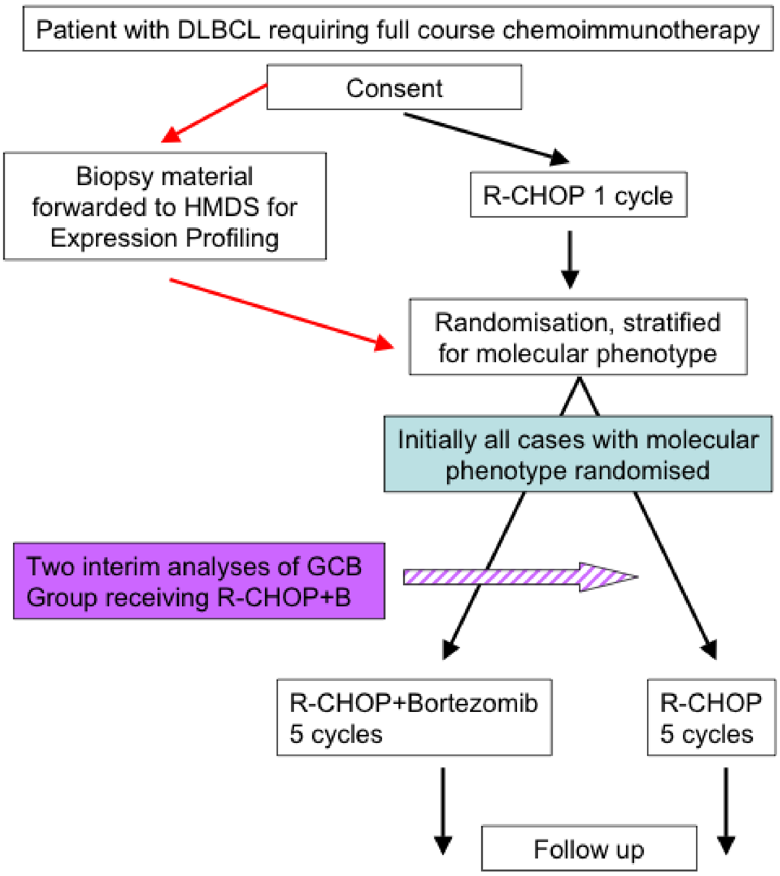

3 SCHEDULE OF OBSERVATIONS AND PROCEDURES

3.1 REGISTRATION AND CYCLE 1

**Visit:**	**Screening**[a]			**Cycle 1**
**Week(s):**				**1**
**Day(s):**	**Within 90 days of treatment**	**Within 42 days of treatment**	**Within 14 days of treatment**	**R-CHOP**
Informed Consent		**X**		
Inclusion /Exclusion Criteria			**X**	
Medical History			**X**	
Physical Exam			**X**	
Vital Signs [b]			**X**	**X**
ECOG performance status			**X**	**X**
CT[c]		**X**		
Bone marrow biopsy [d]	**X**			
Biochemistry: renal and liver function [e]			**X**	
Biochemistry: Additional baseline panel [f]			**X**	
Haematology [g]			**X**	
Hepatitis B and C serology [h]		X		
Pregnancy test [i]			**X**	
Electrocardiogram[j]		**X**		
Cerebrospinal fluid examination [k]			**X**	
EORTC QLQ-C30 + CIPN20 questionnaire				**X**
Tumour material[l]		**X**		
Peripheral blood for FACS analysis and storage (to HMDS) [m]			**X**	
R-CHOP				**X**
Concomitant Medications			**X**	**X**
Adverse Events			**X**	**X**

[a] Screening investigations to performed within 14 days of starting study medication with the exception of informed consent, CT, Electrocardiogram & Bone marrow biopsy
[b] Blood pressure pulse and temperature. Assessment to be performed predose and as per local practice during rituximab infusion
[c] Contrast enhanced CT of chest, abdomen and pelvis (neck if indicated) should be carried out within 42 days of planned treatment. PET/CT hybrid scanners may be used to acquire the required CT images only if the CT produced by the scanner is of diagnostic quality and includes the use of intravenous (IV) contrast.
[d] Bone marrow aspirate and trephine biopsy (single site with adequate trephine) (within 90 days of first treatment). Sites may register patients without these results, but must give them to the SCTU before the patient is randomised
[e] Serum chemistry to include sodium, potassium, urea, creatinine, bilirubin, alanine or aspartate transaminase, alkaline phosphatise and albumin. To be taken within 72 hours of administration of each cycle (except cycle 1)
[f] Additional serum chemistry to be performed at baseline: LDH, calcium, phosphate, β2 microglobulin and uric acid at baseline only
[g] Full blood count to include haemoglobin, white cell count, absolute neutrophil count, lymphocytes and platelets. To be taken within 72 hours of chemotherapy administration on each cycle
[h] As per standard of care, sites should have hepatitis results available prior to initiation of immunochemotherapy. In urgent cases, sites may register patients without Hepatitis results, but these must be available to the SCTU before the patient is randomised.
[i] Only required in females of child bearing potential
[j] A 12 lead ECG should be performed on all patients. In addition, an Echocardiogram or MUGA should be performed for patients over the age of 70, and known diabetics over the age of 60. It should also be performed at baseline in all patients, with a history of cardiac disease, hypertension or an abnormal resting ECG. Patients must have a left ventricular ejection fraction of ≥50% and should be considered suitable to receive 300mg/m^2 doxorubicin
[k] Cerebrospinal fluid examination should be performed if clinically indicated or lymphomatous involvement of bone marrow, peripheral blood, nasal/paranasal sinuses or testis. CNS prophylaxis may be given according to local policy
[l] Diagnostic tumour block to be forwarded immediately upon obtaining Molecular Profiling informed consent to HMDS, Leeds, according to study procedure outlined in Investigator Site File (ISF)
[m] 2 x 4.5 ml EDTA samples required for FACS and storage, according to study procedure outlined in ISF

3.2 FOLLOWING RANDOMISATION

3.2.1 Arm A: R-CHOP

**Visit:**	**Cycle 2**	**Cycle 3**	**Cycle 4**	**Cycle 5**	**Cycle 6**	**End of treatment**
**Week(s):**	**4**	**7**	**10**	**13**	**16**	**20 (month 1)**
Physical Exam	**X**	**X**	**X**	**X**	**X**	**X**
Vital Signs[n]	**X**	**X**	**X**	**X**	**X**	**X**
ECOG performance status	**X**	**X**	**X**	**X**	**X**	**X**
CT[o]						**X**
Bone marrow biopsy						**(X[p])**
Biochemistry: renal and liver function[q]	**X**	**X**	**X**	**X**	**X**	**X**
Haematology[r]	**X**	**X**	**X**	**X**	**X**	**X**
EORTC QLQ-C30 + CIPN20	**X**	**X**	**X**	**X**	**X**	**X**
R-CHOP	**X**	**X**	**X**	**X**	**X**	
Concomitant medications	**X**	**X**	**X**	**X**	**X**	**X**
Adverse Events	**X**	**X**	**X**	**X**	**X**	**X**

[n] Blood pressure pulse and temperature. Assessment to be performed pre-dose and as per local practice during rituximab infusion
[o] Contrast enhanced CT of chest, abdomen and pelvis (neck if indicated) should be carried out one month after the end of treatment. PET/CT hybrid scanners may be used to acquire the required CT images only if the CT produced by the scanner is of diagnostic quality and includes the use of intravenous (IV) contrast.
[p] Bone marrow biopsy to be repeated at the end of treatment if initially involved (to confirm CR)
[q] Serum chemistry to include sodium, potassium, urea, creatinine, bilirubin, alanine or aspartate transaminase, alkaline phosphatise and albumin. To be taken within 72 hours of administration of each cycle
[r] Full blood count to include haemoglobin, white cell count, absolute neutrophil count, lymphocytes and platelets. To be taken within 72 hours of chemotherapy administration on each cycle

3.2.2 Arm B: RB-CHOP

Visit	Cycle 2		Cycle 3		Cycle 4		Cycle 5		Cycle 6		End of treatment
**Week(s)**	**4**		**7**		**10**		**13**		**16**		**20** (month 1)
**Day(s)**	**1**	**8**	**1**	**8**	**1**	**8**	**1**	**8**	**1**	**8**	
Physical Exam	X		X		X		X		X		X
Vital Signs [s]	X	X	X	X	X	X	X	X	X	X	X
ECOG performance status	X	X	X	X	X	X	X	X	X	X	X
CT [t]											X
Bone marrow biopsy											(X[u])
Biochemistry: renal and liver function [v]	X		X		X		X		X		X
Haematology [w]	X	X	X	X	X	X	X	X	X	X	X
EORTC QLQ-C30 + CIPN20 questionnaire	X		X		X		X		X		X
R-CHOP	X		X		X		X		X		
Bortezomib	X	X	X	X	X	X	X	X	X	X	
Concomitant medications	X	X	X	X	X	X	X	X	X	X	X
Adverse Events	X	X	X	X	X	X	X	X	X	X	X

[s] Blood pressure pulse and temperature. Assessment to be performed pre-dose and as per local practice during rituximab infusion
[t] Contrast enhanced CT of chest, abdomen and pelvis (neck if indicated) one month after the end of treatment
[u] Bone marrow biopsy to be repeated at the end of treatment if initially involved (to confirm CR)
[v] Serum chemistry to include sodium, potassium, urea, creatinine, bilirubin, alanine or aspartate transaminase, alkaline phosphatase and albumin. To be taken within 72 hours of administration of each cycle (except cycle 1)
[w] Full blood count to include haemoglobin, white cell count, absolute neutrophil count, lymphocytes and platelets. To be taken within 72 hours of chemotherapy administration on each cycle

3.3 SCHEDULE OF FOLLOW-UP VISITS (BOTH ARMS)

**Months following last chemotherapy/radiotherapy[y]:**	**3**	**6**	**9**	**12**	**18**	**24**	**30**	**36**	**42**	**48**	**54**	**60**
Physical Exam	**X**	**X**	**X**	**X**	**X**	**X**	**X**	**X**	**X**	**X**	**X**	**X**
ECOG performance status	**X**	**X**	**X**	**X**	**X**	**X**	**X**	**X**	**X**	**X**	**X**	**X**
Haematology	**X**	**X**	**X**	**X**	**X**	**X**	**X**	**X**	**X**	**X**	**X**	**X**
LDH	**X**	**X**	**X**	**X**	**X**	**X**	**X**	**X**	**X**	**X**	**X**	**X**
CT[X]				**X**								
EORTC QLQ-C30 + CIPN20 questionnaire	**X**	**X**	**X**	**X**	**X**	**X**		**X**		**X**		**X**
Adverse Events[z]	**X**	**X**	**X**	**X**	**X**	**X**	**X**	**X**	**X**	**X**	**X**	**X**

[X] A contrast enhanced CT scan of the chest, abdomen and pelvis (neck if clinically indicated) will be performed at 12 months following completion of protocol specified therapy

[y] Including the two extra cycles of Rituximab

[Z] Only significant AEs associated with the study should be reported during the follow up period

4 INTRODUCTION

4.1 DISEASE BACKGROUND

More than 10 000 new cases of non-Hodgkin's lymphoma (NHL) are diagnosed in the UK with approximately 4,500 attributable deaths each year (Cancer Research UK accessed August 2009). In the Western World, diffuse large B-cell lymphoma (DLBCL) comprises one third of all adult NHL cases (The Non-Hodgkin's Lymphoma Classification Project 1997), accounting for an annual incidence in the UK of approximately 3000 patients.

For many years CHOP (cyclophosphamide, vincristine, doxorubicin and prednisolone) was the standard of care for DLBCL although overall survival was disappointing, with only about half the patients cured (Fisher, *et al* 1993). The concurrent use of the anti CD20 monoclonal antibody rituximab resulted in a major paradigm shift with higher response rates, longer event free survival and improved overall survival, so that R-CHOP is now accepted as the international standard, with cure rates around 75%(Coiffier, *et al* 2002, Habermann, *et al* 2006, Pfreundschuh, *et al* 2008, Pfreundschuh, *et al* 2006, Sehn, *et al* 2005).

Despite this improvement, a substantial number of patients still have disease which either fails to respond to initial therapy, or relapses after initial remission. The majority of these will die as a result of their disease, because the success of salvage treatment appears to be limited after prior rituximab treatment (Gisselbrecht, *et al* 2009). There is therefore a need to further improve the efficacy of first line treatment.

One approach has been to increase the dose density of CHOP, giving it every 14 days with growth factor support instead of every 21 days. The German NHL-B2 trial conducted in elderly patients with DLBCL, but not using rituximab, found that this approach was superior with a 5 year event free survival of 44% in the CHOP-14 arm and 33% in the CHOP-21 arm (Pfreundschuh, *et al* 2004a), although no such benefit could be identified in younger patients studied in the companion NHL-B1 study (Pfreundschuh, *et al* 2004b). The benefit of adding rituximab to the R-CHOP 14 schedule was demonstrated in the RICOVER-60 trial (Pfreundschuh, *et al* 2008). This also demonstrated that there was no benefit of 8 cycles over 6 cycles of immunochemotherapy. The UK NCRI study of R-CHOP 14 vs 21 included 1080 patients of all ages and closed to recruitment in November 2008(Cunningham, *et al* 2009). Preliminary results have been reported which indicate that the more intensive schedule is not associated with an improvement in the complete response rate, and there have not yet been sufficient events for an analysis of its primary endpoint of progression free survival. Results from a planned interim analysis of the GELA LNH03-6B study of R-CHOP 14 against R-CHOP 21 in older patients have recently been reported (Delarue, *et al* 2009). These demonstrate no significant difference in event free survival at 2 years (48% and 61% respectively).

An alternative approach is to consider adding more agents. A number of approaches with conventional cytotoxics have been tried, but these have not proven to improve upon CHOP in randomised studies(Fisher, *et al* 1993). With increased knowledge of the biology of DLBCL and the availability of new targeted therapeutics, there is an opportunity to study more specific interventions based upon the pathogenetic abnormalities in the lymphoma cells.

4.2 BACKGROUND FOR STUDY

Gene expression profiling of fresh frozen material has provided new insights into the biology of DLBCL. In patients treated with CHOP or CHOP like chemotherapy, unsupervised hierarchical clustering identified two distinct subgroups of the disease; one with a phenotype similar to germinal centre derived B-cells (GCB like) and one with a phenotype similar to activated peripheral B-cells (ABC like)(Alizadeh, *et al* 2000). These molecular subgroups had distinct clinical outcomes. The initial observations were extended to a larger series of tumours by the Leukaemia and Lymphoma Molecular Profiling Project (Rosenwald, *et al* 2002) and the molecular model subsequently refined to a discriminator based upon the expression of just 27 genes(Rimsza, *et al* 2009). With the advent of R-CHOP chemotherapy, this molecular classification was found to be robust, and the inferior prognosis of the ABC-like lymphomas remained inferior with 3 year progression free survival of the ABC group 40% compared to 75% in the GCB group (Lenz, *et al* 2008b). It is in the ABC group that a new approach to treatment appears to be most urgently needed.

Several oncogenic mechanisms distinguish the two sub-groups(Lenz, *et al* 2008c), in particular the observation that constitutive activation of the nuclear factor-κB (NF-κB) signalling pathway appears central to cell survival in ABC-like lymphomas(Davis, *et al* 2001). These tumours rely upon the CARD11/MALT1/BCL10 signalling complex to activate the central kinase in the NF-κB pathway, IκB kinase(Lenz, *et al* 2008a, Ngo, *et al* 2006). More than half of ABC-DLBCL carry somatic mutations in multiple genes, including negative (A20) and positive (CARD11, TRAF2, TRAF5, MAP3K7 and TNFRSF11A) regulators of NF-kB(Compagno, *et al* 2009). Of these, the A20 gene, which encodes aubiquitin-modifying enzyme involved in termination of NF-kB responses, is most commonly affected, with one third of patients displaying biallelic inactivation by mutations and/or deletions. Functional characterisation of GCB-like DLBCL cell lines has suggested that the NF-κB pathway may also be activated in a small subset of this group(Gilmore, *et al* 2004). The induction of the NF-κB pathway appears to suppress the apoptotic effect of cytotoxic chemotherapy (Packham 2008) and this may contribute to the observed differences in outcome between the biological subtypes of DLBCL.

Attempts have been made to simplify molecular sub-classification of DLBCL by immunohistochemistry (IHC) methodologies (Choi, *et al* 2009, Hans, *et al* 2004). Although these have the attraction of being readily applied to routine diagnostic material, there is a major problem of poor reproducibility between laboratories and pathologists, even when expert haematopathologists are used, with a lack of concordance using semiquantiative techniques(de Jong, *et al* 2007). None of the IHC algorithms developed to date appears to give robust prognostic information, in contrast to the gene expression studies which have distinguished groups of markedly differing prognosis treated with either CHOP or R-CHOP.

Technological evolution has now enabled the application of gene expression profiling on formalin fixed paraffin embedded (FFPE) tumour material where mRNA may be partly degraded. The use of FFPE material to distinguish the molecular phenotype of DLBCL has already been validated by other investigators(Rimsza, *et al* 2009).

The Illumina cDNA-mediated annealing, selection, extension and ligation (DASL) array allows analysis of 24 000 probe sets, with excellent correlation to fresh frozen material (r^2>0.98) and with a low starting tRNA of only 10ng. At HMDS the ABC/GCB discriminator genes perform well on this platform and confirm the existence of the two groups, recapitulating the model previously described. The Bayesian algorithm reported by Wright et al.(Wright, *et al* 2003) has been reproduced and performs well at determining the molecular subgroup. This technology is sensitive, so even relatively small biopsies may be examined, although a failure rate of 15% has

been recorded due to insufficient RNA extraction (mostly in small biopsies). The molecular classification of DLBCL may be obtained from FFPE in 4 days; the technology is readily scaleable and is applicable for the determination of ABC or GCB subtype in this study.

Bortezomib is a proteosome inhibitor whose effects are, at least in part, mediated through inhibition of the NF-κB pathway (Nencioni, *et al* 2007). Its clinical utility in a number of haematological malignancies has been demonstrated(Goy, *et al* 2005, Strauss, *et al* 2006) and it may provide a suitable intervention, in addition to R-CHOP, to improve outcomes in DLBCL, particularly the ABC subgroup. There is some preliminary evidence to suggest that this may be the case. Dunleavy *et al* hypothesised that the addition of bortezomib to dose adjusted EPOCH-R (etoposide, prednisolone, vincristine, cyclophosphamide and doxorubicin) chemotherapy would improve the outcome of ABC DLBCL by inhibition of NF-κB in recurrent disease(Dunleavy, *et al* 2009). This was explored in a single institution study at the NCI: As a single agent during a short pre-phase bortezomib demonstrated minimal activity, but in combination resulted in a median overall survival of 10.8 months in recurrent ABC DLBCL compared to 3.4 months in GCB DLBCL (P=0.0026). The authors concluded that bortezomib enhanced the activity of chemotherapy in the ABC, but not the GCB DLBCL.

There is accumulating evidence that bortezomib may safely be given with R-CHOP chemotherapy, allaying concerns about the potential for additive peripheral neuropathy. The GELA group conducted a randomised Phase II trial of R-CHOP for DLBCL with the addition of bortezomib on two schedules, either Days 1, 4, 8, and 11 (Arm A) or Days 1 and 8 (Arm B)(Mounier, *et al* 2007 (abstr.)). Initially bortezomib was given at a dose of 1 mg/m^2 in Arm A and 1.3 mg/m^2 in Arm B. In the second step doses were increased to 1.3 mg/m^2 and 1.6 mg/m^2 respectively. Eighty-four percent of patients achieved a CR/CRu. Neurological toxicity occurred in 21 patients (43%) and was more frequent in the twice weekly schedule grade 2 in 11 patients (7 patients in Step 2) and grade 3 in 10 patients (9 patients in Step 2). The authors concluded that lower doses should be used in a weekly schedule. Leonard et al have presented a Phase I-II trial of dose-escalated bortezomib with standard R-CHOP in patients with DLBCL (Leonard, *et al* 2007). Bortezomib was given at a dose of 0.7 mg/m^2, 1.0 mg/m^2, or 1.3 mg/m^2 on Days 1 and 4 of each cycle. Thirty-six patients in that study were evaluable: The objective response rate was 100%, and the CR/CRu rate was 75% The 2-year progression- free survival was estimated at 72%. Peripheral neuropathy occurred in 22 patients (55%) and was grade 1 in 45% .

As initial therapy for mantle cell lymphoma, Ruan et al. administered R-CHOP with bortezomib 1.3 mg/m^2 on Days 1 and 4; although 56% of patients reported neuropathy, 80% of these were grade 1 (Ruan, *et al* 2009). Sinha et al reported on 17 patients with indolent lymphoma treated with R-CHOP and bortezomib between 1.0mg/m^2 and 1.6 mg/m^2 on Days 1 and 8 of a 21 day cycle (Sinha, *et al* 2009). The dose of vincristine was capped at 1.5 mg (normal dose 2mg). The overall response rate to therapy was 100% and only 1 patient experienced grade 3 neuropathy. Using a quality of life tool to assess neuropathy, 88% of patients reported little or no neuropathy. In addition, Kim et al have administered full dose CHOP-14 with bortezomib 1.6mg/m^2 on Days 1 and 4 to 35 patients with DLBCL in a phase II study (Kim, *et al* 2009). The authors reported a 92% complete remission rate and an 'acceptable' rate of neurological toxicity. In a large phase II study from Canada of bortezomib (1.3 mg/m^2 Day 1 and 8) plus R- CVP (cyclophosphamide, vincristine, and prednisolone), grade 3 neuropathy occurred in only 6 of 95 patients and was largely reversible. Dose reductions were necessary in 16% of patients (Sehn, *et al* 2009). In relapsed mantle cell lymphoma, an NCRI study is currently using bortezomib 1.6 mg/m^2 on Days 1 and 8 in combination with full dose CHOP. Treatment is well tolerated with a minimal incidence of neuropathy (personal communication: Dr Simon Rule).

This information has been used to derive a dose level of bortezomib 1.6 mg/m^2 given on Days 1 and 8 in combination with R-CHOP, which has the advantages of exposure to bortezomib at a biologically active dose, proven low risk of neurotoxicity and convenience of administration. Recent emerging data reported that the use s/c bortezomib has reported similar therapeutic efficacy to IV bortezomib but with a lower incidence of reported level 3 of any peripheral neuropathy toxicity. Bortezomib may also be administered subcutaneously at a dose of 1.6mg/^{2} which pharmacologically delivers with similar therapeutic efficacy but with a lower incidence of reported level 3 peripheral neuropathy toxicity. (38% s/c vs 53% IV). It is recommended to use the s/c route in view of these findings and because of greater convenience for patients and treating physicians. Appropriate dose de-escalation is built into the protocol in case of emergent neurotoxicity.

5 TRIAL OBJECTIVES

The primary of objectives of this study are:

To demonstrate superior clinical efficacy, as measured by progression-free survival (PFS), of bortezomib in combination with rituximab and CHOP (RB-CHOP) versus R- CHOP alone for the treatment of previously untreated patients with DLBCL.

To assess whether the molecular phenotype, either activated B-cell (ABC) like or germinal centre B-cell (GCB) like, of DLBCL determines the benefit from the addition of bortezomib.

Secondary objectives are:

To compare overall survival (OS) between both treatment and molecular groups.

To compare event free survival (EFS) between both treatment and molecular groups.

To compare disease free survival (DFS) between both treatment and molecular groups. To

compare time to progression (TTP) between both treatment and molecular groups. To compare

response duration (RD) between both treatment and molecular groups.

To compare overall response rate (ORR) and complete response rate (CR) between both treatment groups.

To assess differences in toxicity between assigned treatments.

To assess quality of life and chemotherapy induced peripheral neuropathy reported by patients across the two different induction treatments.

To demonstrate that accurate molecular profiling may be conducted in real-time and relate these to immunohistochemical algorithms.

To perform exploratory analysis comparing aberrations in the NF-κB pathway to clinical outcomes by treatment groups.

6 STUDY DESIGN

6.1 OVERVIEW OF STUDY DESIGN

A multicentre open-label adaptive randomised Phase III trial with 2 treatment arms and stratification according to ABC or GCB DLBCL and IPI.

Following informed consent all patients will receive 1 cycle of conventional R-CHOP chemotherapy. At the same time the FFPE diagnostic pathology block will be forwarded to the HMDS in Leeds for molecular profiling and assignment of subtype to GCB, ABC, unclassified or failed RNA extraction. The delivery of the first cycle of R-CHOP will allow a sufficient interval for real time determination of molecular type. Patients whose biopsies yield sufficient RNA and are molecularly typed will be randomised 1:1 between R-CHOP for a further 5 cycles (Arm A) or R-CHOP + bortezomib (Arm B) for 5 cycles. The randomisation will be stratified between ABC and GCB to ensure comparison between groups is robust, and will also be stratified by conventional prognostic factors using the International Prognostic index (Appendix 3). Patients whose biopsy material is molecularly unclassified will be randomised with the ABC group, based upon predicted clinical behaviour (their biology is unknown), but randomisation in the ABC stratum will not be halted until the full number of 'confirmed' ABC DLBCL are randomised. In the case of failed RNA extraction the patient will not be randomised, and will receive conventional R-CHOP and followed, with assessments, according to arm A of the trial. The local Investigator will not be informed of the results of the molecular phenotyping.

In order to avoid delays in acquisition of tissue blocks for molecular profiling at HMDS, a two part consent process has been built into the design of REMoDL-B. Patients may consent for their tumour material to be sent for analysis whilst they consider their willingness to participate in the clinical trial.

The biology of the molecular subgroups predicts that the ABC DLBCL will benefit from the addition of bortezomib, although efficacy in GCB DLBCL cannot be excluded *a priori.* Therefore a number of interim analyses with stopping rules have been integrated into this adaptive study design to assess safety and futility in the GCB arm.

The randomisation, and initiation of bortezomib for half the patients, is timed to occur after the first cycle to ensure that all randomised patients have a successful profile obtained, and to allow only ABC patients to be randomised if the interim analyses indicate that the GCB arm should close. This has the additional advantage that patients will not be administered the investigational agent during the first cycle when they are often most unwell from the lymphoma.

Consideration has been given to randomising patients with ABC DLBCL only, but this approach has not been followed for two reasons: (a) the amount of information on the prognostic significance of gene expression profiles is still not considered great enough to justify a whole trial based entirely upon this. The only study in patients treated with R-CHOP (as opposed to CHOP) is a retrospective analysis (Lenz, *et al* 2008b), and the consensus among UK lymphoma researchers is that REMoDL-B needs to include a demonstration of prospective prognostic value for the classification. (b) The precise mechanism of action of bortezomib is still not fully defined, and there remains a possibility that therapeutic benefit could accrue from its use in patients other than those with active NF-kB.

6.2 STRUCTURE OF PATIENT ALLOCATION

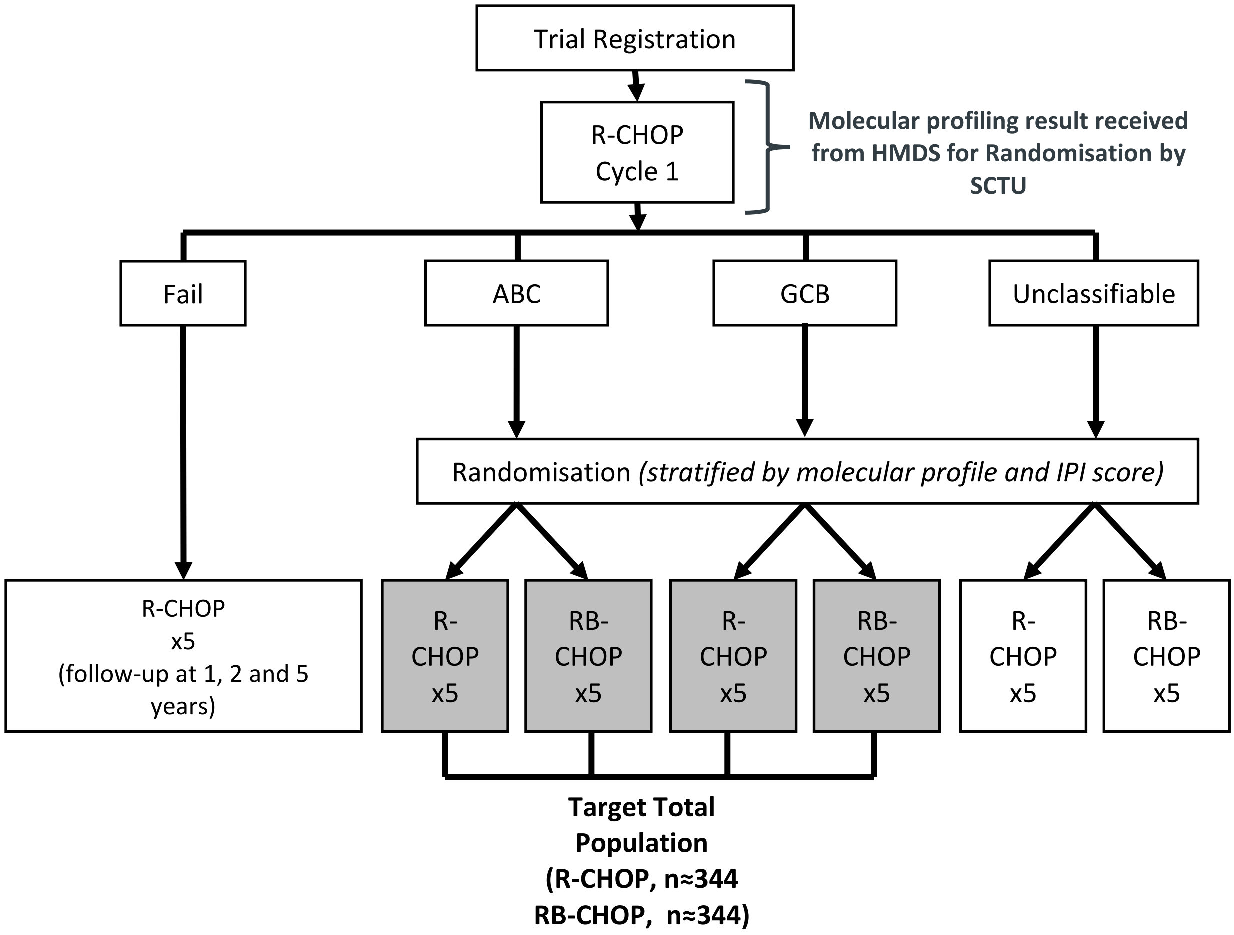

6.3 NUMBER OF PATIENTS/ASSIGNMENT TO TREATMENT ARMS

Initially, 940 patients were required to participate in this study after successfully completing the screening visit. It is expected that there will be a 15% RNA extraction failure rate from submitted FFPE tissue blocks. Following molecular profiling, it is anticipated, that 50% of patients will have GCB-DLBCL, 40% ABC-DLBCL and 10% will be unclassifiable. All GCB and ABC patients will be randomised on a 1:1 basis, with GCB or ABC stratification, between R-CHOP (Arm A) and RB- CHOP (Arm B) with 344 patients in each arm. Unclassified patients will be randomised along with ABC patients, but will not contribute to the total number required in this strata. Patients with RNA extraction failures will be treated with R-CHOP and followed according to arm A of the trial. Patient recruitment will continue until 344 patients have been randomised in each arm.

However, based on trial registrations up to 31-Dec-2013, 1132 patients will be required to be registered in this study. After allowing for a dropout rate of 4.5% from registration to the point of randomisation, it is expected that 1081 patients will reach the point of randomisation, of which 17.5% will be RNA extraction failures. Following molecular profiling, it is anticipated that 52% of patients will have GCB-DLBCL, 29.1% ABC-DLBCL and 18.8% will be unclassifiable i.e. of all the patients reaching the point of randomisation it is expected that 17.5% will be RNA extraction failures, 42.9% will have GCB-DLBCL, 24.1% will have ABC-DLBCL, and 15.5% will be unclassifiable (please see 15.3.1 for further details). All GCB and ABC patients will be randomised on a 1:1 basis, with GCB or ABC stratification, between R-CHOP (Arm A) and RB- CHOP (Arm B) with 344 patients in each arm. (please see 15.3.1 for further details).

In the event that either interim analysis (see section 15) results in closure of the GCB arm, recruitment will continue until 260 ABC patients have been randomised. In this instance, GCB patients will be treated and followed according to arm A of the trial. 'Unclassified' patients will continue to be randomised, but will not contribute to the total number of patients in the ABC arm.

Randomisation and treatment allocation will be performed, prior to administration of cycle 2, by the SCTU on receipt of molecular profiling information from HMDS. To avoid bias, molecular phenotypic data will not be disclosed to investigators.

6.4 Discontinuation of Trial Treatment

Patients will discontinue trial treatment for one of the following reasons:

1. Protocol violation.
2. Physician decision to withdraw patient.
3. Toxicity from study therapy.
4. Patient decision to withdraw from treatment.

Patients withdrawn from trial treatment will continue to be followed on the full pathway, according to the protocol, for months 3, 6, 9, 12, 18, 24, 30, 36, 42, 48, 54 and 60.

6.5 Disease Progression

If disease progression occurs the patient will discontinue trial treatment but will continue to be followed on the abbreviated pathway, according to the protocol, for months 12, 24 and 60

6.6 End of Study

The end of study will occur when **one** of the following events occurs:

1. The patient reaches the final follow up visit, which will be 5 years after receiving the last study treatment.
2. There is an eligibility screening failure, as the patient has been registered but subsequently discovered to be ineligible prior to randomisation.
3. Patient is lost to follow up.
4. Patient withdrew consent from follow up.

5. Death occurred.

The end of the study is defined as the date of the last follow-up visit of the last patient entered (to occur 5 years after receiving the last study treatment) or sooner, if all patients have progressed, died or withdrawn from the study.

Recruitment is expected over a 4 year period, with a planned study duration of 108 months.

6.7 CENTRE SELECTION

Centres will be selected on the basis of facilities, size and ability to report and assess safety data rapidly and effectively. It is expected that >80 will participate in the UK, and 15 in Switzerland.

Before the commencement of recruitment, each centre will be required to provide the following:

- Registered with the University of Southampton Clinical Trials Unit
- Completed Clinical Trial Site Agreement signed by the participating Trust
- Confirmation of participating Trust Research and Development approval

7 TRIAL OUTCOME MEASURES

7.1 PRIMARY OUTCOME MEASURE:

Progression-free survival

7.2 SECONDARY OUTCOME MEASURES:

Overall survival Event-
free survival Disease-
free survival Time to
progression Response
duration
Complete and overall response rates
Evaluation of toxicity (according to CTCAE version 4.0)
Quality of life and assessment of peripheral neuropathy (employing the EORTC QLQ- C30 tool with CIPN20 module for assessment of chemotherapy induced peripheral neuropathy)

8 SELECTION AND ENROLMENT OF PATIENTS

8.1 SCREENING AND PRE-REGISTRATION / RANDOMISATION EVALUATIONS

8.1.1 Molecular Profiling Consent

During the initial clinic visit where the main trial information sheet is offered and explained, patients will be given an information sheet and asked to sign a consent form for molecular profiling. This will allow their tumour block to be obtained and sent to HMDS for analysis (see Appendix 6) prior to the randomisation for which the result is required. Patients will be required to return at a later date to sign consent for the main trial if they wish to participate.

8.1.2 Consent to Main Trial

Patients who have signed an informed consent form will be evaluated for exclusion and inclusion criteria at the screening visit, which should take place within 14 days before the start of treatment.

The following assessments will take place within 14 days before the first dose of study medication. Investigation performed for the purposes of diagnosis and staging may be used as screening assessments provided they are performed within the appropriate trial window. The

CT scan, serology for Hepatitis B and C and echocardiogram/MUGA (if necessary) may be performed up to 42 days prior to planned therapy, and bone marrow up to 90 days prior to planned therapy.

- Medical history including demographics, previous and concurrent relevant diseases and medications.
- Physical examination
- Vital signs, including weight, height, blood pressure, pulse and temperature
- Recording of ECOG performance status
- 12 lead electrocardiogram is mandatory for all patients. In addition, an Echocardiogram or MUGA (as per local practice) should be performed for patients over the age of 70, and known diabetics over the age of 60. It should also be performed at baseline in all patients with a history of cardiac disease, treated hypertension or an abnormal resting ECG. These are not study specific tests, but represent normal clinical practice. Patients must have a left ventricular ejection fraction of $\geq$50% and should be considered fit enough to receive 300mg/m^2 doxorubicin
- Contrast enhanced CT of chest, abdomen and pelvis (neck if indicated). This should be carried out within 42 days of first study treatment. PET/CT hybrid scanners may be used to acquire the required CT images only if the CT produced by the scanner is of diagnostic quality and includes the use of intravenous (IV) contrast
- Bone marrow aspirate and trephine biopsy (single site with adequate trephine) within 90 days of first study treatment
- Serum chemistry to include sodium, potassium, urea, creatinine, bilirubin, alanine or aspartate transaminase, alkaline phosphatase, , albumin, total proteins, β2 microglobulin, LDH, calcium, phosphate and urate
- Full blood count to include haemoglobin, white cell count, absolute neutrophil count and platelets
- Serology for Hepatitis B and C. As per standard of care, prior to initiation of immunochemotherapy, the results of hepatitis serology should be known. In the acute situation, registration may occur without the results of the hepatitis serology, but must be available to the SCTU prior to randomization,
- Cerebrospinal fluid examination should be performed if clinically indicated or lymphomatous involvement of bone marrow, peripheral blood, nasal/paranasal sinuses or testis. CNS prophylaxis may be given according to local policy
- Diagnostic tumour block to be forwarded immediately upon obtaining informed consent to HMDS, Leeds, according to study procedure outlined in Investigator Site File (ISF).
- 2 x 4.5 ml EDTA samples required, to be forwarded to HMDS if UK centre, or IOSI Laboratory of experimental Oncology if Swiss centre. This to be done after registration so that patient ID number can be put on the samples, but prior to the patient receiving the first cycle of chemotherapy.

8.2 TARGET POPULATION

Previously untreated patients with CD20 positive diffuse large B-cell lymphoma (DLBCL) according to the WHO classification.

8.3 INCLUSION CRITERIA

- Histologically confirmed DLBCL, expressing CD20. Sufficient diagnostic material should be available to forward to HMDS for gene expression profiling and central pathology review. If there is doubt as to the quality of the tumour block, a second tumour block should be sent if available. Core biopsies are acceptable, however the molecular profiling success rate is inferior compared to larger surgically acquired tissue samples. Best diagnostic practice encourages investigators to seek the latter approach whenever clinically appropriate. If the only means to obtain histology is a core biopsy, ideally two cores should be taken and embedded separately, so that one can be sent to HMDS and one retained locally for diagnosis.
- Measurable disease of at least 15mm
- Not previously treated for lymphoma and fit enough to receive combination chemoimmunotherapy with curative intent
- Age $\geq$18 years

- Stage I_{AX} (bulk defined as lymph node diameter >10cm) to stage IV disease and deemed to require a full course of chemotherapy. Patients with non-bulky I_E disease are not eligible
- ECOG performance status 0-2
- Adequate bone marrow function with platelets $>100x10^9$/L; neutrophils $>1.0x10^9$/L at study entry, unless lower figures are attributable to lymphoma.
- Serum creatinine <150μmol/L, measured or calculated creatinine clearance>30mls/min, serum bilirubin <35μmol/L and transaminases <2.5x upper limit of normal at time of study entry, unless attributable to lymphoma.
- Cardiac function sufficient to tolerate 300mg/m^2 of doxorubicin. A pre-treatment echocardiogram is not mandated, but recommended in patients considered at higher risk of anthracycline cardiotoxicity
- No concurrent uncontrolled medical condition
- Life expectancy >3 months
- Adequate contraceptive precautions for all patients of child bearing potential
- A negative serum pregnancy test for females of child bearing potential or those <2 years after the onset of the menopause
- Patients will have provided written informed consent

8.4 EXCLUSION CRITERIA

Patients will be excluded from study entry if any of the following criteria are met

- Previous history of treated or untreated indolent lymphoma. However newly diagnosed patients with DLBCL who are found to also have small cell infiltration of the bone marrow or other diagnostic material (discordant lymphoma) will be eligible.
- Diagnosis of primary mediastinal lymphoma
- Uncontrolled systemic infection
- History of cardiac failure or uncontrolled angina
- Clinical CNS involvement
- Serological positivity for Hepatitis B, C, or known HIV infection. As per standard of care, prior to initiation of immunochemotherapy , the results of hepatitis serololgy should be known. In the acute situation, registration may occur without the results of the hepatitis serology, but must be available to the SCTU prior to randomisation,
    - Positive test results for chronic HBV infection (defined as positive HBsAg serology) will not be eligible.
    - Patients with occult or prior HBV infection (defined as negative HBsAg and positive total HBcAb) will not be eligible as one would normally monitor HBV DNA serially and add lamivudine if copy number became detectable. There is an interaction between lamivudine and bortezomib. Reactivation of latent infection has been reported with the use of bortezomib in this population (along obviously with the well recognised reactivation following R-CHOP). For these patient safety reasons, these patients should be excluded.
    - Patients who have protective titres of hepatitis B surface antibody (HBSAb) after vaccination are eligible.
    - Positive test results for hepatitis C (hepatitis C virus [HCV] antibody serology testing) will not be eligible.
- Serious medical or psychiatric illness likely to affect participation or that may compromise the ability to give informed consent.
- Active malignancy other than fully excised squamous or basal cell carcinoma of the skin or carcinoma in situ of the uterine cervix in the preceding 5 years.
- History of allergic reaction to substances containing boron or mannitol
- Patient unwilling to abstain from green tea and preparations made from green tea as bortezomib may interact with these.

8.5 PATIENT TRANSFERS

For patients moving from one area to another, every effort should be made for the patient to be followed up at another participating trial centre, and for this centre to take over responsibility for the patient. A copy of the patient's case report form (CRF) should be provided to the new site. The patient will be required

to sign a new consent form at the new site, and until this has happened, the patient remains the responsibility of the initial centre. The SCTU should be informed in writing of patient transfers.

9 REGISTRATION / RANDOMISATION PROCEDURES

Each patient must be given a patient information sheet and provide written informed consent, for both the Molecular Profiling and the main trial (as outlined in section 8.1.1 & 8.1.2) witnessed and signed by the researcher.

Once the patient has signed the Molecular Profiling Informed Consent Form, sites should notify the SCTU of the patient details (see Appendix 6). The SCTU will check the HMDS register for arrival of the patients tumour block. This should be sent immediately by site to allow sufficient time for work up of molecular phenotype.

9.1 REGISTRATION

The registration and baseline assessment form should be completed.

Only patients fulfilling all eligibility criteria should be registered and patients must be registered before cycle 1 of treatment commences. It is important to let the SCTU know a provisional planned date of administration of first cycle of R-CHOP chemotherapy. Any queries should be discussed with the SCTU before registration.

To register a patient:

- Fully complete and sign the REMoDL-B Patient Registration Form
- Fax form to 0844 XXXXXXX or scan and e-mail it to XXX@XXXX.ac.uk
- Alert SCTU that registration has been sent by phoning XXX XXXX XXXX
- Once the form is received the REMoDL-B trial coordinator will check eligibility of patient and delegation log, and then register the patient
- Confirmation of registration will be faxed, phoned or emailed to relevant site staff

Registrations can only be carried out during SCTU office hours:

Monday to Friday between 09.00 and 17.00

Any registration forms received out of hours will be processed on the next working day.

9.2 RANDOMISATION

The University of Southampton Clinical Trials Unit will be notified of the result of the molecular phenotype of DLBCL.
The patient will be randomised to one of two groups on a 1:1 ratio, with stratification for molecular phenotype and IPI.

Arm A	R-CHOP given every 21 days for 5 additional cycles
Arm B	RB-CHOP given every 21 days for 5 cycles.

Depending upon the results of the interim analysis, strata may close to randomisation.

The randomisation result will be faxed, phoned or emailed to the clinician entering the patient.

10 TREATMENTS

10.1 OVERVIEW

Patients will be registered and commence R-CHOP chemotherapy. Their diagnostic material should be forward to HMDS for molecular phenotyping. This result will be available prior to cycle 2 and the patient at this point will be randomised on a 1:1 basis (with stratification for IPI and molecular phenotype) between 5 further cycles of R-CHOP or 5 cycles of RB-CHOP.

Patients where molecular phenotyping fails will continue on the study and receive 5 further cycles of R-CHOP and be followed up for survival data at 1, 2 and 5 years. Those patients who are deemed molecularly unclassifiable will be randomised between R-CHOP and RB-CHOP.

On completion of trial allocated therapy, patients will undergo response evaluation and subsequently followed.

10.2 TREATMENT ARMS

10.2.1 All patients - first cycle

All patients will receive a single cycle of R-CHOP therapy (days 1-21).

A **pre-phase** will be permissible according to individual patient need and local policy. This approach may improve symptoms and performance status immediately prior to initiation of definitive therapy. This should not exceed 10 days of Prednisolone 100mg od (or equivalent) with or without vincristine 1mg.

The doses will be administered as shown below. For the purposes of this protocol, Day 1 is the day when the first cycle of R-CHOP is initiated. As rituximab and CHOP are to be administered on the same day, it is recommended that prednisolone be given prior to the rituximab infusion. The order and rate of administration will be determined by local practice. Dose banding will also be permitted for R-CHOP/RB-CHOP according to local policy.

For patients aged over 70, sites can reduce cycle 1 doxorubicin and cyclophosphamide by 25%.

No other first cycle dose modifications are permissible.

Premedication and administration schedule for rituximab will follow local procedures throughout both arms of the study.

10.2.1.1 R-CHOP: cycle1 (Days 1-21)

Drug	Dose	admin	Day				
			1	2	3	4	5
Rituximab	375 mg/m^2	IV infusion	√				
Cyclophosphamide	750 mg/m^2	IV bolus	√				
Vincristine	1.4 mg/m^2 (max 2mg)	IV infusion	√				
Doxorubicin	50mg/m^2	IV bolus	√				
Prednisolone	100mg	oral	√	√	√	√	√

10.3 TREATMENT ASSIGNMENT

Within 21 days (prior to planned commencement of second cycle) the Investigator will be notified by the SCTU of the results of the randomisation to either R-CHOP or RB-CHOP.

In order to ensure that Investigators are notified within the appropriate timelines, it is essential that the tissue block is forwarded to HMDS as soon as the patient has consented to trial entry.

10.4 ARM A: R-CHOP

A further 5 cycles (maximum) of R-CHOP will be given on a 21 day schedule as above.

10.5 ARM B: RB-CHOP

The following R-CHOP and bortezomib regimen will be given on a 21 day cycle to a maximum of 5 cycles. Bortezomib will be administered first on Days 1 and 8 subcutaneously

Drug	Dose	admin	Day					
			1	2	3	4	5	8
Bortezomib	1.6 mg/m^2	Sub-cut	√					√
Rituximab	375 mg/m^2	IV infusion	√					
Cyclophosphamide	750 mg/m^2	IV bolus	√					
Vincristine	1.4 mg/m^2 (max 2mg)	IV infusion	√					
Doxorubicin	50mg/m^2	IV bolus	√					
Prednisolone	100mg	oral	√	√	√	√	√	

10.6 CONTINUATION OF STUDY THERAPY

Patients will receive a maximum of 6 cycles of therapy (R-CHOP x1 followed by 5 further cycles of R-CHOP or 5 cycles of RB-CHOP) There will be no provision for treatment beyond 6 cycles. However, two additional doses of rituximab are permissible in line with NICE and local network policy guidelines.

If giving additional doses of rituximab, sites must:

- Pre-specify this to the SCTU in advance
- Administer to all patients regardless of treatment arm
- Document in the standard CRF pages

If the patient is felt to clinically require further therapy (excluding the two extra rituximab doses mentioned above, if applicable) upon completion of study treatment, this will be documented under 'second line induction therapy' and they will be followed up for survival only at years 1, 2 and 5 post treatment. Any additional therapy will be at the discretion of the local Investigator.

10.7 SUPPORTIVE CARE

Patients will receive standard anti-emetic as per local policy. Patients receiving bortezomib on day 8 may benefit from the administration of metoclopramide IV pretreatment, with oral medication as required post-therapy

Allopurinol 300mg od po should be given as per local practice.

Mouthcare and antacids are given according to local policy.

Diarrhoea and constipation are both recognised side effects of therapy in this protocol, diarrhoea is the most common. Treatment should be according to local policy, although prophylactic antidiarrhoeal agents are not recommended on the first cycle of RB-CHOP. Depending upon severity, loperamide should be prescribed for subsequent cycles for patients experiencing diarrhoea.

GCSF support may be given according to local policy for primary prophylaxis. Secondary prophylaxis may be given as per tables 11.1.1.1. and 11.2.1.1.

10.8 ADDITIONAL THERAPY FOR DLBCL

Additional therapy for DLBCL may be given according to local policy in the following manner:

10.8.1 Central nervous system prophylaxsis

Patients with lymphomatous involvement in bone marrow, peripheral blood, nasal/paranasal sinuses, orbit, paravertebral disease and testis are considered to have high risk for CNS disease as are those with a high LDH and multiple extranodal sites. They should receive prophylactic intrathecal methotrexate at 12.5mg per cycle for between three to six cycles, but this is at the Investigators discretion. The REMoDL-B protocol is permissive and CNS intrathecal prophylaxis should be according to their local policy. Intrathecal methotrexate must always be administered after all intravenous drugs are given. This should be administered according to local and national policies.

The use of prophylactic systemic administration of methotrexate is not permissible for randomized patients during R-CHOP or RB-CHOP therapy. This is because of a potential interaction with bortezomib. Its use is allowed after completion of protocol defined therapy and should be discussed with the clinical members of the Trial Management Group before initiation.

Patients who fail molecular profiling, and are therefore not randomised, may have systemic administration of methotrexate any time during therapy. This should be according to local hospital policy.

10.8.2 Radiotherapy

Following treatment with rituximab and CHOP +/- bortezomib, the use of radiotherapy to initial bulk disease, extra-nodal sites or residual disease is left to individual investigator’s discretion.

If patients receive radiotherapy, response assessment should be performed 1 month after completion.

10.9 PET IMAGING

Upon completion of protocol specified therapy, response assessment will be by CT. Sites may use PET/CT if the CT component can adequately assess response (see Section 14.2.6 of protocol), however if there is any doubt as to the quality of the PET/CT scan, a conventional CT scan should be performed.

In the event of a residual mass being identified a PET/CT may be performed according to local practice. This is not a study related procedure. Investigators are recommended to biopsy any areas suggestive of active disease before initiating additional anti-lymphoma therapy. PET abnormalities should be managed according to local practice. Data on additional therapy will be collected.

11 DOSE MODIFICATIONS FOR TOXICITY

11.1 R-CHOP

Further cycles of R-CHOP chemotherapy will be given when the neutrophils $\geq$1.0x109/l and the platelets are $\geq$100x109/l.

11.1.1 Haematological Toxicity

11.1.1.1 Neutropenia

Problem	Action
Neutrophils <1.0x10^9/l on proposed day 1 of cycle	Delay therapy until neutrophils ≥1.0x10^9/l. If not recovered after 14 days, withdraw patient from study. GCSF permissible as secondary prophylaxis according to local policy
Grade 4 neutropenia or any febrile neutropenia following any cycle of R-CHOP	All subsequent cycles of CHOP given with GCSF support according to local policy (an example: filgrastim 300μg once daily on days 5-12). The use of pegfilgrastim is permitted
Grade 4 neutropenia leading to infection despite GCSF support	Reduce dose of cyclophosphamide and doxorubicin by 50% for all subsequent cycles
Grade 4 neutropenia recurs despite 50% dose reduction in cyclophosphamide and doxorubicin	Withdraw patient from treatment

11.1.1.2 Thrombocytopenia

Problem	Action
Platelets <100x10^9/l on proposed day 1 of cycle	Delay therapy until platelets ≥100x10^9/l. If not recovered after 14 days, withdraw patient from study
Grade 4 thrombocytopenia following any cycle of R-CHOP	Reduce dose of cyclophosphamide and doxorubicin by 50% for all subsequent cycles
Grade 4 thrombocytopenia recurs despite 50% dose reduction in cyclophosphamide and doxorubicin	Withdraw patient from treatment

11.1.2 Non Haematological toxicity

11.1.2.1 Modification for neuropathic pain or peripheral neuropathy

If grade ≥2 motor or grade ≥3 sensory neurological toxicity to vincristine appears, the dose will be decreased to 1mg per cycle. If the neurological toxicity increases despite dose reduction, vincristine will be stopped.

11.1.2.2 Other non-Haematological toxicity

Dose reduction of individual medications can be considered if other toxicities such as severe mucositis occur, as per usual local practice. Grading of adverse event precipitating dose reduction should be recorded. Dose reductions for hepatic or renal impairment should be according to local policy.

11.2 RB-CHOP

Further cycles of RB-CHOP chemotherapy will be given when the neutrophils $\geq 1.0 \times 10^9$/l and the platelets are $\geq 100 \times 10^9$/l.

11.2.1 Haematological Toxicity

11.2.1.1 Neutropenia

Problem	Action
Neutrophils $<1.0 \times 10^9$/l on proposed day 1 of cycle	Delay therapy until neutrophils $\geq 1.0 \times 10^9$/l. If not recovered after 14 days, withdraw patient from study. GCSF permissible as secondary prophylaxis according to local policy
Grade 4 neutropenia or any febrile neutropenia following any cycle of RB-CHOP	All subsequent cycles of CHOP given with GCSF support according to local policy (an example: filgrastim 300µg once daily on days 5-12). The use of pegfilgrastim is permitted
Grade 4 neutropenia leading to infection despite GCSF support	Reduce dose of cyclophosphamide and doxorubicin by 50% for all subsequent cycles
Grade 4 neutropenia recurs despite 50% dose reduction in cyclophosphamide and doxorubicin	Withdraw patient from treatment

11.2.1.2 Thrombocytopenia

Problem	Action
Platelets $<100 \times 10^9$/l on proposed day 1 of cycle	Delay therapy until platelets $\geq 100 \times 10^9$/l. If not recovered after 14 days, withdraw patient from study
Platelets $<30 \times 10^9$/l on proposed day 8 of RB-CHOP cycle	Omit bortezomib on day 8. Restart next cycle with dose reduction of bortezomib by 1 level (see below) on days 1 and 8 Do not re-escalate dose on future cycles
Platelets $<30 \times 10^9$/l on proposed day 8 of RB-CHOP cycle despite dose reduction	Omit bortezomib on day 8. Restart next cycle with dose reduction of bortezomib by a further level (see below) on days 1 and 8 Do not re-escalate dose on future cycles
Platelets $<30 \times 10^9$/l on proposed day 8 of RB-CHOP cycle despite dose reduction recurs following any cycle of RB-CHOP despite second dose reduction in bortezomib	Omit bortezomib on all future cycles

Grade 3 or 4 thrombocytopenia recurs despite omission of bortezomib.	Reduce dose of cyclophosphamide and doxorubicin by 50% for all subsequent cycles
Grade 3 or 4 thrombocytopenia recurs Despite omission of bortezomib and 50% dose reduction in cyclophosphamide and doxorubicin	Withdraw patient from treatment

11.2.2 Non Haematological toxicity

11.2.2.1 Modification for neuropathic pain or peripheral neuropathy

There are two potentially neurotoxic agents in the RB-CHOP (Arm B) of REMoDL-B. Prompt recognition of neurological adverse events is critical.

In considering neuropathy its features should be considered separately and motor, sensory and neuropathic pain individually graded according to CTCAE 4.0.

If the degree of deficit reverses, it is possible to reintroduce agents, according to schedule.

The following process should be followed:

1 Assess and grade individual components of neuropathy according to CTCAE 4.0 on day 1 and 8 of cycle (Table below)

	Neuropathic pain (CTCAE 4.0 definition neuralgia/peripheral nerve)	Peripheral sensory neuropathy	Peripheral motor neuropathy	Autonomic neuropathy see below*
**0**	none	Normal	Normal	Normal
**1**	mild pain not interfering with function	Asymptomatic; loss of deep tendon reflexes or parasthesia	Asymptomatic; clinical or diagnostic observations only; intervention not indicated	Asymptomatic; clinical or diagnostic observations only; intervention not indicated
**2**	moderate pain; limiting instrumental ADL	Moderate symptoms; limiting instrumental ADL	Moderate symptoms; limiting instrumental ADL	Moderate symptoms; limiting instrumental ADL
**3**	severe pain; limiting self-care ADL	Severe symptoms; limiting self-care ADL	Severe symptoms; limiting self-care ADL; assistive device indicated	Severe symptoms; limiting self-care ADL; assistive device indicated
**4**		Life-threatening consequences; urgent intervention indicated	Life-threatening consequences; urgent intervention indicated	Life-threatening consequences; urgent intervention indicated

*autonomic neuropathy is not a CTCAE 4.0 stand-alone terminology. The above grading should be recorded, along with symptom specific grading

2 Use grading of neuropathy/neuropathic pain to determine required action.

These reductions should be applied unless causality of toxicity is DEFINITELY not related to one or more of these agents

**Day of Cycle**	**Severity of motor, sensory or autonomic neuropathy**	***and***	**Severity of Neuropathic Pain**	□	**Action/ Plan: Bortezomib and Vincristine**
Day 1	Grade 0 or 1	*and*	Grade 0 or 1 pain	□	No dose reduction
		and	Grade 2 pain	□	Withhold bortezomib; resume with 1 level dose reduction bortezomib when pain resolves to ≤ Grade 1
		and	Grade 3 pain	□	Withhold bortezomib; resume with 2 level dose reduction bortezomib when pain resolves to ≤ Grade 1
	Grade 2	*and*	Grade 0 or 1 pain	□	Drop 1 dose levels bortezomib<br>25% dose reduction of vincristine
		and	Grade 2 pain	□	Withhold both drugs; resume with reduction in bortezomib and 50% dose reduction in vincristine when pain resolves to ≤ Grade 1
		and	Grade 3 pain	□	Discontinue both drugs
	Grade 3	*and*	Grade 0, 1 or 2 pain	□	Withhold both drugs; resume with 2 level dose reduction and 50% vincristine dose reduction when neuropathy resolves to ≤ Grade 1 and/or pain resolves to ≤ Grade 1
		and	Grade 3 pain	□	Discontinue both drugs
Day 8	Grade 0 or 1	*and*	Grade 0 or 1 pain	□	No dose reduction
		and	Grade 2 or 3 pain	□	Withhold bortezomib; resume on day 1 according to guidelines
	Grade 2	*and*	Grade 0 or 1 pain	□	No further dose reduction if already reduced same cycle day 1, otherwise 1 level dose reduction bortezomib
		and	Grade 2 or 3 pain	□	Withhold bortezomib; resume on day 1 according to guidelines
	Grade 3	*and*	Grade 0, 1, 2 or 3 pain	□	Withhold bortezomib; resume on day 1 according to guidelines
	Grade 4	*and*	With or without pain	□	Discontinue bortezomib

3 Select current dosing level of bortezomib and reduce according to plan

Level	Dose
3	1.6 mg/m^2 per dose
2	1.2 mg/m^2 per dose
1	0.8 mg/m^2 per dose
0	discontinue

Patients may remain on study if the cycle is delayed for a maximum of 2 weeks. After 2 weeks patients may continue with other therapies but will continue on study on the abbreviated pathway.

11.2.2.2 Renal impairment

In the event of creatinine clearance (measure or calculated) being <20 mls/min at the beginning of the cycle, bortezomib should be withheld.

Dose reductions for other agents should be according to local policy.

11.2.2.3 Other non-Haematological toxicity

Dose reduction of individual medications can be considered if other toxicities such as severe mucositis occur, as per usual local practice. Grading of adverse event precipitating dose reduction should be recorded. Dose reductions for hepatic or renal impairment should be according to local policy.

In cases where the patient is unable to tolerate the s/c bortezomib the patient may switch back to the IV administration. In this situation the reason for the patient's inability to tolerate s/c bortezomib must be reported as an Adverse Event and clinician must provide an e-mail to the SCTU detailing the reason for the need to revert back to the IV route of administration. This shall be passed on to Janssen who shall review each case. Once approved, Janssen shall provide patient specific IV bortezomib vials for the remaining cycles.

11.3 INTERACTION WITH OTHER DRUGS

11.3.1 Patients receiving bortezomib

In vitro studies indicate that bortezomib is a weak inhibitor of the cytochrome P450 (CYP) isozymes 1A2, 2C9, 2C19, 2D6 and 3A4. There is minimal data on drug interactions with bortezomib. Patients should be closely monitored when given bortezomib in combination with potent CYP3A4 inhibitors and CYP3A4 inducers. A list of many of the drugs with the above properties is listed in Appendix 8.

During clinical trials, hypoglycemia and hyperglycemia were uncommonly and commonly reported in diabetic patients receiving oral hypoglycemics. Patients on oral antidiabetic agents receiving bortezomib therapy treatment will require close monitoring of their blood glucose levels and adjustment of the dose of their oral hypoglycaemic accordingly.

11.4 STUDY THERAPIES

Bortezomib will be centrally supplied free of charge for this clinical trial by Janssen Cilag. Bortezomib will be ordered via the University of Southampton Clinical Trials Unit using the fax provided and delivered by Fisher Clinical Supplies. Delivery will take 8 working days from ordering to arrival at site. Each carton

will contain 10 vials of bortezomib (for 5 cycles of treatment). The bortezomib supplied is trial specific not patient specific and as such any unused vials should be used for the next patient allocated to arm B.

Other study treatments are standard of care and will be from local supplies.

11.5 ACCOUNTABILITY

The Principal Investigator (PI) is fully responsible for the IMPs at the site. Dispensing of medication may be delegated to a hospital pharmacy as locally applicable.

The person responsible for dispensing the medication will be responsible for maintaining adequate control of the IMPs and for documenting all transactions relating to them (as a minimum batch number, expiry date and dispense date must be documented on the Drug Accountability Logs). IMPs must be stored in a safe and secure place (only accessible to authorized personnel), and proper dispensing arrangements must be made.

11.6 CO-ENROLLMENT GUIDELINES

Patients in REMoDL-B should not be recruited into other studies of anti-lymphoma treatments prior to disease progression, nor should they be already participating in other trials of anti- lymphoma therapy prior to study entry

Individuals participating in a trial testing a medicinal product unrelated to lymphoma will not be excluded and patients will be allowed to enter other trials that do not involve anti- lymphoma therapy.

Any queries should be addressed to the REMoDL-B Clinical Trials Coordinator

12 ASSESSMENT AND FOLLOW-UP OF PATIENTS

12.1 ONE MONTH AFTER THE FINAL DOSE OF THERAPY

Response to therapy will be made 1 month after the administration of the final dose of chemotherapy. If radiotherapy is given, this assessment will follow the radiation. If patients are given two additional doses of rituximab, follow-up will occur 1 month after day one of cycle 8 of the rituximab dose.

If patients receive radiotherapy. Response assessment should be performed one month after completion of radiotherapy.

<u>The following will be performed:</u>

A medical history, physical examination and recording of performance status will be performed along with routine laboratory tests: full blood count and biochemistry screen, including Lactate Dehydrogenase level.

A CT scan of the chest, abdomen and pelvis (with neck if indicated).PET/CT hybrid scanners may be used to acquire the required CT images only if the CT produced by the scanner is of diagnostic quality and includes the use of intravenous (IV) contrast

NOTE: *Upon completion of protocol specified therapy, response assessment will be by CT. In the event of a residual mass being identified a PET/CT may be performed according to local practice. This is not a study related procedure. Investigators are recommended to biopsy any areas suggestive of active disease before initiating additional anti-lymphoma therapy. PET abnormalities should be managed according to local practice. Data on additional therapy will be collected.*

A bone marrow aspirate and trephine will be performed, if previously involved

EORTC QLQ-C30 + CIPN20

Assessment of toxicity and adverse events

Response will be Investigator assessed by the criteria of the International Workshop Standardised Response Criteria for non-Hodgkin Lymphoma (Cheson, et al 1999). See section 14.2.6 for definitions.

Patients who fail molecular profiling will only be followed-up at year 1, 2 and 5 for survival data.

12.2 SUBSEQUENT FOLLOW- UP VISITS

Subsequent study visits will be performed at months 3, 6, 9, 12, 18, 24, 30, 36, 42, 48, 54 and 60 following completion of all therapy.

At each follow-up visit a medical history will be taken, a physical examination performed and the performance status recorded. Full blood count and Lactate Dehydrogenase levels are required as part of the routine follow-up.

The EORTC QLQ-C30 + CIPN20 questionnaires will be administered at months 3, 6, 9 12, 18, 24, 36, 48 and 60.

A single CT scan of the chest, abdomen and pelvis (with neck if indicated) will be performed at **12 months** following completion of protocol specified therapy, or on clinical suspicion of disease progression.

12.3 ASSESSMENTS FOR PATIENTS WITHDRAWN FROM TRIAL THERAPY

Patients who have been withdrawn from study therapy will be followed up according to the above schedule until disease progression is documented or second-line induction therapy is initiated. In these instances, patients will be followed for survival.

12.4 SAFETY ASSESSMENT

Safety will be assessed through the reporting of adverse events as described in section 13. Adverse events should be reported according to the National Cancer Institute Common Terminology Criteria for Adverse Events (CTCAE) version 4.0 (June 2010) (see Appendix 4)

12.5 QUALITY OF LIFE ASSESSMENT

The EORTC QLQ-c30 and chemotherapy induced peripheral neuropathy module (CIPN20) will be used to assess quality of life (Appendix 5). This will be administered prior to the beginning of each chemotherapy, at month 1 response assessment visit then at months 3, 6, 9, 12, 18, 24, 36, 48 and 60.

12.6 TRANSLATIONAL RESEARCH

This study aims to prospectively validate the cell of origin (ABC/GCB) model of DLBCL and its clinical practicality and utility, as well as assessing the benefit/toxicity of the addition of bortezomib to R-CHOP chemotherapy. Molecular profiling of tumour material will generate considerable amount of data for use in translational research to assess candidate and novel biomarkers.

The forwarding of formalin-fixed paraffin embedded (FFPE) lymph node biopsies to HMDS is of course critical to the success of REMoDL-B. Molecular phenotyping will be performed using the DASL array platform requiring the extraction of RNA. Central pathological review will be performed as a quality check but not fed back to sites unless findings untoward.. Additional material will be used for immunohistochemical, cytogenetic and molecular genetic studies. Material will only be retained if the sample size is sufficient for removal of material without compromising its value as archived diagnostic material. This may include the generation of extended full section immunohistochemical studies, tissue micorarrays and DNA extraction. The remaining FFPE block will be returned to the local pathologist.

DNA extracted from tumour material will be used to perform mutation detection on NF-κB pathway genes (eg *A20, ABIN-1, ABIN-2, CARD11*) Genetic abnormalities of NF-κB regulators will be correlated with clinical outcomes and with the expression of NF-κB target genes (such as CCND2, IRF4, cFLIP, BCL2, CCR7, IκBα). The latter will be explored as a surrogate marker for prognosis and prediction of treatment response.

In addition two 4.5 ml samples are required

i) For isolation of lymphocytes and FACS analysis at HMDS
ii) For storage and future extraction of germline DNA

Researchers wishing to access stored REMoDL-B samples will be required to submit a one project proposal form (available from the REMoDL-B Clinical Trials Coordinator) documenting a study outline, type of material required and sample quantities. Requests will be forward to the NCRI Lymphoma Clinical Studies Group who will seek peer review. They will decide, with appropriate support, whether to grant permission for the work. Evidence of ethical approval for the project will be required, if outside that granted in the parent trial, before any samples are released. A Material Transfer Agreement will also be signed before sample release.

12.7 PATIENTS LOST TO FOLLOW-UP

If a patient is lost to follow-up the local Principal Investigator along with the patient's usual clinician should attempt contact. Failing this, the patient's GP will be contacted and requested to provide follow-up information where possible.

13 PHARMACOVIGILANCE

13.1 DEFINITIONS

The Medicines for Human Use (Clinical Trials) Regulations 2004, as amended, provides the following definitions relating to adverse events in trials with an investigational medicinal product:

Adverse Event (AE): any untoward medical occurrence in a patient or clinical trial patient administered a medicinal product and which does not necessarily have a causal relationship with this treatment.
An AE can therefore be any unfavourable and unintended sign (including an abnormal laboratory finding), symptom, or disease temporally associated with the use of an investigational medicinal product (IMP), whether or not considered related to the IMP.

Adverse Reaction (AR): all untoward and unintended responses to an IMP related to any dose administered.
All AEs judged by either the reporting investigator or the sponsor as having reasonable causal relationship to a medicinal product qualify as adverse reactions. The expression reasonable causal relationship means to convey in general that there is evidence or argument to suggest a causal relationship.

Unexpected Adverse Reaction: an AR, the nature or severity of which is not consistent with the applicable product information (e.g. investigator's brochure (IB) for an unapproved investigational product or summary of product characteristics (SmPC) for an authorised product*).*
When the outcome of the adverse reaction is not consistent with the applicable product information this adverse reaction should be considered as unexpected. Side effects documented in the IB/SmPC which occur in a more severe form than anticipated are also considered to be unexpected.

Serious Adverse Event (SAE) or **Serious Adverse Reaction:** any untoward medical occurrence or effect that at any dose:

- **Results in death**

- **Is life-threatening** – *refers to an event in which the patient was at risk of death at the time of the event; it does not refer to an event which hypothetically might have caused death if it were more severe*
- **Requires hospitalisation, or prolongation of existing inpatients' hospitalisation**
- **Results in persistent or significant disability or incapacity**
- **Is a congenital anomaly or birth defect**

Medical judgement should be exercised in deciding whether an AE/AR is serious in other situations. Important AE/ARs that are not immediately life-threatening or do not result in death or hospitalisation but may jeopardise the patient or may require intervention to prevent one of the other outcomes listed in the definition above, should also be considered serious.

Suspected Unexpected Serious Adverse Reaction (SUSAR): any suspected adverse reaction related to an IMP that is both unexpected and serious.

13.2 CAUSALITY

Most adverse events and adverse drug reactions that occur in this trial, whether they are serious or not, will be expected treatment-related toxicities due to the drugs used in this trial. The assignment of the causality should be made by the investigator responsible for the care of the patient using the definitions in the table below.

If any doubt about the causality exists the local investigator should inform the Southampton Clinical Trials Unit (SCTU) who will notify the Chief Investigator. Pharmaceutical companies and/or other clinicians may be asked for advice in these cases.

In the case of discrepant views on causality between the investigator and others, all parties will discuss the case. In the event that no agreement is made, the MHRA will be informed of both points of view.

Relationship	Description
**Unrelated**	There is no evidence of any causal relationship.
**Unlikely**	There is little evidence to suggest there is a causal relationship (e.g. the event did not occur within a reasonable time after administration of the trial medication). There is another reasonable explanation for the event (e.g. the patient's clinical condition, other concomitant treatment).
**Possible**	There is some evidence to suggest a causal relationship (e.g. because the event occurs within a reasonable time after administration of the trial medication). However, the influence of other factors may have contributed to the event (e.g. the patient's clinical condition, other concomitant treatments).
**Probable**	There is evidence to suggest a causal relationship and the influence of other factors is unlikely.
**Definitely**	There is clear evidence to suggest a causal relationship and other possible contributing factors can be ruled out.

13.3 REPORTING PROCEDURES

All adverse events following patient's registration in the study will be reported. Depending on the nature of the event the reporting procedures below should be followed. Any questions concerning adverse event reporting should be directed to the SCTU in the first instance. A flowchart will be provided to aid in the reporting procedures.

13.3.1 Pre-existing Conditions

A pre-existing condition should not be reported as an AE unless the condition worsens by at least one CTCAE grade during the trial. The condition, however, must be reported in the pre- treatment section of the CRF, if symptomatic at the time of entry, or under concurrent medical conditions if asymptomatic.

13.3.2 Non serious AR/AEs

All such toxicities, whether expected or not, should be recorded on the Adverse Events Form. This should be completed continuously throughout the patient's treatment cycles. Once treatment is completed the AE form should be returned to the SCTU with the End of Treatment CRF. For patients who fail molecular profiling, non-serious AR/AEs will not be recorded.

13.3.3 Serious Adverse Events and Reactions

Fatal or life threatening SAEs and SUSARs should be reported within 24 hours of the local site becoming aware of the event. The SAE/SUSAR form asks for nature of event, date of onset, severity, corrective therapies given, outcome and causality (i.e. unrelated, unlikely, possible, probably, definitely). The responsible investigator should assign the causality and expectedness of the event with reference to the current IMP IB/SmPC (please see Appendix 7)and use the event terms and grades given in the NCI CTCAE v4.0. Additional information should be provided as soon as possible if the event/reaction has not resolved at the time of reporting.

All patients hospitalised with grade 4 neutropenia or grade 4 thrombocytopenia should be reported as an SAE/SAR.

Patients experiencing grade 4 neutropenia or grade 4 thrombocytopenia without hospitalisation will not need to be reported as a SAE per Chief Investigator decision that neutropenia and thrombocytopenia in this study is not usually life-threatening. If local investigator believes neutropenia is life-threatening for any patient, a SAE should still be submitted.

Relapse and death due to lymphoma, and hospitalisations for elective treatment of a pre- existing condition or routine blood transfusion do not need reporting as an SAE.

13.3.4 Reporting Details

An SAE/SUSAR form should be completed for all SAEs and SUSARS and faxed to the SCTU within 24 hours.

Complete the SAE/SUSAR form & fax or email a scanned copy of the form with as many details as possible to the SCTU together with <u>anonymised</u> relevant treatment forms and investigation reports.

Or

Contact the SCTU by phone for advice and then fax or email a scanned copy of the completed SAE/SUSAR form.

SAE/SUSAR REPORTING CONTACT DETAILS

Fax: XXXX XXX XXXX or Email: XXX@XXXX.ac.uk

FAO: Quality and Regulatory Team

Please <u>send</u> original SAE/SUSAR forms to:

SCTU

Tel: XXX XXXX XXXX (Mon to Fri 09.00 – 17.00)

The SCTU and SAKK (Swiss Group for Clinical Cancer Research) will notify the relevant competent authorities and main ethics committees of all SUSARs occurring during the trial according to the following timelines in accordance with the SAE reporting procedure: fatal and life-threatening within 7 days of notification and non-life threatening within 15 days. All investigators will be informed of all SUSARs occurring throughout the trial.

Local investigators should report any SUSARs and /or SAEs as required by their Local Research Ethics Committee and/or Research & Development Office.

13.3.5 Follow Up and Post-study SAEs and AEs

The reporting requirement for SAEs and AEs affecting patients applies for all events occurring up to 30 days after the last administration of study drugs. All unresolved adverse events should be followed by the investigator until resolved, the patient is lost to follow-up, or the adverse event is otherwise explained. At the last scheduled visit, the investigator should instruct each patient to report any subsequent event(s) that the patient, or the patient's general practitioner, believes might reasonably be related to participation in this study. The investigator should notify the study sponsor of any death or adverse event occurring at any time after a patient has discontinued or terminated study participation that may reasonably be related to this study.

13.3.6 SARs not requiring Immediate Reporting

The summary of product characteristics (SmPC) for each of the IMPs being used in this trial will be circulated to participating centres by the SCTU at initiation and is to be stored in the local site file. It is the responsibility of the PI at each site to check for the most recent version of the SmPC (see Appendix 7) produced by the correct manufacturer (where there are multiple) as sourced by their Trust pharmacy department.

Side effects and toxicities that are expected are listed in the SmPC(s) and do not require immediate reporting in this trial, unless they are of an unexpected severity. Death as a result of disease progression and other events that are primary or secondary outcome measures are also not considered to be SAEs.

SAEs of the above types should be recorded on the SAE/SUSAR form provided and this should be forwarded to the SCTU in a timely manner.

13.3.7 Pregnancy

If a patient or his/her partner becomes pregnant whilst taking part in the trial or during a stage where the foetus could have been exposed to an IMP, the investigator must inform the SCTU, complete the pregnancy form and fax to the SCTU within 24 hours of being made aware, and ensure that the patient and the patient's healthcare professional are aware that follow up information is required on the outcome of the pregnancy. If the patient leaves the area, their new healthcare professional should also be informed.

14 DEFINITION OF ENDPOINTS

14.1 PRIMARY ENDPOINT

The primary endpoint is progression-free survival (PFS). This is defined as the time from the day of registration to the date of progression or death from any cause.

14.2 SECONDARY ENDPOINTS

14.2.1 Overall survival

Overall survival will be measured from the day of registration to the date of death from any cause

14.2.2 Event-free survival

Event-free survival (time to treatment failure) is measured from the day of registration to any treatment failure including disease progression, or discontinuation of treatment for any reason (eg, disease progression, toxicity, patient preference, initiation of new treatment without documented progression, or death).

14.2.3 Disease-free survival

Disease-free survival will be measured from the time of documentation of disease-free state (CR or CRu) to disease recurrence or death as a result of lymphoma or acute toxicity of treatment.

14.2.4 Time to Progression

Time to progression (TTP) is defined as the time from registration until documented lymphoma progression or death as a result of lymphoma. Deaths from other causes are censored at the time of death.

14.2.5 Response Duration

Response duration is defined as the time from documentation of response (ie,CR, CRu or PR) until the documentation of relapse or progression.

14.2.6 Response Evaluation

Response will be assessed in accordance with the International Workshop Standardized Response Criteria for Non-Hodgkin's Lymphoma (Cheson, *et al* 1999).

The proportions of patients that experience CR, CRu and PR will be presented separately for each treatment and molecular group.

Response criteria will be determined as follows:

14.2.6.1 Complete response (CR)

Complete response (CR) requires **all** of the following criteria are met:

a) Complete disappearance of all detectable clinical and radiographic evidence of disease and disappearance of all disease-related symptoms if present before therapy and normalisation of those biochemical abnormalities (e.g. LDH definitely contributable to NHL)

b) All lymph nodes and nodal masses must have regressed to normal size (≤1.5cm in their greatest transverse diameter for nodes >1.5cm before therapy). Previously involved nodes that were 1.1 to 1.5 cm in their greatest transverse diameter before treatment must have decreased to ≤1.0 cm in their greatest transverse diameter after treatment, or by more than
75% in the sum of the products of the greatest diameters (SPD).

c) The spleen, if considered to be enlarged before therapy on the basis of a CT scan, must have regressed in size and must not be palpable on physical examination. However, no normal size can be specified because of the difficulties in accurately evaluating splenic and hepatic size. Any macroscopic nodules in any organs detectable on imaging techniques should no longer be present.

Similarly, other organs considered enlarged before therapy due to involvement by lymphoma, such as liver and kidneys, must have decreased in size.

d) If the bone marrow was involved by lymphoma before treatment, the infiltrate must be cleared on repeat bone marrow aspirate and biopsy of the same site. Flow cytometric, molecular or cytogenetic studies are not considered part of routine assessment to document persistent disease at the present time.

14.2.6.2 Complete response, undocumented/unconfirmed (CRu)

Complete response, undocumented/unconfirmed (CRu) includes those patients who fulfil criteria a) and c) above, but with one or more of the following features:

a) A residual lymph node mass greater than 1.5cm in greatest transverse diameter that has regressed by more than 75% in the SPD. Individual nodes that were previously confluent must have regressed by more than 75% in their SPD compared with the size of the original mass.

b) Indeterminate bone marrow (increased number or size of aggregates without cytological or architectural atypia).

14.2.6.3 Partial response (PR)

Partial response (PR) requires all of the following:

a) ≥50% decrease in SPD of the six largest dominant nodes or nodal masses. These nodes or masses should be selected according to the following features: (i) they should be clearly measurable in at least two perpendicular dimensions, (ii) they should be from as disparate regions of the body as possible, and (iii) they should include mediastinal and retroperitoneal areas of disease whenever these sites are involved.

b) No increase in the size of the other nodes, liver or spleen.

c) Splenic and hepatic nodules must regress by at least 50% in the SPD.

d) With the exception of splenic and hepatic nodules, involvement of other organs is considered assessable and not measurable disease

e) Bone marrow assessment is irrelevant for determination of a PR because it is assessable and not measurable disease

f) No new sites of disease

14.2.6.4 Stable disease (SD):

Neither sufficient shrinkage to qualify for partial response nor sufficient increase to qualify for progressive disease.

14.2.6.5 Relapsed and Progressive disease (PD) Relapsed

disease (from CR, CRu) requires the following:

a) Appearance of any new lesion or increase by ≥ 50% in the size of previously involved sites.

b) ≥50% increase in greatest diameter of any previously identified node greater than 1 cm in its short axis or in the SPD of more than one node.

Progressive disease (PD) following PR or SD requires one of the following:

a) ≥50% increase from nadir in the SPD of any previously identified abnormal node for PRs or non responders.

b) Appearance of any new lesion during or at the end of therapy

Relapsed and Progressive disease will be considered together

14.2.6.6 Summary of response criteria

Response	Nodal Mass	Liver/spleen	Bone marrow	Lymphoma related symptoms and physical examination
**CR**	mass >1.5cm in GTD should have regressed diameter to <1.5cm<br><br>Nodes 1.1-1.5cm in GTD diameter need to have regressed to <1.0cm or >75% SPD	Normal size, not palpable	No infiltration	Normal
**CR(u)**	As for CR	Normal size, not palpable	Indeterminate	Normal
	Mass >1.5cm in GTD regressed by >75% in the SPD<br><br>Individual masses that were previously confluent must have regressed by >75% in their SPD compared to size of the original mass	Normal size, not palpable	Normal or indeterminate	Normal
**PR**	As for CR	Normal size, not palpable	Positive	
	>50% decrease in SPD of 6 largest nodes<br>No increase in size of other nodes/new sites	Nodules reduced by >50%	Irrelevant	Decrease in size of liver/spleen
**SD**	Does not fulfil criteria of CR, CR(U), PR or PD			
**PD**	>50% increase in SPD from nadir (if PR or SD)<br>New lesions (>1.5 cm)		New or recurrent involvement	Enlarging liver/spleen<br><br>New disease sites

GTD; Greatest transverse diameter. SPD; sum of products greatest diameter

14.2.6.7 PET/CT Response Assessment

PET scanning is not used for response assessment in REMoDL-B. In the event of a residual mass being identified a PET/CT may be performed according to local practice. This is not a study related procedure.

Should a PET/CT be performed the TMG request that this information is not used in the determination of response. However, Investigators will be invited to supply this information on the CRF according to the International Workshop Revised Response Criteria for Malignant Lymphoma (Cheson, *et al* 2007). This will provide valuable data in order to validate the revised criteria.

14.2.7 Toxicity

Toxicity will be assessed through the reporting of adverse events as described in section 13. Adverse events should be reported according to the National Cancer Institute Common Terminology Criteria for Adverse Events (CTCAE) version 4.0 (June 2010) (see Appendix 4).

14.2.8 Quality of Life

The EORTC QLQ-C30 and chemotherapy induced peripheral neuropathy module (CIPN20) will be used to assess quality of life (Appendix 5).

14.3 DEFINITION OF END OF TRIAL

The trial will be considered closed 5 years after the last patient has completed their treatment. However further observational follow-up of all patients enrolled in the trial will continue until all randomised patients have died. This will initially be via the hospital but in the long term may employ national registers.

Investigators will be informed when patient recruitment ceases.

The TSC may prematurely discontinue the trial. Any such decision will be notified to the MHRA and REC.

15 STATISTICS AND DATA ANALYSIS

The trial uses a novel adaptive design (Simon 2008) with the initial hypothesis that the addition of bortezomib to R-CHOP is equally effective in both GCB and ABC type DLBCL. The stratification methodology restricts a priori assumptions, but uses a prospective analysis plan to evaluate the efficacy of the bortezomib overall and in the predefined subsets of ABC and GCB. An interim safety analysis is included in the GCB where the biology does not predict that the addition of bortezomib will be of value, and allows for closure in the event of a low early PFS. A second interim analysis is designed to seek efficacy in the GCB arm. In the event that there is no evidence that the addition of bortezomib to R-CHOP may be improving efficacy, the GCB strata will be closed. In this event the final analysis will be restricted to the ABC group in which the biological rationale is strongest.

15.1 EARLY STOPPING RULES

Early stopping rules are integral to the iterative design of this study:

1. A safety analysis will be performed after the first 55 GCB R-CHOP+B patients have been recruited. The analysis will be restricted to this group of patients only. This is based upon a Case Morgan design (Case and Morgan 2003) with the *a prior* knowledge (from the UK NCRI R-CHOP 14 vs. 21 study) that PFS at this time across all R-CHOP treated patients is 90%, and will mandate early stopping of the GCB group if the 1 year progression free survival is <70% (two sided significance level 5%, power 90%). This will protect against bortezomib actually being detrimental in the good prognosis GCB group. In the event of termination of the GCB strata, all GCB patients will receive R-CHOP and will be evaluated and followed as per protocol.

2. A second analysis will be performed for futility in the GCB group. This analysis will be performed after 73 patients have been randomised to receive R-CHOP+bortezomib and followed for one year (Case Morgan design with two sided significance level 5%, power 90%). If at one year the estimate of PFS is <85%, further exploration of bortezomib in this group of patients is not warranted, based upon the data from the previous study using molecular profiling, in which the figure for the GCB cases was 85% (Lenz, *et al* 2008b). In this case randomisation in the GCB group will be terminated on the grounds that the addition of bortezomib is highly unlikely to show an advantage. In the event of termination of the GCB strata, all GCB patients will receive R-CHOP and will be evaluated and followed as per protocol. However, if the estimate of PFS in the GCB R-CHOP+B group is ≥85%, continued exploration of this group is of interest as it suggests the possibility of benefit from bortezomib in GCB patients and randomisation in this group will continue.

In the event of closure of the GCB strata, ABC and unclassified patients will continue to be randomised between R-CHOP and R-CHOP+B, however sample size calculations will be revised and will be based upon the number of confirmed ABC patients (See statistical methodology). In this event, Investigators will be informed of the results of patients who are of GCB phenotype. There will be no disclosure of phenotype for those with failed molecular phenotype, ABC or unclassified to the local Investigator. These patients will continue to be treated according to study protocol.

15.2 STUDY POPULATIONS

The All Patients Registered population all the patients that have been registered for REMoDL-B.

The Intention-To-Treat population comprises all patients that are registered for REMoDL-B and reach the point of randomisation i.e. are either successfully randomised or have a confirmed failed RNA extraction.

The Safety population will comprise all patients who receive at least 1 dose of any study drug. The Response

Evaluable population is defined as all patients who have measurable disease at baseline, receive at least 1 dose of any study drug, and have at least 1 post-baseline response assessment.

15.3 SAMPLE SIZE AND POWER CALCULATIONS

Based upon pilot experience with the extraction of suitable RNA from FFPE tumour blocks and quality control of the DASL arrays, technical failure is expected in 15% of patients. These patients will not proceed to the treatment phase, and will receive conventional R-CHOP.

In the R-CHOP 14 vs 21 study, the progression free survival (PFS) rate of all R-CHOP treated patients was 75% at 30 months. This trial aims to detect an improvement in 30 month progression free survival of 10%, that is to 85%, with a significance level of 5% (two-sided) and 90% power. Patients will be randomised 1:1 between R-CHOP and R-CHOP+B with stratification by GCB or ABC subset and International Prognostic Index. The International Prognostic Index will be grouped as follows: low, low and high intermediate grouped together and high for the purpose of stratification. A total of 129 events are required to observe this difference and will require 344 patients to be randomised in each arm. Thus 688 patients defined as either GCB or ABC will be randomised. Patients deemed 'unclassified' by gene expression profiling, estimated to be 14% of the total, will be stratified in the ABC arm according to their predicted biological behaviour (Lenz, et al 2008b), but a total of 800 patients will be randomised in order to have a sufficient number of successfully typed cases. Randomisation will be performed over a 3.75 year period, with a further 5 years of follow-up. Based upon pilot data, we will allow for a 15% RNA extraction failure rate from tissue blocks, leading to the need to register a total of 940 patients at the maximum (however, please see revision of sample size below).

15.3.1 Revised Sample Size

Based on trial registrations up to 31-Dec-2013, 29 out of 640 (4.5%) patients registered withdrew from the study before the point of randomisation. This resulted in 611 patients reaching the point of randomization, of which:

- 107 (17.5%) were RNA extraction failures;
- 147 (24.1%) were of ABC molecular phenotype;
- 262 (42.9%) were of GCB molecular phenotype;
- 95 (15.5%) were of Unclassifiable molecular phenotype.

This leads to the need to register a total of 1132 patients. Allowing for a dropout of 51 patients (4.5%) should ensure that 1081 patients reach the point of randomisation, of which:

- 189 (17.5%) should be RNA extraction failures;
- 260 (24.1%) should be of ABC molecular phenotype;
- 464 (42.9%) should be of GCB molecular phenotype;
- 168 (15.5%) should be of Unclassifiable molecular phenotype.

The reported 30 month PFS in ABC patients treated with R-CHOP is 40% (Lenz, et al 2008b). An improvement in 30 month PFS to >60% with the addition of bortezomib would be considered a practice changing intervention. With 5% two sided significance level and a power of 90%, a total of 260 confirmed ABC patients would need to be randomised, with full analysis after 123 events.

If the GCB strata are closed after the second interim analysis (i.e. after 73 GCB R-CHOP+B patients) the total number of patients randomised to that point would be 530. Given that ABC patients are anticipated to represent 40% of the patient population, to gain 260 ABC randomisations a total of 650 successful array results would be required.

Therefore given the different possible scenarios, between 560 and 892 patients will be randomised depending upon the results of the interim analyses. Taking into account the expected RNA extraction failure rate and the likely number of unclassifiable cases, a total of between 765 and 1132 patients will need to be registered. These numbers may change depending on the ratio of ABC patients to GBC patients recruited to the study. If the RNA failure rate is higher than expected and/or the ABC or GCB rates are lower than expected, for example, the sample size will be adjusted accordingly to recruit and randomise 688 ABC+GCB patients. (Please see table on page 55)

		Total No. of patients	Total with successful molecular profile	Randomised		Not classified
				ABC	GCB	
1 GCB and ABC strata remain open						
	1 year PFS in GCB R-CHOP+B ≥ 85% patients	1132	891	260	463	168
	446 patients will be randomised to receive R-CHOP+bortezomib					
2 GCB strata close after first interim analysis: 1 year PFS <70% in 55 GCB R-CHOP+B patients						
	Patients before GCB strata closed	567	452	181	208	63
	Total to randomise 260 ABC	765	650	260	208	91
	280 patients will be randomised to receive R-CHOP+bortezomib					
3 GCB strata close after second interim analysis: 1 year PFS <85% in 73 GCB R-CHOP+ B patients						
	Patients before GCB strata closed	623	530	212	244	74
	Total to randomise 260 ABC	765	650	260	244	91
	298 patients will be randomised to receive R-CHOP+bortezomib					

15.3.2 Timing of primary analyses

The primary analysis is planned to take place when all registered patients have been followed up for a median of 30 months (except those who progress, die or withdraw before this time), or when the required number of events from the above sample size calculation has been reached in the ABC sub-group (123), whichever occurs first.

15.4 INITIAL PLAN OF ANALYSIS:

The trial will be analysed and reported according to the Consolidated Standard of Reporting Trials guidelines. A full statistical analysis plan will be written prior to the final analysis. The primary analysis will be based upon the intention-to-treat population.

Knowledge of the disease biology suggests that it may only be the ABC patients that will benefit from the addition of bortezomib to R-CHOP. Therefore two interim analyses will be performed, only in the GCB R-CHOP+B subgroup initially, first to determine if the addition of bortezomib is detrimental in GCB patients and second to gain an estimate of potential benefit.

A Case Morgan (Case and Morgan 2003) analysis will be applied to the GCB R-CHOP+B trial arm only. This analysis will be based upon 1 year PFS. With a significance level of 5% (two-sided) and 90% power, the analysis will be performed when 55 GCB R-CHOP+B patients have been recruited. If the PFS <70% the estimate suggests that the addition of bortezomib is detrimental in GCB patients, the GCB randomisation strata will be closed and all GCB patients will receive R-CHOP but will continue to be followed as per protocol.

A second Case Morgan analysis will be performed after 73 GCB patients treated with R- CHOP+B have been followed for 1 year. Data from Lenz et al (Lenz, et al 2008b) indicates an 85% I year PFS in GCB patients treated with R-CHOP chemotherapy (53% for ABC patients). If at this time point the 1 year PFS is at least 85% (significance 5% and power 90%) this would provide an estimate of benefit from the addition of bortezomib in GCB patients and they will continue to be randomised according to the schema above. If however the PFS is <85% at the time of this analysis it will be considered that the addition of bortezomib is unlikely to be effective and this stratum will therefore be closed and all GCB patients will receive R-CHOP.

In the event that there is no evidence that the addition of bortezomib to R-CHOP may be improving efficacy, the GCB strata will be closed. In this event the final analysis will be restricted to the ABC group in which the biological rationale is strongest.

PFS will be estimated using the method of Kaplan and Meier (Kaplan and Meier 1958) and will be compared across treatment and molecular groups using the log rank test with a two sided significance level of 0.05. The unadjusted hazard ratio (and confidence intervals) will be calculated using the Cox proportional hazards regression model. Secondary analyses will be performed for other important prognostic factors that may emerge throughout the course of the trial.

The differences in proportions of adverse events between treatment groups will be presented. Longitudinal analysis will be used to assess quality of life over time and treatment groups. The proportions of patients that experience CR, CRu and PR will be presented separately for each treatment and molecular group.

16 REGULATORY ISSUES

16.1 CLINICAL TRIAL AUTHORISATION

This trial has a Clinical Trial Authorisation from the UK Competent Authority the MHRA.

16.2 ETHICS APPROVAL

The trial protocol has received the favourable opinion of a main Research Ethics Committee or Institutional Review Board (IRB) in the approved international participating countries (UK and Switzerland).

The trial will be conducted in accordance with the recommendations for physicians involved in research on human patients adopted by the 18th World Medical Assembly, Helsinki 1964 as revised and recognised by governing laws and EU Directives. Each patient's consent to participate in the trial should be obtained after a full explanation has been given of treatment options, including the conventional and generally accepted methods of treatment. The right of the patient to refuse to participate in the trial without giving reasons must be respected.

After the patient has entered the trial, the clinician may give alternative treatment to that specified in the protocol, at any stage, if they feel it to be in the best interest of the patient. However, reasons for doing so should be recorded and the patient will remain within the trial for the purpose of follow-up and data analysis according to the treatment option to which they have been allocated. Similarly, the patient remains free to withdraw at any time from protocol treatment and trial follow-up without giving reasons and without prejudicing their further treatment.

The investigator must ensure that patient's anonymity will be maintained and that their identities are protected from unauthorised parties. On CRFs patients will not be identified by their names, but by an identification code. The investigator should keep a patient enrolment log showing codes, names and addresses.

16.3 CONSENT

Consent to enter the trial must be sought from each patient only after a full explanation has been given, an information leaflet offered and time allowed for consideration. Signed patient consent should be obtained. The right of the patient to refuse to participate without giving reasons must be respected. After the patient

has entered the trial the clinician remains free to give alternative treatment to that specified in the protocol at any stage if he/she feels it is in the patient's best interest, but the reasons for doing so should be recorded. In these cases the patients remain within the trial for the purposes of follow-up and data analysis. All patients are free to withdraw at any time from the protocol treatment without giving reasons and without prejudicing further treatment.

16.4 CONFIDENTIALITY

Patients' identification data will be required for the registration process. The SCTU will preserve the confidentiality of patients taking part in the trial.

16.5 INDEMNITY

The sponsor of the trial is University Hospital Southampton NHS Foundation Trust. For NHS sponsored research HSG (96) 48 reference no.2 refers. If there is negligent harm during the clinical trial when the NHS body owes a duty of care to the person harmed, NHS Indemnity covers NHS staff, medical academic staff with honorary contracts, and those conducting the trial. NHS Indemnity does not offer no-fault compensation and is unable to agree in advance to pay compensation for non-negligent harm. Ex-gratia payments may be considered in the case of a claim.

16.6 SPONSOR

University Hospital Southampton NHS Foundation Trust, Southampton is acting as the main sponsor for this trial. The SCTU has been delegated duties by the Sponsor relating to: submissions to regulatory authorities, GCP and pharmacovigilance. Other delegated duties will be assigned to the NHS Trusts or others taking part in this trial by means of the site clinical trial agreement.

16.7 FUNDING

This study is funded by an unrestricted educational grant from Janssen-Cilag. It has received CTAAC endorsement.

16.8 AUDITS AND INSPECTIONS

The trial may be patient to inspection and audit by University Hospital Southampton NHS Foundation Trust, Southampton, under their remit as sponsor, the SCTU as the Sponsor's delegate and other regulatory bodies to ensure adherence to ICH GCP, Research Governance Framework for Health and Social Care, applicable contracts/agreements and national regulations.

17 CASE REPORT FORMS AND MONITORING

17.1 COMPLETION OF THE CRF

Data will be collected and retained in accordance with the Data Protection Act (1988). Case report forms (CRFs) will be used to collect the data. The Investigator is responsible for ensuring the accuracy, completeness, legibility and timelines of the data reported in the CRFs. Study documents will be retained in a secure location during and after the trial has finished.

The CRFs must be completed in BLACK ink. Only the Investigator and those personnel authorised by him should enter or change data on the CRFs. All laboratory data and Investigator observations must be transcribed into the CRF where required.

Corrections can be made only by striking out any errors, with a single stroke, and not by using correction fluid. The correct entry must be entered by the side. The incorrect figure must remain visible and the correction should be initialled and dated by the person authorised by the Investigator to make the correction.

After all the queries have been resolved at the end of the study, the Investigator will confirm this by signing off the CRFs.

17.2 STUDY PERFORMANCE AND MONITORING

Before the study can be initiated, the prerequisites for conducting the study must be confirmed and the organisational preparations made with the trial centre. The suitability of the Investigator's research team, technical facilities and availability of eligible patients at the trial centre must be ensured. The Investigator must ensure that all study information is disseminated continuously to all those who are involved. The sponsor, via the Southampton Clinical Trials Unit (SCTU), must be informed immediately of any change in the persons involved in the conduct of the study at site.

The study will be monitored and audited in accordance with the Sponsor and SCTU/SAKK procedures. All trial-related documents will be made available on request for monitoring and audit by the Sponsor, SCTU/SAKK, the relevant ethics committee and for inspection by the MHRA or other relevant bodies. During the trial the Sponsor is responsible for monitoring data quality. Prior to the study start, the Investigator will be advised of the anticipated frequency of the monitoring visits. The Investigator will receive reasonable notification prior to each monitoring visit.

It is the duty of the Sponsor and SCTU/SAKK to review study records and compare them with source documents; discuss the conduct of the study and any emerging problems with the Investigator; check that the drug storage and dispensing are reliable and appropriate and verify that the available facilities remain acceptable.

At the final close-down visit, SCTU/SAKK will clarify any open questions, verify that all data requested and corrections have been entered correctly on the CRFs and collect the study material that is no longer required. All unused drug supplied will be destroyed as instructed by the SCTU and destruction certificates retained in the Investigator Site File.

17.3 SOURCE DOCUMENT VERIFICATION

The Investigator will allow the Sponsor and SCTU/SAKK direct access to relevant source documentation for verification of data entered onto the CRFs, taking into account data

protection regulations. Entries in the CRF will be compared with patients' medical records and the results will be documented on the Source Data Verification (SDV) Form and the monitoring report.

The patients' medical records, and other relevant data, may also be reviewed by appropriate qualified personnel, independent of UHS, appointed to audit the study, and by regulatory authorities. Details will remain confidential.

17.4 DATA MANAGEMENT

Completed CRFs should be submitted to the SCTU in a timely manner and will be reviewed by the data officer. Due to the planned interim analyses that are fundamental to the trial design, a request for CRF submission may on occasions be to a shorter timeline. Any queries raised on the submitted data will be sent to the site. The answered queries will be returned to the Southampton Clinical Trials Unit. This cycle may be repeated.

Data will be submitted to the following address:

REMoDL-B Data Officer
Southampton Clinical Trials Unit MP131
Southampton General Hospital Tremona Road
Southampton
Hampshire
SO16 6YD

17.5 SAFETY REPORTS

Safety reports will be generated by the SCTU which allow the monitoring of SAEs across sites. These will be reviewed by the clinical reviewers and by the Trial Management Group (TMG). There will also be independent oversight of safety events from the Trial Steering Committee (TSC) and the Data Monitoring and Ethics Committee (DMEC). The SCTU will prepare Annual Safety Reports for the MHRA and REC.

17.6 RECRUITMENT

The REMoDL-B Coordinator will produce monthly recruitment reports to allow the clinical reviewers to regularly review recruitment across sites.

17.7 PROTOCOL VIOLATIONS/DEVIATIONS

All protocol violations and deviations will be recorded, and reported to the Chief Investigator immediately. These will also be sent for review to the TMG.

17.8 STUDY REPORT

At appropriate intervals (annually), the Investigator is required to submit a study progress report to the main REC and the Competent Authority (MHRA).

17.9 RECORD RETENTION (ARCHIVING)

The Investigator must maintain adequate and accurate records to enable the conduct of the study to be fully documented and the study data to be subsequently verified. After study closure, the Investigator will maintain all source documents, study-related documents and

copies of the CRFs, Data Query and Amendment Forms. The Chief Investigator will retain specific study-related documents and the original CRFs. All source documents will be retained for a period of 15 years following the end of the study.

18 TRIAL MANAGEMENT

The Trial Management Group (TMG) is responsible for overseeing progress of the trial. The day-to-day management of the trial will be co-ordinated through the SCTU and oversight will be maintained by the Trial Steering Committee and the Data Monitoring and Ethics Committee (See Appendix 1).

19 PUBLICATION POLICY

Publication will follow the rules of the NCRI lymphoma CSG. Authorship will include the Chief Investigator, trial statistician, a representative of the SCTU, a member of the central histopathology team, and one additional author from each centre entering more than 5% of the total patients.

Individual Investigators must undertake not to submit any part of their individual data for publication without the prior approval of the TMG.

20 REFERENCES

Alizadeh, A.A., Eisen, M.B., Davis, R.E., Ma, C., Lossos, I.S., Rosenwald, A., Boldrick, J.C., Sabet, H., Tran, T., Yu, X., Powell, J.I., Yang, L., Marti, G.E., Moore, T., Hudson, J., Jr., Lu, L., Lewis, D.B., Tibshirani, R., Sherlock, G., Chan, W.C., Greiner, T.C., Weisenburger, D.D., Armitage, J.O., Warnke, R., Levy, R., Wilson, W., Grever, M.R., Byrd, J.C., Botstein, D., Brown, P.O. & Staudt, L.M. (2000) Distinct types of diffuse large B-cell lymphoma identified by gene expression profiling. *Nature,* **403,** 503-511.

Cancer Research UK (accessed August 2009) *UK Non-Hodgkin's Lymphoma Statistics,* *http://info.cancerresearchuk.org/cancerstats/types/nhl/?a=5441*.

Case, L.D. & Morgan, T.M. (2003) Design of Phase II cancer trials evaluating survival probabilities. *BMC Med Res Methodol,* **3,** 6.

Cheson, B.D., Horning, S.J., Coiffier, B., Shipp, M.A., Fisher, R.I., Connors, J.M., Lister, T.A., Vose, J., Grillo-Lopez, A., Hagenbeek, A., Cabanillas, F., Klippensten, D., Hiddemann, W., Castellino, R., Harris, N.L., Armitage, J.O., Carter, W., Hoppe, R. & Canellos, G.P. (1999) Report of an international workshop to standardize response criteria for non- Hodgkin's lymphomas. NCI Sponsored International Working Group. *J Clin Oncol,* **17,**
1244.

Cheson, B.D., Pfistner, B., Juweid, M.E., Gascoyne, R.D., Specht, L., Horning, S.J., Coiffier, B., Fisher, R.I., Hagenbeek, A., Zucca, E., Rosen, S.T., Stroobants, S., Lister, T.A., Hoppe, R.T., Dreyling, M., Tobinai, K., Vose, J.M., Connors, J.M., Federico, M. & Diehl, V. (2007) Revised response criteria for malignant lymphoma. *J Clin Oncol,* **25,** 579-586.

Choi, W.W., Weisenburger, D.D., Greiner, T.C., Piris, M.A., Banham, A.H., Delabie, J., Braziel, R.M., Geng, H., Iqbal, J., Lenz, G., Vose, J.M., Hans, C.P., Fu, K., Smith, L.M., Li, M., Liu, Z., Gascoyne, R.D., Rosenwald, A., Ott, G., Rimsza, L.M., Campo, E., Jaffe, E.S., Jaye, D.L., Staudt, L.M. & Chan, W.C. (2009) A new immunostain algorithm classifies diffuse large B-cell lymphoma into molecular subtypes with high accuracy. *Clin Cancer Res,* **15,**
5494-5502.

Coiffier, B., Lepage, E., Briere, J., Herbrecht, R., Tilly, H., Bouabdallah, R., Morel, P., Van Den Neste, E., Salles, G., Gaulard, P., Reyes, F., Lederlin, P. & Gisselbrecht, C. (2002) CHOP chemotherapy plus rituximab compared with CHOP alone in elderly patients with diffuse large-B-cell lymphoma. *N Engl J Med,* **346,** 235-242.

Compagno, M., Lim, W.K., Grunn, A., Nandula, S.V., Brahmachary, M., Shen, Q., Bertoni, F., Ponzoni, M., Scandurra, M., Califano, A., Bhagat, G., Chadburn, A., Dalla-Favera, R. & Pasqualucci, L. (2009) Mutations of multiple genes cause deregulation of NF-kappaB in diffuse large B-cell lymphoma. *Nature,* **459,** 717-721.

Cunningham, D., Smith, P., Mouncey, W., Qian, W., Pocock, C., Ardeshna, K., M., Radford, J., Davies, J., McMillan, A. & Linch, D. (2009) A phase III trial comparing R-CHOP 14 and R- CHOP 21 for the treatment of patients with newly diagnosed diffuse large B-cell non- Hodgkin's lymphoma. *J Clin Oncol,* **27,** abstr. 8506.

Davis, R.E., Brown, K.D., Siebenlist, U. & Staudt, L.M. (2001) Constitutive nuclear factor kappaB activity is required for survival of activated B cell-like diffuse large B cell lymphoma cells. *J Exp Med,* **194,** 1861-1874.

de Jong, D., Rosenwald, A., Chhanabhai, M., Gaulard, P., Klapper, W., Lee, A., Sander, B., Thorns, C., Campo, E., Molina, T., Norton, A., Hagenbeek, A., Horning, S., Lister, A., Raemaekers, J., Gascoyne, R.D., Salles, G. & Weller, E. (2007) Immunohistochemical prognostic markers in diffuse large B-cell lymphoma: validation of tissue microarray as a prerequisite for broad clinical applications--a study from the Lunenburg Lymphoma Biomarker Consortium. *J Clin Oncol,* **25,** 805-812.

Delarue, R., Tilly, H., Salles, G., Gisselbrecht, C., Mounier, N., Fournierm, M., Molina, T., Bonmati, C., Ghesquireres, H., Blanc, M., Germain, M., Girard, L., Haioun, C. & Bosly, A. (2009) R-CHOP14 Compared to R-CHOP21 in Elderly Patients with Diffuse Large B-Cell Lymphoma: Results of the Interim Analysis of the LNH03-6B GELA Study. *Blood,* **114,** abstr. 406.

Dunleavy, K., Pittaluga, S., Czuczman, M.S., Dave, S.S., Wright, G., Grant, N., Shovlin, M., Jaffe, E.S., Janik, J.E., Staudt, L.M. & Wilson, W.H. (2009) Differential efficacy of bortezomib plus chemotherapy within molecular subtypes of diffuse large B-cell lymphoma. *Blood,* **113,** 6069-6076.

Fisher, R.I., Gaynor, E.R., Dahlberg, S., Oken, M.M., Grogan, T.M., Mize, E.M., Glick, J.H., Coltman, C.A., Jr. & Miller, T.P. (1993) Comparison of a standard regimen (CHOP) with three intensive chemotherapy regimens for advanced non-Hodgkin's lymphoma. *N Engl J Med,* **328,** 1002-1006.

Gilmore, T.D., Kalaitzidis, D., Liang, M.C. & Starczynowski, D.T. (2004) The c-Rel transcription factor and B-cell proliferation: a deal with the devil. *Oncogene,* **23,** 2275-2286.

Gisselbrecht, C., Glass, B., Mounier, N., Gill, D., Linch, D., Trneny, M., Bosly, A., Shpilberg, O., Ketterer, N., Moskowitz, C. & Schmitz, N. (2009) R-ICE versus R-DHAP in relapsed patients with CD20 diffuse large B-cell lymphoma (DLBCL) followed by autologous stem cell transplantation: CORAL study. *J Clin Oncol,* **27,** abstr. 8509.

Goy, A., Younes, A., McLaughlin, P., Pro, B., Romaguera, J.E., Hagemeister, F., Fayad, L., Dang, N.H., Samaniego, F., Wang, M., Broglio, K., Samuels, B., Gilles, F., Sarris, A.H., Hart, S., Trehu, E., Schenkein, D., Cabanillas, F. & Rodriguez, A.M. (2005) Phase II Study of Proteasome Inhibitor Bortezomib in Relapsed or Refractory B-Cell Non-Hodgkin's Lymphoma. *J Clin Oncol,* **23,** 667-675.

Habermann, T.M., Weller, E.A., Morrison, V.A., Gascoyne, R.D., Cassileth, P.A., Cohn, J.B., Dakhil, S.R., Woda, B., Fisher, R.I., Peterson, B.A. & Horning, S.J. (2006) Rituximab- CHOP versus CHOP alone or with maintenance rituximab in older patients with diffuse large B-cell lymphoma. *J Clin Oncol,* **24,** 3121-3127.

Hans, C.P., Weisenburger, D.D., Greiner, T.C., Gascoyne, R.D., Delabie, J., Ott, G., Muller- Hermelink, H.K., Campo, E., Braziel, R.M., Jaffe, E.S., Pan, Z., Farinha, P., Smith, L.M., Falini, B., Banham, A.H., Rosenwald, A., Staudt, L.M., Connors, J.M., Armitage, J.O. & Chan, W.C. (2004) Confirmation of the molecular classification of diffuse large B-cell lymphoma by immunohistochemistry using a tissue microarray. *Blood,* **103,** 275-282.

Kaplan, E.L. & Meier, P. (1958) Nonparametric Estimation from Incomplete Observations. *Journal of the American Statistical Association,* **53,** 457-481.

Kim, J.E., Lee, D.H., Lee, S.I., Lee, J.H., Kim, W.S., Yoon, B. & Suh, C. (2009) Interim Report of Phase II Study of Bortezomib Plus CHOP Every 2 Weeks in Patients with Disseminated

Stage Diffuse Large B-Cell Lymphoma. *Blood (ASH Annual Meeting Abstracts),* **114,** 2688-.

Lenz, G., Davis, R.E., Ngo, V.N., Lam, L., George, T.C., Wright, G.W., Dave, S.S., Zhao, H., Xu, W., Rosenwald, A., Ott, G., Muller-Hermelink, H.K., Gascoyne, R.D., Connors, J.M., Rimsza, L.M., Campo, E., Jaffe, E.S., Delabie, J., Smeland, E.B., Fisher, R.I., Chan, W.C. & Staudt, L.M. (2008a) Oncogenic CARD11 mutations in human diffuse large B cell lymphoma. *Science,* **319,** 1676-1679.

Lenz, G., Wright, G., Dave, S.S., Xiao, W., Powell, J., Zhao, H., Xu, W., Tan, B., Goldschmidt, N., Iqbal, J., Vose, J., Bast, M., Fu, K., Weisenburger, D.D., Greiner, T.C., Armitage, J.O., Kyle, A., May, L., Gascoyne, R.D., Connors, J.M., Troen, G., Holte, H., Kvaloy, S., Dierickx, D., Verhoef, G., Delabie, J., Smeland, E.B., Jares, P., Martinez, A., Lopez- Guillermo, A., Montserrat, E., Campo, E., Braziel, R.M., Miller, T.P., Rimsza, L.M., Cook, J.R., Pohlman, B., Sweetenham, J., Tubbs, R.R., Fisher, R.I., Hartmann, E., Rosenwald, A., Ott, G., Muller-Hermelink, H.K., Wrench, D., Lister, T.A., Jaffe, E.S., Wilson, W.H., Chan, W.C. & Staudt, L.M. (2008b) Stromal gene signatures in large-B-cell lymphomas. *N Engl J Med,* **359,** 2313-2323.

Lenz, G., Wright, G.W., Emre, N.C., Kohlhammer, H., Dave, S.S., Davis, R.E., Carty, S., Lam, L.T., Shaffer, A.L., Xiao, W., Powell, J., Rosenwald, A., Ott, G., Muller-Hermelink, H.K., Gascoyne, R.D., Connors, J.M., Campo, E., Jaffe, E.S., Delabie, J., Smeland, E.B., Rimsza, L.M., Fisher, R.I., Weisenburger, D.D., Chan, W.C. & Staudt, L.M. (2008c) Molecular subtypes of diffuse large B-cell lymphoma arise by distinct genetic pathways. *Proc Natl Acad Sci U S A,* **105,** 13520-13525.

Leonard, J.P., Furman, R.R., Cheung, Y.K., Vose, J.M., Glynn, P.W., Ruan, J., Martin, P., Niesvizky, R., LaCasce, A., Chadburn, A. & Coleman, M. (2007) CHOP-R + bortezomib as initial therapy for diffuse large B-cell lymphoma (DLBCL). *J Clin Oncol (Meeting Abstracts),* **25,** 8031-.

Moreau P, Pylypenko H, Grosicki S (2011) Subcutaneous versus intravenous administration of botesomib in patients with relapsed multiple myeloma: a randomized, phase 3, non-inferiority study. *LanceatOncol. 12 (5): 431-41*

Mounier, N., Ribrag, V., Haioun, C., Salles, G., Golfier, M., Ertault, M., Ferme, C., Briere, J., Brice, P., De Kerviler, E. & Gisselbrecht, C. (2007 (abstr.)) Efficacy and toxicity of two schedules of R-CHOP plus bortezomib in front-line B lymphoma patients: A randomized phase II trial from the Groupe d'Etude des Lymphomes de l'Adulte (GELA). *J Clin Oncol,* **25,** 8010.

Nencioni, A., Grunebach, F., Patrone, F., Ballestrero, A. & Brossart, P. (2007) Proteasome inhibitors: antitumor effects and beyond. *Leukemia,* **21,** 30-36.

Ngo, V.N., Davis, R.E., Lamy, L., Yu, X., Zhao, H., Lenz, G., Lam, L.T., Dave, S., Yang, L., Powell, J. & Staudt, L.M. (2006) A loss-of-function RNA interference screen for molecular targets in cancer. *Nature,* **441,** 106-110.

Oken, M.M., Creech, R.H., Tormey, D.C., Horton, J., Davis, T.E., McFadden, E.T. & Carbone, P.P.(1982) Toxicity And Response Criteria Of The Eastern Cooperative Oncology Group. *Am J Clin Oncol,* ***5****, 649-655.*

Packham, G. (2008) The role of NF-kappaB in lymphoid malignancies. *Br J Haematol,* **143,** 3-15.

Pfreundschuh, M., Schubert, J., Ziepert, M., Schmits, R., Mohren, M., Lengfelder, E., Reiser, M., Nickenig, C., Clemens, M., Peter, N., Bokemeyer, C., Eimermacher, H., Ho, A., Hoffmann, M., Mertelsmann, R., Trumper, L., Balleisen, L., Liersch, R., Metzner, B., Hartmann, F., Glass, B., Poeschel, V., Schmitz, N., Ruebe, C., Feller, A.C. & Loeffler, M. (2008) Six versus eight cycles of bi-weekly CHOP-14 with or without rituximab in elderly patients with aggressive CD20+ B-cell lymphomas: a randomised controlled trial (RICOVER-60). *Lancet Oncol,* **9,** 105-116.

Pfreundschuh, M., Trumper, L., Kloess, M., Schmits, R., Feller, A.C., Rube, C., Rudolph, C., Reiser, M., Hossfeld, D.K., Eimermacher, H., Hasenclever, D., Schmitz, N. & Loeffler, M. (2004a) Two-weekly or 3-weekly CHOP chemotherapy with or without etoposide for the treatment of elderly

patients with aggressive lymphomas: results of the NHL-B2 trial of the DSHNHL. *Blood,* **104,** 634-641.

Pfreundschuh, M., Trumper, L., Kloess, M., Schmits, R., Feller, A.C., Rudolph, C., Reiser, M., Hossfeld, D.K., Metzner, B., Hasenclever, D., Schmitz, N., Glass, B., Rube, C. & Loeffler, M. (2004b) Two-weekly or 3-weekly CHOP chemotherapy with or without etoposide for the treatment of young patients with good-prognosis (normal LDH) aggressive lymphomas: results of the NHL-B1 trial of the DSHNHL. *Blood,* **104,** 626-633.

Pfreundschuh, M., Trumper, L., Osterborg, A., Pettengell, R., Trneny, M., Imrie, K., Ma, D., Gill, D., Walewski, J., Zinzani, P.L., Stahel, R., Kvaloy, S., Shpilberg, O., Jaeger, U., Hansen, M., Lehtinen, T., Lopez-Guillermo, A., Corrado, C., Scheliga, A., Milpied, N., Mendila, M., Rashford, M., Kuhnt, E. & Loeffler, M. (2006) CHOP-like chemotherapy plus rituximab versus CHOP-like chemotherapy alone in young patients with good- prognosis diffuse large-B-cell lymphoma: a randomised controlled trial by the MabThera International Trial (MInT) Group. *Lancet Oncol,* **7,** 379-391.

Rimsza, L., Wright, G., Schwartz, M., Chan, W.C., Jaffe, E.S., Gascoyne, R.D., Campo, E., Rosenwald, A., Ott, G., Cook, J.R., Tubbs, R.R., Braziel, R., Delabie, J., Miller, T.P., Fisher, R.I. & Staudt, L.M. (2009) Accurate Classification of Diffuse Large B Cell Lymphoma Into Germinal Center and Activated B Cell Subtypes Using a Nuclease Protection Assay On Formalin Fixed Paraffin Embedded Tissue: A Study From the Lymphoma and Leukemia Molecular Profiling Project *Blood,* **114,** abstr. 620.

Rosenwald, A., Wright, G., Chan, W.C., Connors, J.M., Campo, E., Fisher, R.I., Gascoyne, R.D., Muller-Hermelink, H.K., Smeland, E.B., Giltnane, J.M., Hurt, E.M., Zhao, H., Averett, L., Yang, L., Wilson, W.H., Jaffe, E.S., Simon, R., Klausner, R.D., Powell, J., Duffey, P.L., Longo, D.L., Greiner, T.C., Weisenburger, D.D., Sanger, W.G., Dave, B.J., Lynch, J.C., Vose, J., Armitage, J.O., Montserrat, E., Lopez-Guillermo, A., Grogan, T.M., Miller, T.P., LeBlanc, M., Ott, G., Kvaloy, S., Delabie, J., Holte, H., Krajci, P., Stokke, T. & Staudt, L.M. (2002) The use of molecular profiling to predict survival after chemotherapy for diffuse large-B-cell lymphoma. *N Engl J Med,* **346,** 1937-1947.

Ruan, J., Martin, P., Furman, R.R., Vose, J.M., LaCasce, A., Cheung, Y.-K.K., O'Loughlin, J., Elstrom, R., Niesvizky, R., Chadburn, A., Ely, S., Cesarman, E., Coleman, M. & Leonard, J.P. (2009) CHOP-R + Bortezomib as Initial Therapy for Mantle Cell Lymphoma (MCL). *Blood (ASH Annual Meeting Abstracts),* **114,** 2682-.

Sehn, L.H., Donaldson, J., Chhanabhai, M., Fitzgerald, C., Gill, K., Klasa, R., MacPherson, N., O'Reilly, S., Spinelli, J.J., Sutherland, J., Wilson, K.S., Gascoyne, R.D. & Connors, J.M. (2005) Introduction of combined CHOP plus rituximab therapy dramatically improved outcome of diffuse large B-cell lymphoma in British Columbia. *J Clin Oncol,* **23,** 5027-5033.

Sehn, L.H., Macdonald, D.A., Rubin, S.H., Cantin, G., Rubinger, M., Lemieux, B., Basi, S., Imrie, K.R., Gascoyne, R.D., Chen, B., Djurfeldt, M., Shepherd, L., Couban, S. & Crump, M. (2009) Bortezomib Added to CVP-R Is Safe and Effective for Previously Untreated Advanced Stage Follicular Lymphoma: A Phase II Study by the NCIC Clinical Trials Group. *ASH Annual Meeting Abstracts,* **114,** 407-.

Simon, R. (2008) The use of genomics in clinical trial design. *Clin Cancer Res,* **14,** 5984-5993.

Sinha, R., Kaufman, J.L., Khoury, H.J., Jr., King, N., Shenoy, P.J., Lewis, C., Bumpers, K., Lonial, S. & Flowers, C.R. (2009) The Addition of Bortezomib to Modified R-CHOP as Initial Therapy for Follicular NHL Results in High CR Rate and Is Well Tolerated. *Blood (ASH Annual Meeting Abstracts),* **114,** 3718-.

Strauss, S.J., Maharaj, L., Hoare, S., Johnson, P.W., Radford, J.A., Vinnecombe, S., Millard, L., Rohatiner, A., Boral, A., Trehu, E., Schenkein, D., Balkwill, F., Joel, S.P. & Lister, T.A. (2006) Bortezomib therapy in patients with relapsed or refractory lymphoma: potential correlation of in vitro sensitivity and tumor necrosis factor alpha response with clinical activity. *J Clin Oncol,* **24,** 2105-2112.

The International Non-Hodgkin's Lymphoma Prognostic Factors Project (1993) A predictive model for aggressive non-Hodgkin's lymphoma. *N Engl J Med,* **329,** 987-994.

The Non-Hodgkin's Lymphoma Classification Project (1997) A clinical evaluation of the International Lymphoma Study Group classification of non-Hodgkin's lymphoma. *Blood,* **89,** 3909-3918.

Venkatakrishnan, K., Rader, M., Ramanathan, R.K., Ramalingam, S., Chen, E., Riordan, W., Trepicchio, W., Cooper, M., Karol, M., von Moltke, L., Neuwirth, R., Egorin, M. & Chatta, G. (2009) Effect of the CYP3A inhibitor ketoconazole on the pharmacokinetics and pharmacodynamics of bortezomib in patients with advanced solid tumors: a prospective, multicenter, open-label, randomized, two-way crossover drug-drug interaction study. *Clin Ther,* **31 Pt 2,** 2444-2458.

Wright, G., Tan, B., Rosenwald, A., Hurt, E.H., Wiestner, A. & Staudt, L.M. (2003) A gene expression-based method to diagnose clinically distinct subgroups of diffuse large B cell lymphoma. *Proc Natl Acad Sci U S A,* **100,** 9991-9996.

APPENDIX 1 COMMUNICATION AND RELATIONSHIP BETWEEN PARTIES

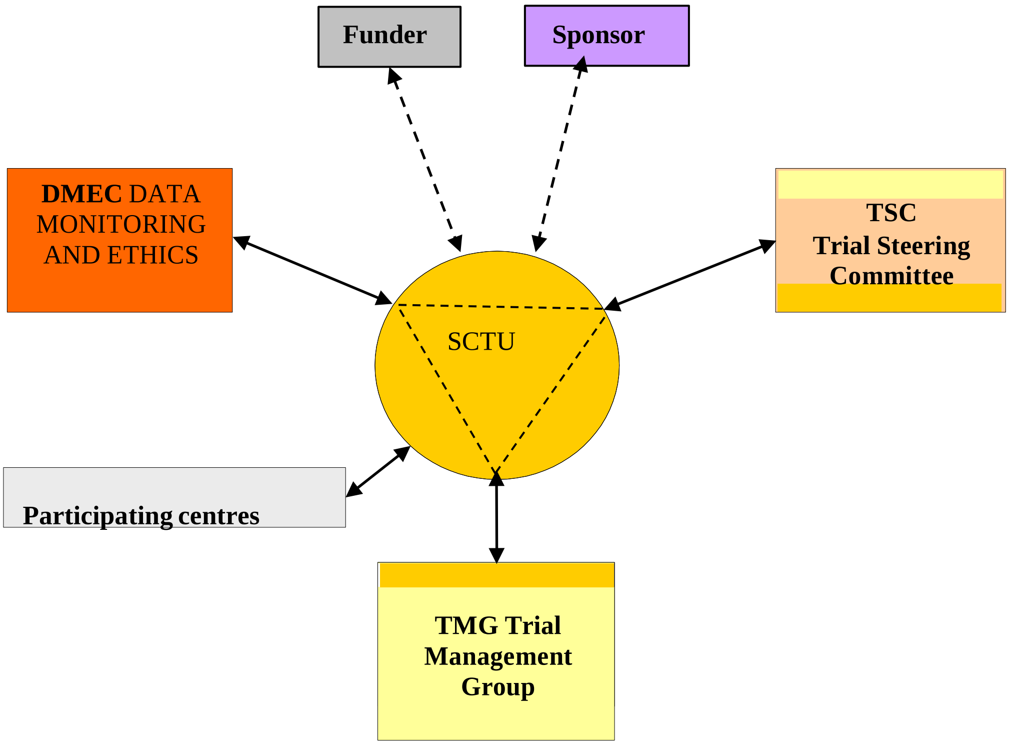

APPENDIX 2 ECOG* PERFORMANCE STATUS

GRADE	PERFORMANCE STATUS
0	Able to carry out all normal activity without restriction.
1	Restricted in physically strenuous activity but ambulatory and able to carry out light work.
2	Ambulatory and capable of all self-care but unable to carry out any work; up and about more 50% of waking hours.
3	Capable of only limited self-care confined to bed or chair more than 50% of waking hours.
4	Completely disabled; cannot carry out any self-care; totally confined to bed and chair.

* Eastern Cooperative Oncology Group, Robert Comis M.D., Group Chair, 1982, (Oken, et al, 1982)

APPENDIX 3 INTERNATIONAL PROGNOSTIC INDEX

The adverse factors used to calculate the International Prognostic Index (IPI) are listed below (The International Non-Hodgkin's Lymphoma Prognostic Factors Project 1993). Score one point for each criteria met.

Adverse Factors

Parameter	Adverse Factor
Age	>60 years
Ann Arbor stage	III-IV
ECOG performance status	>1
Serum LDH level	> ULN
Number of extranodal sites	> 1

Patients are assigned to one of four risk groups based upon the number of presenting risk factors:

Risk Group	Score
Low	0 or 1
Low Intermediate	2
High Intermediate	3
High	4 or 5

APPENDIX 4 COMMON TERMINOLOGY CRITERIA FOR ADVERSE EVENTS

The Common Toxicity Criteria has now become the Common Terminology Criteria for Adverse Event Reporting. Version 4.0 will be used in REMoDL-B. A full version of this document can be downloaded in pdf format from the NCI website:

http://evs.nci.nih.gov/ftp1/CTCAE/CTCAE_4.03_2010-06-14_QuickReference_8.5x11.pdf

APPENDIX 5 QUALITY OF LIFE QUESTIONNAIRES

EORTC QLQ-C30 with CIPN20 module

ENGLISH

EORTC QLQ-C30 (version 3)

We are interested in some things about you and your health. Please answer all of the questions yourself by circling the number that best applies to you. There are no "right" or "wrong" answers. The information that you provide will remain strictly confidential.

Please fill in your initials: |_|_|_|_|
Your birthdate (Day, Month, Year): |_|_|_|_|_|_|_|_|
Today's date (Day, Month, Year): 31 |_|_|_|_|_|_|_|_|

	Not at All	A Little	Quite a Bit	Very Much
1. Do you have any trouble doing strenuous activities, like carrying a heavy shopping bag or a suitcase?	1	2	3	4
2. Do you have any trouble taking a long walk?	1	2	3	4
3. Do you have any trouble taking a short walk outside of the house?	1	2	3	4
4. Do you need to stay in bed or a chair during the day?	1	2	3	4
5. Do you need help with eating, dressing, washing yourself or using the toilet?	1	2	3	4

**During the past week:**	Not at All	A Little	Quite a Bit	Very Much
6. Were you limited in doing either your work or other daily activities?	1	2	3	4
7. Were you limited in pursuing your hobbies or other leisure time activities?	1	2	3	4
8. Were you short of breath?	1	2	3	4
9. Have you had pain?	1	2	3	4
10. Did you need to rest?	1	2	3	4
11. Have you had trouble sleeping?	1	2	3	4
12. Have you felt weak?	1	2	3	4
13. Have you lacked appetite?	1	2	3	4
14. Have you felt nauseated?	1	2	3	4
15. Have you vomited?	1	2	3	4
16. Have you been constipated?	1	2	3	4

Please go on to the next page

**During the past week:**	**Not at All**	**A Little**	**Quite a Bit**	**Very Much**
17. Have you had diarrhea?	1	2	3	4
18. Were you tired?	1	2	3	4
19. Did pain interfere with your daily activities?	1	2	3	4
20. Have you had difficulty in concentrating on things, like reading a newspaper or watching television?	1	2	3	4
21. Did you feel tense?	1	2	3	4
22. Did you worry?	1	2	3	4
23. Did you feel irritable?	1	2	3	4
24. Did you feel depressed?	1	2	3	4
25. Have you had difficulty remembering things?	1	2	3	4
26. Has your physical condition or medical treatment interfered with your family life?	1	2	3	4
27. Has your physical condition or medical treatment interfered with your social activities?	1	2	3	4
28. Has your physical condition or medical treatment caused you financial difficulties?	1	2	3	4

For the following questions please circle the number between 1 and 7 that best applies to you

29. How would you rate your overall health during the past week?

1	2	3	4	5	6	7
Very poor						Excellent

30. How would you rate your overall quality of life during the past week?

1	2	3	4	5	6	7
Very poor						Excellent

© Copyright 1995 EORTC Quality of Life Group. All rights reserved. Version 3.0

EORTC QLQ – CIPN20

Patients sometimes report that they have the following symptoms or problems. Please indicate the extent to which you have experienced these symptoms or problems during the past week. Please answer by circling the number that best applies to you.

During the past week :	Not at All	A Little	Quite a Bit	Very Much
31 Did you have tingling fingers or hands?	1	2	3	4
32 Did you have tingling toes or feet?	1	2	3	4
33 Did you have numbness in your fingers or hands?	1	2	3	4
34 Did you have numbness in your toes or feet?	1	2	3	4
35 Did you have shooting or burning pain in your fingers or hands?	1	2	3	4
36 Did you have shooting or burning pain in your toes or feet?	1	2	3	4
37 Did you have cramps in your hands?	1	2	3	4
38 Did you have cramps in your feet?	1	2	3	4
39 Did you have problems standing or walking because of difficulty feeling the ground under your feet?	1	2	3	4
40 Did you have difficulty distinguishing between hot and cold water?	1	2	3	4
41 Did you have a problem holding a pen, which made writing difficult?	1	2	3	4
42 Did you have difficulty manipulating small objects with your fingers (for example, fastening small buttons)?	1	2	3	4
43 Did you have difficulty opening a jar or bottle because of weakness in your hands?	1	2	3	4
44 Did you have difficulty walking because your feet dropped downwards?	1	2	3	4

Please go on to the next page

During the past week :	Not at All	A Little	Quite a Bit	Very Much
45 Did you have difficulty climbing stairs or getting up out of a chair because of weakness in your legs?	1	2	3	4
46 Were you dizzy when standing up from a sitting or lying position?	1	2	3	4
47 Did you have blurred vision?	1	2	3	4
48 Did you have difficulty hearing?	1	2	3	4
**Please answer the following question only if you drive a car**				
49 Did you have difficulty using the pedals?	1	2	3	4
**Please answer the following question only if you are a man**				
50 Did you have difficulty getting or maintaining an erection?	1	2	3	4

APPENDIX 6 RANDOMISATION FLOW CHART GUIDE

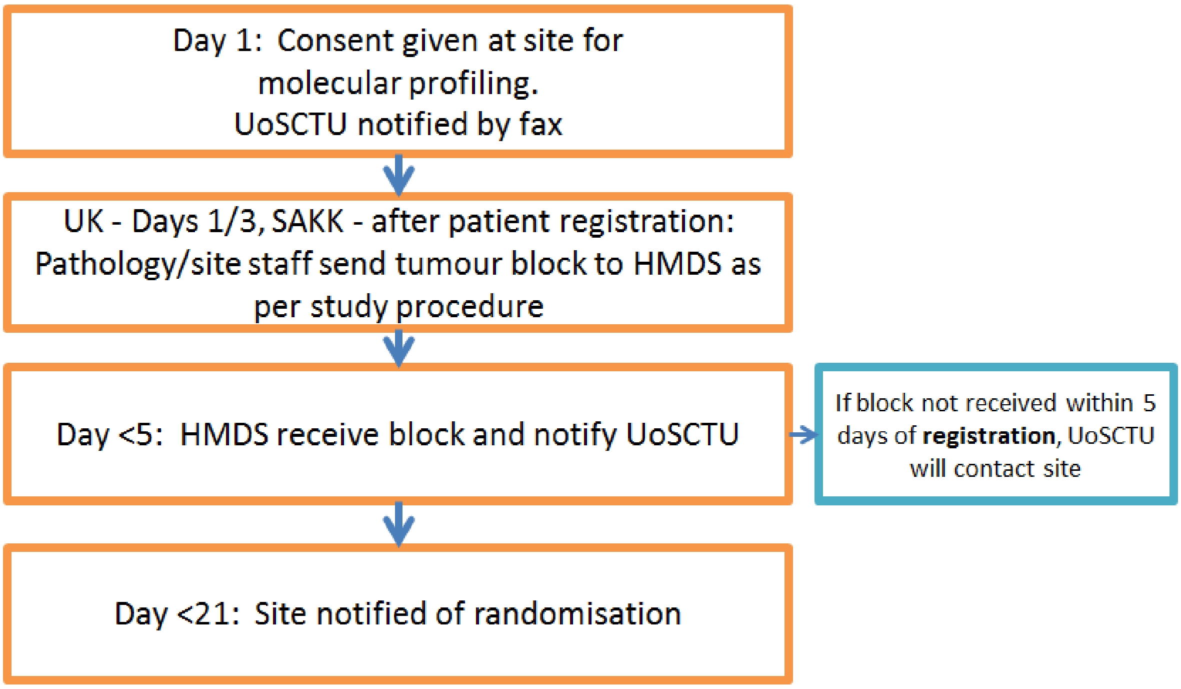

Instructions for tissue sample collection and shipment will be sent to sites before initiation in the Investigator Site File.

APPENDIX 7 EXAMPLE LIST OF EXPECTED TOXICITIES

Expected adverse events are recorded in the summary of product characteristics (SmPCs) for each investigation medicinal product (IMP). In REMoDL-B the following are the IMPs:

Rituximab
Cyclophosphamide
Doxorubicin
Vincristine
Prednisolone
Bortezomib

The table below states which SmPC/IB version is being used as the reference safety information for this trial. Please check the date of revision of the text prior to assessing an event.

Name of Product	IB/SmPC	Manufacturer	Date of text revision
Bortezomib	IB 18	Janssen	03-Jun-2015
Rituximab	SmPC	Roche Products Limited	26-May-2016
Cyclophosphamide	SmPC	Baxter Healthcare Ltd	07-Jun-2016
Doxorubicin	SmPC	Pfizer Limited	Feb 2016
Vincristine	SmPC	Hospira UK Ltd	April 2016
Prednisolone	SmPC	Alliance Pharmaceuticals Limited	13-Jul-2016

The PI at each site is responsible for obtaining the latest SmPC for each IMP. Where there are more than one manufacturer of an IMP the PI is responsible for ascertaining which is used by their site pharmacy and placing a copy in the Investigator Site File.

APPENDIX 8 CYP3A4 INHIBITORS AND

INDUCERS <u>CYP3A4 Inhibitors and Bortezomib</u>

An interaction study based on data from 12 patients, assessing the effect of ketoconazole, a potent CYP3A4 inhibitor, showed a bortezomib AUC mean increase of 35% (Venkatakrishnan, *et al* 2009). Therefore patients should be closely monitored when given bortezomib in combination with potent CYP3A4 inhibitors.

Other inhibitors to consider using with caution during co-treatment with bortezomib are listed below. This list is not exhaustive. For further information see: http://medicine.iupui.edu/clinpharm/ddis/table.asp

+++ strong inhibitor
++ moderate inhibitor
+ weak inhibitor

HIV protease Inhibitors
Indinavir (+++)
nelfinavir (+++)
ritonavir (+++)
saquinavir (+++)

Macrolide antibiotics
Clarithromycin (+++)
telithromycin (+++)
Erythromycin (++)

Antifungals
itraconazole (+++)
ketoconazole (+++)

Calcium channel blockers
Diltiazem (++)
Verapamil (++)

Antidepressants
Nefazodone
Fluvoxamine

Others
grapefruit juice (++)
aprepitant (++)
cimetidine (+)

<u>CYP3A4 Inducers and Bortezomib</u> In the absence of interaction studies investigating the effect of CYP3A4 inducers on the pharmacokinetics of bortezomib, patients should be closely monitored when given bortezomib in combination with potent CYP3A4 inducers (e.g. efavirenez, phenytoin, rifampicin, and St John's wort).

APPENDIX 9 DECLARATION OF HELSINKI

The trial will be conducted in accordance with the recommendations for physicians involved in research on human Patients adopted by the 18th World Medical Assembly, Helsinki 1964 as revised and recognised by governing laws and EU Directives. Each patient's consent to participate in the trial should be obtained after a full explanation has been given of treatment options, including the conventional and generally accepted methods of treatment. The right of the patient to refuse to participate in the trial without giving reasons must be respected.

WORLD MEDICAL ASSOCIATION DECLARATION OF HELSINKI Ethical Principles for Medical Research Involving Human Patients

A. INTRODUCTION

1. The World Medical Association has developed the Declaration of Helsinki as a statement of ethical principles to provide guidance to physicians and other patients in medical research involving human patients. Medical research involving human patients includes research on identifiable human material or identifiable data.

2. It is the duty of the physician to promote and safeguard the health of the people. The physician's knowledge and conscience are dedicated to the fulfilment of this duty.

3. The Declaration of Geneva of the World Medical Association binds the physician with the words, "The health of my patient will be my first consideration," and the International Code of Medical Ethics declares that, "A physician shall act only in the patient's interest when providing medical care which might have the effect of weakening the physical and mental condition of the patient."

4. Medical progress is based on research which ultimately must rest in part on experimentation involving human patients.

5. In medical research on human patients, considerations related to the well-being of the human patient should take precedence over the interests of science and society.

6. The primary purpose of medical research involving human patients is to improve prophylactic, diagnostic and therapeutic procedures and the understanding of the aetiology and pathogenesis of disease. Even the best proven prophylactic, diagnostic, and therapeutic methods must continuously be challenged through research for their effectiveness, efficiency, accessibility and quality.

7. In current medical practice and in medical research, most prophylactic, diagnostic and therapeutic procedures involve risks and burdens.

8. Medical research is patient to ethical standards that promote respect for all human beings and protect their health and rights. Some research populations are vulnerable and need special protection. The particular needs of the economically and medically

disadvantaged must be recognised. Special attention is also required for those who cannot give or refuse consent for themselves, for those who may be patient to giving consent under duress, for those who will not benefit personally from the research and for those for whom the research is combined with care.

9. Research Investigators should be aware of the ethical, legal and regulatory requirements for research on human patients in their own countries as well as applicable international requirements. No national ethical, legal or regulatory requirement should be allowed to reduce or eliminate any of the protections for human patients set forth in this Declaration.

B. BASIC PRINCIPLES FOR ALL MEDICAL RESEARCH

10. It is the duty of the physician in medical research to protect the life, health, privacy, and dignity of the human patient.

11. Medical research involving human patients must conform to generally accepted scientific principles, be based on a thorough knowledge of the scientific literature, other relevant sources of information, and on adequate laboratory and, where appropriate, animal experimentation.

12. Appropriate caution must be exercised in the conduct of research which may affect the environment, and the welfare of animals used for research must be respected.

13. The design and performance of each experimental procedure involving human patients should be clearly formulated in an experimental protocol. This protocol should be submitted for consideration, comment, guidance, and where appropriate, approval to a specially appointed ethical review committee, which must be independent of the investigator, the funder or any other kind of undue influence. This independent committee should be in conformity with the laws and regulations of the country in which the research experiment is performed. The committee has the right to monitor ongoing trials. The researcher has the obligation to provide monitoring information to the committee, especially any serious adverse events. The researcher should also submit to the committee, for review, information regarding funding, sponsors, institutional affiliations, other potential conflicts of interest and incentives for patients.

14. The research protocol should always contain a statement of the ethical considerations involved and should indicate that there is compliance with the principles enunciated in this Declaration.

15. Medical research involving human patients should be conducted only by scientifically qualified persons and under the supervision of a clinically competent medical person. The responsibility for the human patient must always rest with a medically qualified person and never rest on the patient of the research, even though the patient has given consent.

16. Every medical research project involving human patients should be preceded by careful assessment of predictable risks and burdens in comparison with foreseeable benefits to the patient or to others. This does not preclude the participation of healthy volunteers in medical research. The design of all studies should be publicly available.

17. Physicians should abstain from engaging in research projects involving human patients unless they are confident that the risks involved have been adequately assessed and can be satisfactorily managed. Physicians should cease any investigation if the risks are found to outweigh the potential benefits or if there is conclusive proof of positive and beneficial results.

18. Medical research involving human patients should only be conducted if the importance of the objective outweighs the inherent risks and burdens to the patient. This is especially important when the human patients are healthy volunteers.

19. Medical research is only justified if there is a reasonable likelihood that the populations in which the research is carried out stand to benefit from the results of the research.

20. The patients must be volunteers and informed patients in the research project.

21. The right of research patients to safeguard their integrity must always be respected. Every precaution should be taken to respect the privacy of the patient, the confidentiality of the patient's information and to minimise the impact of the study on the patient's physical and mental integrity and on the personality of the patient.

22. In any research on human beings, each potential patient must be adequately informed of the aims, methods, sources of funding, any possible conflicts of interest, institutional affiliations of the researcher, the anticipated benefits and potential risks of the study and the discomfort it may entail. The patient should be informed of the right to abstain from participation in the study or to withdraw consent to participate at any time without reprisal. After ensuring that the patient has understood the information, the physician should then obtain the patient's freely-given informed consent, preferably in writing. If the consent cannot be obtained in writing, the non-written consent must be formally documented and witnessed.

23. When obtaining informed consent for the research project the physician should be particularly cautious if the patient is in a dependent relationship with the physician or may consent under duress. In that case the informed consent should be obtained by a well-informed physician who is not engaged in the investigation and who is completely independent of this relationship.

24. For a research patient who is legally incompetent, physically or mentally incapable of giving consent or is a legally incompetent minor, the investigator must obtain informed consent from the legally authorised representative in accordance with applicable law. These groups should not be included in research unless the research is necessary to promote the health of the population represented and this research cannot instead be performed on legally competent persons.

25. When a patient deemed legally incompetent, such as a minor child, is able to give assent to decisions about participation in research, the investigator must obtain that assent in addition to the consent of the legally authorised representative.

26. Research on individuals from whom it is not possible to obtain consent, including proxy or advance consent, should be done only if the physical/mental condition that prevents obtaining informed consent is a necessary characteristic of the research

population. The specific reasons for involving research patients with a condition that renders them unable to give informed consent should be stated in the experimental protocol for consideration and approval of the review committee. The protocol should state that consent to remain in the research should be obtained as soon as possible from the individual or a legally authorised surrogate.

27. Both authors and publishers have ethical obligations. In publication of the results of research, the investigators are obliged to preserve the accuracy of the results. Negative as well as positive results should be published or otherwise publicly available. Sources of funding, institutional affiliations and any possible conflicts of interest should be declared in the publication. Reports of experimentation not in accordance with the principles laid down in this Declaration should not be accepted for publication.

C. ADDITIONAL PRINCIPLES FOR MEDICAL RESEARCH COMBINED WITH MEDICAL CARE

28. The physician may combine medical research with medical care, only to the extent that the research is justified by its potential prophylactic, diagnostic or therapeutic value. When medical research is combined with medical care, additional standards apply to protect the patients who are research patients.

29. The benefits, risks, burdens and effectiveness of a new method should be tested against those of the best current prophylactic, diagnostic, and therapeutic methods. This does not exclude the use of placebo, or no treatment, in studies where no proven prophylactic, diagnostic or therapeutic method exists.

30. At the conclusion of the study, every patient entered into the study should be assured of access to the best proven prophylactic, diagnostic and therapeutic methods identified by the study.

31. The physician should fully inform the patient which aspects of the care are related to the research. The refusal of a patient to participate in a study must never interfere with the patient-physician relationship.

32. In the treatment of a patient, where proven prophylactic, diagnostic and therapeutic methods do not exist or have been ineffective, the physician, with informed consent from the patient, must be free to use unproven or new prophylactic, diagnostic and therapeutic measures, if in the physician's judgement it offers hope of saving life, re- establishing health or alleviating suffering. Where possible, these measures should be made the object of research, designed to evaluate their safety and efficacy. In all cases, new information should be recorded and, where appropriate, published. The other relevant guidelines of this Declaration should be followed.

Appendix - CONSORT Checklist

Section/topic	Item number	Checklist item	Reported on page number
**Title and abstract**			
	1a	Identification as a randomised trial in the title	2
	1b	Structured summary of trial design, methods, results, and conclusions (for specific guidance see CONSORT for abstracts[21,31])	2
**Introduction**			
Background and objectives	2a	Scientific background and explanation of rationale	3
	2b	Specific objectives or hypotheses	3
**Methods**			
Trial design	3a	Description of trial design (such as parallel, factorial) including allocation ratio	4
	3b	Important changes to methods after trial commencement (such as eligibility criteria), with reasons	5
Participants	4a	Eligibility criteria for participants	4
	4b	Settings and locations where the data were collected	4-6
Interventions	5	The interventions for each group with sufficient details to allow replication, including how and when they were actually administered	4-5
Outcomes	6a	Completely defined prespecified primary and secondary outcome measures, including how and when they were assessed	6
	6b	Any changes to trial outcomes after the trial commenced, with reasons	N/A
Sample size	7a	How sample size was determined	6
	7b	When applicable, explanation of any interim analyses and stopping guidelines	6
**Randomisation**			
Sequence generation	8a	Method used to generate the random allocation sequence	4
	8b	Type of randomisation; details of any restriction (such as blocking and block size)	4
Allocation concealment mechanism	9	Mechanism used to implement the random allocation sequence (such as sequentially numbered containers), describing any steps taken to conceal the sequence until interventions were assigned	4
Implementation	10	Who generated the random allocation sequence, who enrolled participants, and who assigned participants to interventions	4-5
Blinding	11a	If done, who was blinded after assignment to interventions (for example, participants, care providers, those assessing outcomes) and how	N/A
	11b	If relevant, description of the similarity of interventions	N/A
Statistical methods	12a	Statistical methods used to compare groups for primary and secondary outcomes	7
	12b	Methods for additional analyses, such as subgroup analyses and adjusted analyses	6-7
**Results**			
Participant flow (a diagram is strongly recommended)	13a	For each group, the numbers of participants who were randomly assigned, received intended treatment, and were analysed for the primary outcome	Figure 1
	13b	For each group, losses and exclusions after randomisation, together with reasons	Figure 1
Recruitment	14a	Dates defining the periods of recruitment and follow-up	7
	14b	Why the trial ended or was stopped	N/A
Baseline data	15	A table showing baseline demographic and clinical characteristics for each group	Table 1
Numbers analysed	16	For each group, number of participants (denominator) included in each analysis and whether the analysis was by original assigned groups	Figure 1
Outcomes and estimation	17a	For each primary and secondary outcome, results for each group, and the estimated effect size and its precision (such as 95% CI)	9
	17b	For binary outcomes, presentation of both absolute and relative effect sizes is recommended	N/A
Ancillary analyses	18	Results of any other analyses performed, including subgroup analyses and adjusted analyses, distinguishing prespecified from exploratory	9
Harms	19	All important harms or unintended effects in each group (for specific guidance see CONSORT for harms[28])	8-9
**Discussion**			
Limitations	20	Trial limitations, addressing sources of potential bias, imprecision, and, if relevant, multiplicity of analyses	12
Generalisability	21	Generalisability (external validity, applicability) of the trial findings	11-12
Interpretation	22	Interpretation consistent with results, balancing benefits and harms, and considering other relevant evidence	10-12
**Other information**			
Registration	23	Registration number and name of trial registry	2, 4
Protocol	24	Where the full trial protocol can be accessed, if available	N/A
Funding	25	Sources of funding and other support (such as supply of drugs), role of funders	2, 7

*We strongly recommend reading this statement in conjunction with the CONSORT 2010 Explanation and Elaboration[13] for important clarifications on all the items. If relevant, we also recommend reading CONSORT extensions for cluster randomised trials,[11] non-inferiority and equivalence trials,[12] non-pharmacological treatments,[32] herbal interventions,[33] and pragmatic trials.[34] Additional extensions are forthcoming: for those and for up-to-date references relevant to this checklist, see http://www.consort-statement.org.

Table:* CONSORT 2010 checklist of information to include when reporting a randomised trial
